# Unexpected significance of a minor reaction pathway in daytime formation of biogenic highly oxygenated organic compounds

**DOI:** 10.1126/sciadv.abp8702

**Published:** 2022-10-21

**Authors:** Hongru Shen, Luc Vereecken, Sungah Kang, Iida Pullinen, Hendrik Fuchs, Defeng Zhao, Thomas F. Mentel

**Affiliations:** ^1^ Department of Atmospheric and Oceanic Sciences & Institute of Atmospheric Sciences, Fudan University, Shanghai 200438, China.; ^2^ Institute of Energy and Climate Research, IEK-8: Troposphere, Forschungszentrum Jülich GmbH, 52425 Jülich, Germany.; ^3^ Physikalisches Institut, Universität zu Köln, 50932 Köln, Germany.; ^4^ Shanghai Frontiers Science Center of Atmosphere-Ocean Interaction, Fudan University, Shanghai 200438, China.; ^5^ Institute of Eco-Chongming (IEC), 20 Cuiniao Rd., Chongming, Shanghai 202162, China.; ^6^ IRDR ICoE on Risk Interconnectivity and Governance on Weather/Climate Extremes Impact and Public Health, Fudan University, Shanghai 200438, China.

## Abstract

Secondary organic aerosol (SOA), formed by oxidation of volatile organic compounds, substantially influence air quality and climate. Highly oxygenated organic molecules (HOMs), particularly those formed from biogenic monoterpenes, contribute a large fraction of SOA. During daytime, hydroxyl radicals initiate monoterpene oxidation, mainly by hydroxyl addition to monoterpene double bonds. Naturally, related HOM formation mechanisms should be induced by that reaction route, too. However, for α-pinene, the most abundant atmospheric monoterpene, we find a previously unidentified competitive pathway under atmospherically relevant conditions: HOM formation is predominately induced via hydrogen abstraction by hydroxyl radicals, a generally minor reaction pathway. We show by observations and theoretical calculations that hydrogen abstraction followed by formation and rearrangement of alkoxy radicals is a prerequisite for fast daytime HOM formation. Our analysis provides an accurate mechanism and yield, demonstrating that minor reaction pathways can become major, here for SOA formation and growth and related impacts on air quality and climate.

## Teaser

A minor reaction plays an unexpected major role in producing materials for daytime atmospheric organic particulate matter.

## INTRODUCTION

Secondary organic aerosols (SOAs) in the atmosphere affect air quality and climate by contributing a large portion to the mass of submicrometer particulate matter (*1*–*3*), by acting as cloud condensation nuclei, and by scattering and absorbing solar radiation (*4*–*9*). SOA is formed by condensation of products from the atmospheric oxidation of volatile organic compounds (VOCs) (*3*, *10*). Here, highly oxygenated organic molecules (HOMs) play a pivotal role (*11*–*17*) because of their low volatility. New particles can be formed from HOMs and be grown to sizes where they can act as cloud condensation nuclei. Elucidating HOM formation pathways is thus crucial to assess and predict the abundance of SOA and its impact on climate and air quality.

HOMs typically contain at least six oxygen atoms (*18*) and were first found in the reaction of alkenes with ozone (*11*–*13*), a common atmospheric oxidant, after several autoxidation steps (*18*, *19*) involving intramolecular H-shifts with subsequent O_2_ addition as shown in fig. S1. Bimolecular termination reactions of the HOM peroxy radicals lead to several product families C_Z_H_Y_O_X_, wherein we define a family as molecules having the same C_Z_H_Y_ backbone but a varying number X of O-atoms. In particular, biogenic monoterpenes are found to efficiently form HOM (*11*, *20*–*22*) and to contribute substantially to SOA formation (*11*–*13*, *20*, *23*) owing to their high emission rates (*24*). As shown for the most abundant monoterpene α-pinene (C_10_H_16_), also hydroxyl radicals (OH), the most important atmospheric oxidants, initiate fast HOM formation via a family of peroxy radicals (RO_2_•) containing 10 C-atoms and 17 H-atoms (C_10_H_17_O_X_•) (*25*–*27*). Here, we report a previously unidentified pathway for OH-induced HOM formation via C_10_H_15_O_X_•, intermediates with only 15 H-atoms as an alternative to previously proposed chemical pathways (*25*–*27*). Notably, on the basis of currently known formation pathways of HOM, such intermediates with 15 H-atoms are expected to be produced from only the reaction of α-pinene with ozone (*11*–*13*, *20*, *23*) or oxidation of α-pinene oxidation products by OH (*28*, *29*).

Only few studies have investigated HOM formation induced solely by OH radicals, although oxidation by OH radicals formed from the ozonolysis reaction itself inherently contributes to HOM formation. While HOM yields have been determined in the α-pinene oxidation by OH (*11*, *12*, *21*, *26*) and several HOM products have been reported, product distributions and formation mechanisms remain unclear. OH-initiated HOM formation was believed to start with OH addition to the C═C double bond of α-pinene forming peroxy radicals with 10 C-atoms and 17 H-atoms (C_10_H_17_O_3_•) (*25*–*27*). Berndt *et al.* (*26*) identified a number of HOM products formed via C_10_H_17_O_X_• peroxy radicals, related to each other by autoxidation steps. They proposed a mechanism where OH addition is followed by opening of the four-membered ring and forming a hydroxy peroxy radical containing a new C═C double bond in the six-membered ring (*29*, *30*). Recently, Xu *et al.* (*27*) calculated fast rates (>0.1 s^−1^) for unimolecular H-shifts in such RO_2_• radicals and proposed this pathway to be a major channel of HOM formation in the OH oxidation of α-pinene. However, only less oxygenated products (O ≤ 5) were characterized by Xu *et al.* (*27*), and although they suggested plausible routes to higher-oxygenated products (O > 5), the HOM formation hypothesis remains to be tested by systematic characterization of the HOM product distribution. In addition, HOM products from α-pinene + OH were mainly determined at very short reaction times [e.g., 3 to 7.9 s by Berndt *et al.* (*26*)], and therefore, products formed via fast sequences of bimolecular peroxy radical steps at the time scale of several tens to hundreds of seconds may still be missing. Thus, HOM formation in the ambient atmosphere, where the RO_2_• lifetimes can be long (up to several hundreds of seconds) (*31*, *32*), may involve alternative OH-initiated pathways. To our knowledge, systematic characterization of the HOM composition formed in the α-pinene oxidation by OH on a time scale of several minutes has not been reported and the underlying chemistry remains unexplored.

In this study, we investigated the OH-initiated oxidation of α-pinene under atmospherically relevant conditions in the outdoor SAPHIR chamber (Simulation of Atmospheric PHotochemistry In a large Reaction chamber) in experiments designed to minimize the impact of HOM production from ozonolysis reactions as demonstrated by direct OH radical and ozone measurements. We characterized the HOM composition in experiments at both low-NO [30 to ~100 parts per trillion (ppt)] and high-NO [~20 parts per billion (ppb)] mixing ratios representative for remote environments and areas influenced by anthropogenic activities, respectively. We further calculated the loss rates of different reaction pathways of peroxy radicals, the key intermediates in HOM formation, using measured HO_2_•, RO_2_•, and NO_X_ concentrations. Unexpectedly, more than 70% of C_10_-HOM from α-pinene + OH were related to C_10_H_15_O_X_• peroxy radicals in both low- and high-NO experiments in this study. We attribute these C_10_H_15_O_X_•-related C_10_-HOMs to be formed from the hydrogen abstraction pathway by OH rather than from the previously suggested addition pathway of OH as the first step in the oxidation of α-pinene by OH. We propose a plausible pathway of OH-initiated HOM formation, which is supported by theoretic kinetics calculations. H-abstraction pathways have been proposed for monoterpenes (*28*, *33*–*36*) contributing typically about 10% to oxidation by OH; therefore, they are usually not included in atmospheric chemical models as being of minor interest.

## RESULTS

### HOM product distribution

**Fig. 1.**
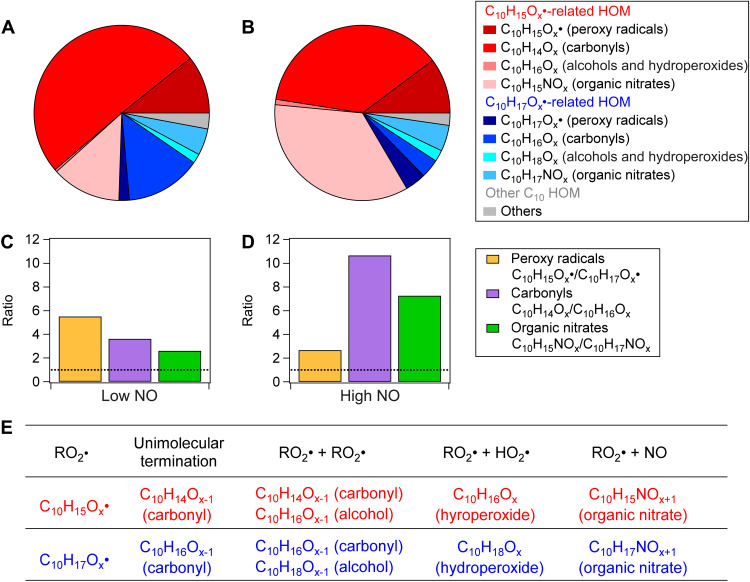
C_10_-HOM from OH oxidation of α-pinene in low-NO (30 to ~100 ppt) and high-NO (~20 ppb) experiments. The distribution of C_10_H_15_O_X_•-related HOM (red), C_10_H_17_O_X_•-related HOM (blue), and other C_10_-HOM (gray) from α-pinene + OH during the first 15-min reaction time in (**A**) low-NO and (**B**) high-NO experiments. Four families, including peroxy radicals, carbonyls, alcohols and hydroperoxides, and organic nitrates, were identified in both C_10_H_15_O_X_•- and C_10_H_17_O_X_•-related C_10_-HOM. Concentration ratios of these C_10_H_15_O_X_•- to C_10_H_17_O_X_•-related families under (**C**) low NO and (**D**) high NO are denoted in bar charts, with peroxy radicals in yellow, carbonyls in purple, and organic nitrates in green. Note that concentrations of the family of alcohols and hydroperoxides in both C_10_H_15_O_X_•- and C_10_H_17_O_X_•-related HOM are negligible and are thus not shown in the figure. The horizontal dashed lines in the bar charts represent equal contribution (ratio 1). The details of determining the ratio of C_10_H_15_O_X_•-related to C_10_H_17_O_X_•-related carbonyl are provided in section S3. (**E**) Possible reactions leading to C_10_ closed-shell products (carbonyls, organic nitrates, and alcohols and hydroperoxides) are listed for individual peroxy radicals, including C_10_H_15_O_X_• (red) and C_10_H_17_O_X_• (blue).

In the chamber experiments of OH oxidation of α-pinene, a C_10_H_15_O_X_•-related HOM chemistry, including both peroxy radicals and their related termination products, dominated over the expected C_10_H_17_O_X_•-related chemistry at both low NO (NO: 30 to ~100 ppt; Fig. 1A) and high NO (NO: ~20 ppb; Fig. 1B). On the basis of the established HOM chemistry (*18*, *37*), C_10_H_15_O_X_• peroxy radicals are expected to be terminated by self-OH elimination or reactions with other RO_2_•, NO, or HO_2_• to form C_10_ closed-shell products with 14 H-atoms (likely carbonyls), 15-H-atoms (organic nitrates), or 16 H-atoms (alcohols and hydroperoxides) as shown in Fig. 1E and fig. S2. On the basis of the measured concentrations of RO_2_•, HO_2_•, and NO, an average bimolecular RO_2_• loss rate of ~0.02 s^−1^ (low NO) and ~ 3.5 s^−1^ (high NO) predominately due to the reaction with NO was estimated in our previous study (*38*). The expected main C_10_ closed-shell product families of C_10_H_15_O_X_• (X = 6 to 14) are the carbonyls C_10_H_14_O_X_ (X = 6 to 16) and organic nitrates C_10_H_15_NO_X_ (X = 6 to 15; Fig. 1A). Analogously, C_10_H_17_O_X_• (X = 7 to 14) forms the carbonyl family with formulas C_10_H_16_O_X_ (X = 6 to 16) and the organic nitrate family C_10_H_17_NO_X_ (X = 6 to 14; Fig. 1B). Note that in the following text, “X” will be used as defined here when one product family is referred to. The relative abundances of each individual product are shown in fig. S6. Other possible C_10_ closed-shell products of C_10_H_15_O_X_• and C_10_H_17_O_X_• are listed in Fig. 1E. Note that products with formulas C_10_H_16_O_X_ can arise either from C_10_H_15_O_X_• peroxy radicals as alcohols or hydroperoxides or from C_10_H_17_O_X_• as carbonyls, while all other families can uniquely be attributed to one peroxy radical family. The separation of C_10_H_15_O_X_• and C_10_H_17_O_X_• chemistry’s contributions to C_10_H_16_O_X_ is discussed in section S3. During the early stage of the reaction time (0 to 15 min), the sum of peroxy radical concentrations of the C_10_H_15_O_X_• family was more than twice as much as that of the C_10_H_17_O_X_• family regardless of the NO concentration (Fig. 1, C and D). We also observed higher concentrations of C_10_ termination product families related to C_10_H_15_O_X_• compared to C_10_H_17_O_X_•. Concentrations of carbonyls (C_10_H_14_O_X_) and organic nitrates (C_10_H_15_NO_X_) stemming from C_10_H_15_O_X_• radicals are 3.5 and 2.5 times higher than those of their counterparts C_10_H_16_O_X_ and C_10_H_17_NO_X_ from C_10_H_17_O_X_• at low NO (Fig. 1C), and 10.7 and 6.9 times higher at high NO (Fig. 1D). Note that concentrations of alcohol and hydroperoxide products of C_10_H_16_O_X_ and C_10_H_18_O_X_ from respective reactions of C_10_H_15_O_X_• and C_10_H_17_O_X_• radicals are negligible. Both the predominant contribution of the C_10_H_15_O_X_• peroxy radicals themselves and of the related C_10_H_14_O_X_ and C_10_H_15_NO_X_ HOM demonstrate the leading role of the C_10_H_15_O_X_• peroxy family in the OH-induced HOM formation from α-pinene at low and high NO.

In this study, we assume that C_10_H_15_O_X_•- and C_10_H_17_O_X_•-related HOM have the same measurement sensitivity in NO_3_^−^-CIMS. The rationale is that autoxidation provides two or more hydrogen bond donors (-OOH or -OH) in C_10_H_15_O_X_•- and C_10_H_17_O_X_•-related HOM with six and more oxygen atoms (*18*, *37*). These H bond donors allows HOMs to form strong clusters with NO_3_^−^, with rates near the collision limit similar to H_2_SO_4_ as they are able to compete with the very stable (HNO_3_)NO_3_^−^, thus leading to the same high sensitivity (*39*). Moreover, the chemical structures of radicals, and carbonyls and organic nitrates with the same number of O atoms arising from C_10_H_15_O_X_• or C_10_H_17_O_X_•, only differ by one C═C bond or an endocyclic peroxide ring. Such differences are unlikely to substantially change the multiple H bonding–based sensitivity of NO_3_^−^-CIMS to C_10_H_15_O_X_•-related families and C_10_H_17_O_X_•-related families (*40*). Experimentally, Pullinen *et al.* (*41*) used the same NO_3_^−^-CIMS with the same setting and found no dependence of sensitivity on the functional groups of HOM, within a maximum uncertainty of a factor of 2. Therefore, we conclude that the sensitivity of NO_3_^−^-CIMS to C_10_H_15_O_X_•- and C_10_H_17_O_X_•-related HOM is the same and near the collision limit. Applying the uncertainty factor of 2 to the C_10_H_17_O_X_•-related families, concentration ratios became C_10_H_14_O_X_•:C_10_H_16_O_X_• = 1.8 (low NO) and 5.4 (high NO), and C_10_H_15_NO_X_:C_10_H_17_NO_X_ = 1.2 (low NO) and 3.5 (high NO). Even within this uncertainty, the role of C_10_H_15_O_X_• chemistry remains important in the HOM formation from OH oxidation of α-pinene.

At low NO, a strong contribution of the C_10_H_15_O_X_• family is also evident in the abundance of accretion products with 20 C-atoms (C_20_-HOM). C_20_-HOMs are formed via the self- and cross-reactions (*42*, *43*) of C_10_H_15_O_X_• and C_10_H_17_O_X_• peroxy radicals via the loss of two O-atoms. We observed C_20_-HOM, with a concentration ratio for C_20_H_30_O_X_ (X = 6, 9 to 17):C_20_H_32_O_X_ (X = 9 to 17):C_20_H_34_O_X_ (X = 10 to 14) of 1:2.2:1.6 within the first 15 min of the reaction of α-pinene + OH (fig. S9C). Considering the possible permutations, accretion products C_20_H_30_O_2n-2_ and C_20_H_34_O_2n-2_ are formed solely by C_10_H_15_O_n_• or C_10_H_17_O_n_•, respectively, while cross-reactions of C_10_H_15_O_n_• and C_10_H_17_O_n_• lead to C_20_H_32_O_2n-2_. Note that the concentration ratios of HOM C_10_H_15_O_X_• to C_10_H_17_O_X_• need not exactly match the corresponding dimer ratios, C_20_H_30_O_X_ to C_20_H_34_O_X_, because less oxygenated C_10_H_15_O_X_• to C_10_H_17_O_X_• also participate in accretion reactions as shown in previous studies (*15*, *41*, *42*, *44*). The dominance of C_10_H_15_O_X_• observed for HOM (X > 6) is not necessarily applicable to less oxygenated compounds, where indeed C_10_H_17_O_X_• should dominate in OH oxidation of α-pinene. Therefore, observation and concentration ratios of C_20_H_34_O_X_- and C_20_H_32_O_X_-HOM here do not contradict our finding of the dominance of C_10_H_15_O_X_•-related products in C_10_-HOM. In contrast, the strong presence of the “mixed” C_20_H_32_O_X_ family and the observation of the C_20_H_30_O_X_ family underline the central role of the C_10_H_15_O_X_• peroxy radicals. At high NO (fig. S4B), accretion products are negligible, because reactions with NO suppress their formation (*41*, *45*–*47*).

We also observed substantial fractions of C_7_-HOM, which are expected to arise from bond scission reactions of alkoxy radicals. We attribute their origin to the C_10_H_15_O_X_• and C_10_H_17_O_X_• families with details being discussed in section S6. The strong C_7_-HOM product formation and the dominance of C_7_H_9_O_X_• (originating from C_10_H_15_O_X_• peroxy radicals)–related products over C_7_H_11_O_X_• (originating from C_10_H_17_O_X_• peroxy radicals)–related products are also in support of the dominance of a C_10_H_15_O_X_• peroxy radical chemistry, as well as indicative of the importance of pathways involving alkoxy radicals. As we will show in the following, alkoxy radicals play a central role in OH-induced pathways to C_10_H_15_O_X_• peroxy radicals from α-pinene.

### OH-induced pathways to C_10_H_15_O_x_•

Addition of OH to the double bond of α-pinene (C_10_H_16_) is its accepted major oxidation pathway [~90%; (*26*, *48*)] and results in peroxy radicals C_10_H_17_O_3_• with 17 H-atoms. Subsequent autoxidation leads to peroxy radicals with formulas C_10_H_17_O_X_• (fig. S12A). This is not compatible with our observations on 15-min time scales. We propose that the pathway of hydrogen abstraction by OH forms C_10_H_15_O_2_• after O_2_ addition (*28*, *29*), a peroxy radical that has 15 H-atoms like the observed HOM. C_10_H_15_O_2_• is then the starting point for forming the C_10_H_15_O_X_• peroxy radical family by autoxidation steps.

An alternative pathway forming C_10_H_15_O_X_• peroxy radicals can start from a first-generation C_10_ oxidation products in α-pinene + OH reaction. We consider pinonaldehyde (C_10_H_16_O_2_) (*34*) as the most competitive candidate, which is one of the main products in α-pinene + OH reaction and the most abundant first-generation C_10_ product reported (fig. S12, B and C). H-abstraction by OH and subsequent O_2_ addition leads to C_10_H_15_O_4_•, which could also be a starting point to form the C_10_H_15_O_X_• family by further autoxidation steps. However, on the 15-min time scale of our experiments, the pinonaldehyde product has not yet accumulated and the rate of the H-abstraction reaction from α-pinene by OH is much faster compared to pinonaldehyde (at least 70 and 8 times faster at low and high NO, respectively; see section S7). As pinonaldehyde is the most abundant first-generation C_10_ product and reacts fast with OH (3.9 × 10^−11^ cm^3^ molecule^−1^ s^−1^ at 298 K), other C_10_ first-generation products are expected to be even less important in forming C_10_H_15_O_X_• because of lower yield and reactivity, or the inability to produce C_10_H_15_O_X_• when reacting with OH, such as C_10_-hydroxynitrate. Therefore, we conclude that direct hydrogen abstraction by OH from α-pinene is the responsible pathway to C_10_H_15_O_X_• peroxy radicals in this study.

**Fig. 2.**
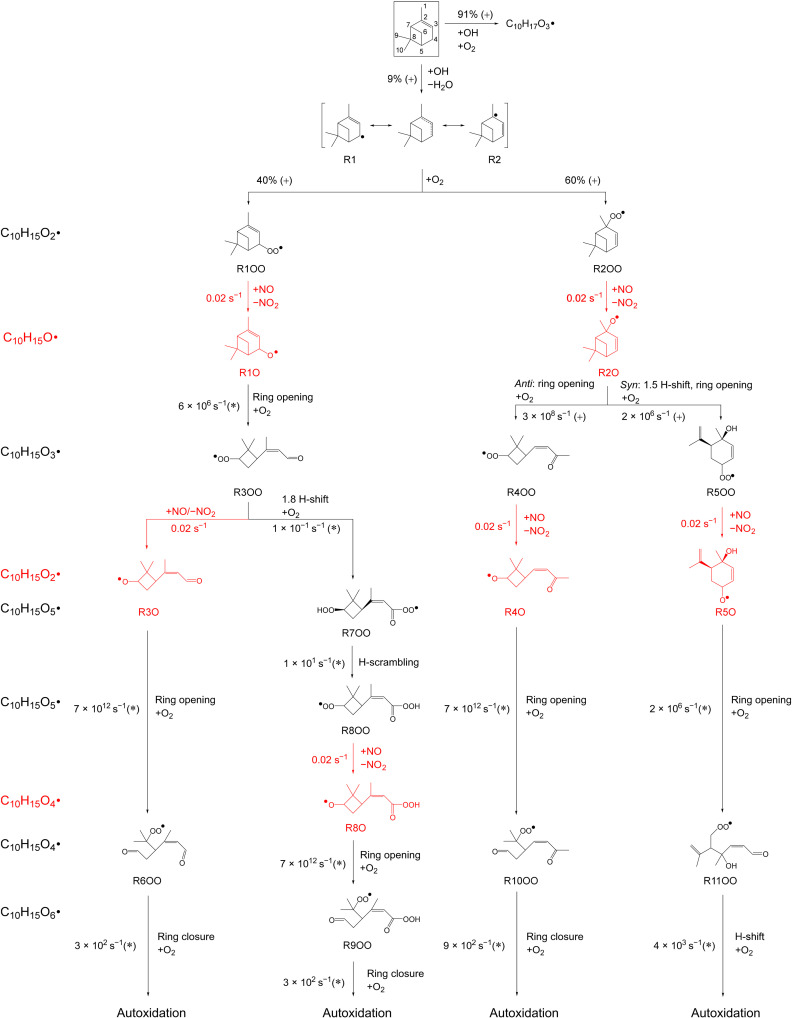
Formation pathways to C_10_H_15_O_X_• radicals and their structures. Simplified reaction scheme at 298 K after H-abstraction from α-pinene (shown in the box) by OH leading to C_10_H_15_O_X_• radicals that can undergo further autoxidation. Only pathways leading to HOM formation are included in this figure; other competing pathways are described in sections S9 and S10. Alkoxy steps are highlighted in red. Alkyl radicals are labeled as R#, while alkoxy radicals and peroxy radicals are labeled as R#O and R#OO. Chemical formulas are denoted on the left side of the figure. Rate of alkoxy radical formation in the RO_2_• + NO reactions is estimated on the basis of 100 ppt of NO and an organic nitrate yield of 0.1. Unimolecular rate coefficients are derived from structure-activity relationships (SARs) (*) or taken from Peeters *et al.* (*28*) (+). See the Supplementary Materials for more detailed stereo-specific chemistry, further autoxidation steps, and minor reactions with rates derived from SARs or explicit theoretical calculations.

The most likely routes to form C_10_H_15_O_2_• peroxy radicals subsequent to the hydrogen abstraction from α-pinene by OH shown in Fig. 2 are based on calculated rate constants (*28*). The initial hydrogen abstraction is expected to occur on C4, leading to an allylic radical with two resonance radical sites, R1 and R2, which immediately react with O_2_ forming the peroxy radicals R1OO and R2OO with an estimated ratio of 40:60. These RO_2_• have no competitive unimolecular reaction channels (table S1 and section S9). Under the experimental conditions with the presence of NO, R1OO and R2OO thus mainly undergo reactions with NO (as shown in fig. S7) and form the alkoxy radicals R1O and R2O (and organic nitrates, not shown in the Fig. 2). In the following, we denote the conversion of RO_2_• to alkoxy radicals (RO•) by NO (RO_2_• + NO ➔ RO• + NO_2_) as an “alkoxy step.” For R1O, the opening of the six-membered ring followed by O_2_ addition leads to R3OO, which is unlikely to proceed to autoxidation because of the remaining four-membered ring (table S2 and section S10). However, peroxy radicals formed after one further alkoxy step, followed by ring cleavage and O_2_ addition, are expected to easily undergo autoxidation (e.g., R6OO; fig. S18A). The competing 1,8 H-shift of the aldehydic H-atom in R3OO followed by one more alkoxy step can also lead to a peroxy radical, R9OO, which likewise can undergo fast autoxidation steps as shown in fig. S18B. Depending on the stereo-specific chemistry of the alkoxy radicals R2O (see fig. S15 and section S10), these will either undergo direct opening of the six-membered ring and form the peroxy radical R4OO while retaining the four-membered ring [about 60% (*28*)] or undergo 1,5 H-shift and subsequent cleavage of the four-membered ring R5OO (about 40%). According to our calculations, R4OO and R5OO have no viable unimolecular reactions to further react via an autoxidation step (table S2). Again, one more alkoxy step allows the opening of the remaining four- or six-membered ring in R4O and R5O with subsequent O_2_ addition (Fig. 2) to proceed to autoxidation via R10OO and R11OO (see Fig. 2, and subsequent chemistry in fig. S18, C and D). R10OO and R11OO are predicted to react further with high-rate coefficients, for example, 8.7 × 10^2^ s^−1^ for the five-membered ring closure of R10OO and 4 × 10^3^ s^−1^ for the 1,8 H-shift of R11OO derived from structure-activity relationship (SAR), as described in section S11. This is related to the functionalization of R10OO and R11OO by the double bond (*49*) and the aldehyde group (*50*).

**Fig. 3.**
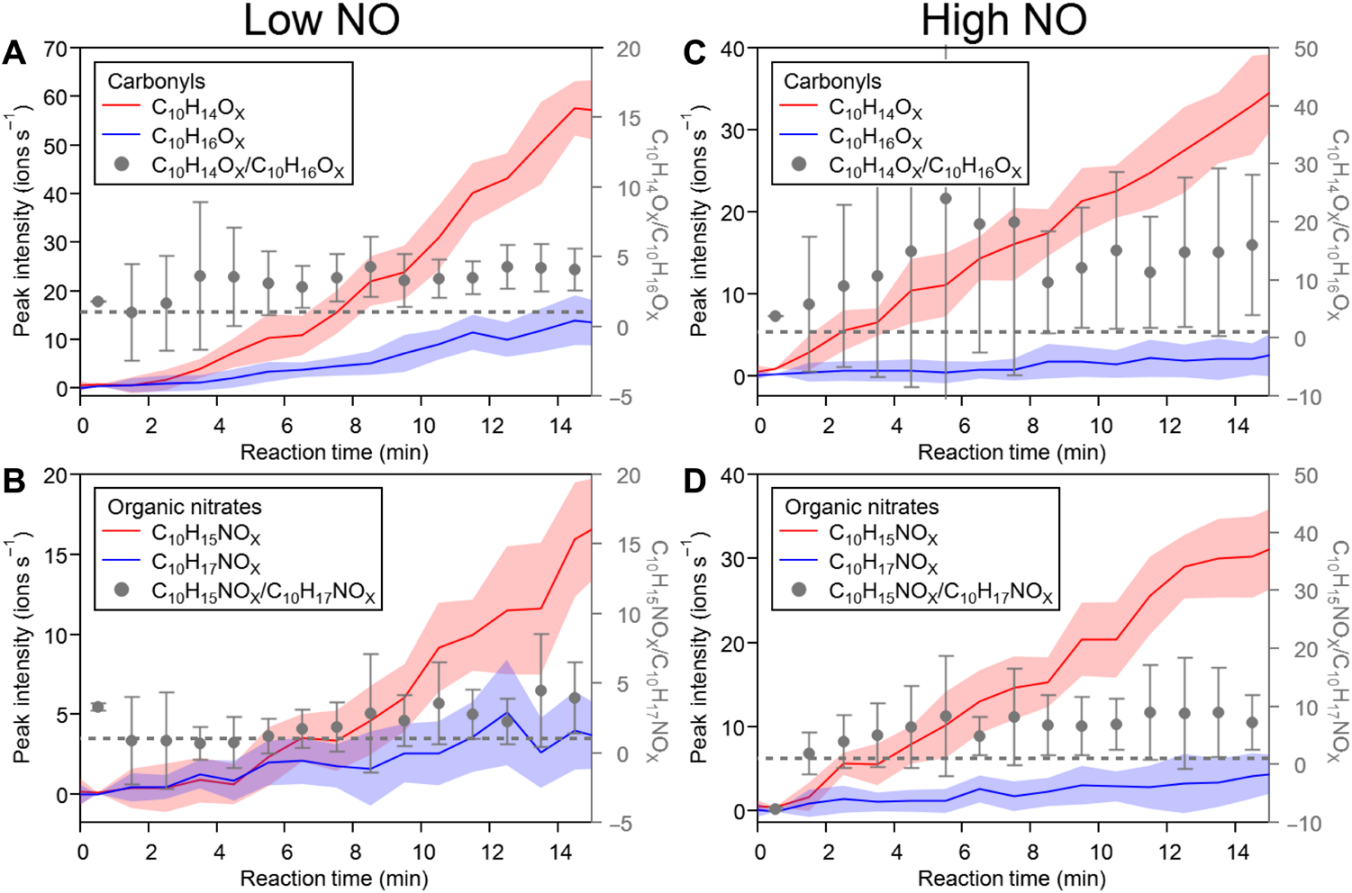
Time series of C_10_H_15_O_X_•- and C_10_H_17_O_X_•-related families of carbonyls and organic nitrates in low-NO (30 to ~100 ppt) and high-NO (~20 ppb) experiments. For the carbonyl family, C_10_H_14_O_X_ (red), C_10_H_16_O_X_ (blue), and their concentration ratio (C_10_H_14_O_X_ to C_10_H_16_O_X_, gray) during the first 15-min reaction are shown in (**A**) low-NO and (**C**) high-NO experiments. Time series of the family of organic nitrates, including C_10_H_15_NO_X_ (red), C_10_H_17_NO_X_ (blue), and concentration ratio of C_10_H_15_NO_X_ to C_10_H_17_NO_X_, are similarly demonstrated in (**B**) low-NO and (**D**) high-NO experiments. The horizontal dashed lines represent a concentration ratio of 1, where C_10_H_15_O_X_• chemistry is equal to C_10_H_17_O_X_• chemistry. All data are averaged to a time resolution of 1 min. In each panel, error bars of C_10_H_15_O_X_•- and C_10_H_17_O_X_•-related products (left axes) represent 1 SD and error bars of concentration ratios (right axes) were calculated using error propagation.

As shown in Fig. 2, alkoxy steps allow for the formation of RO•, which will undergo ring opening by α-scission at very fast rates. Ring opening, especially of the bicyclic frame of α-pinene and ultimately of both rings, is required to remove geometric strain in the molecule. Once migratable H-atoms are accessible, multistep autoxidation reactions lead to the formation of the C_10_H_15_O_X_• peroxy radical family. In this study, RO• are mainly formed by the alkoxy step (RO_2_• + NO ➔ RO• + NO_2_), whose reaction rate depends on the current NO concentration, while the ring opening is especially fast with a typical rate >10^6^ s^−1^ (*51*). As a consequence, the formation rate of C_10_H_15_O_X_•-related HOM is mainly affected by the NO concentration. In our low-NO experiments, NO needed first to be formed by photolysis of HONO originating from the chamber walls (*52*). Note that the NO concentration increased gradually during the experiment at low NO (fig. S3A). The formation of C_10_H_15_O_X_•-related products was consequently delayed by 3 to 4 min (Fig. 3A) because of the lack of fast RO• production from alkoxy steps at low NO concentration (30 to 50 ppt within the first 5-min reaction time). As the experiment proceeded, increasing NO accelerated the RO• production and the subsequent formation of C_10_H_15_O_X_•-related carbonyls C_10_H_14_O_X_ (Fig. 3A) and organic nitrates C_10_H_15_NO_X_ (Fig. 3B). In the high-NO experiment, with NO concentrations of ~20 ppb (RO_2_• lifetime of ~0.2 s), C_10_H_15_O_X_•-related carbonyls and nitrates form immediately (<1 to 2 min; Fig. 3, C and D) because high concentrations of NO directly enabled the alkoxy steps. A similar time series pattern, i.e., 3 to 4 min delay at low NO and quick formation at high NO, was also observed in individual C_10_H_15_O_X_•-related C_10_-HOM, with several examples shown in fig. S8. Time series of both lumped and individual C_10_H_15_O_X_•-related C_10_-HOM are consistent with the role of NO in their formation and further support the proposed mechanism in Fig. 2, with rapid autoxidation after two alkoxy steps. At low NO, the autoxidation process of C_10_H_17_O_X_• is competitive with bimolecular reactions with NO, with rapid H shifts (>0.1 s^−1^) proposed in Xu *et al.* (*27*). Because the formation rate of C_10_H_17_O_X_• peroxy radicals is independent of NO (*25*–*27*), time series of C_10_H_17_O_X_•-related C_10_-HOM are similar at low and high NO, with no delay at low NO observed, as shown in blue lines in Fig. 3 and fig. S8 (G to L). Increasing concentration ratios of C_10_H_15_O_X_•-related C_10_-HOM to C_10_H_17_O_X_•-related C_10_-HOM (Fig. 3, A and B) at low NO further support an accelerated C_10_H_15_O_X_• chemistry by increasing NO as the reaction proceeds and a C_10_H_17_O_X_• chemistry independent of NO, likely due to the absence of double bond (*53*) and aldehyde groups (*50*) compared to C_10_H_15_O_X_•. At high NO, concentration ratios of C_10_H_15_O_X_•-related C_10_-HOM to C_10_H_17_O_X_•-related C_10_-HOM (Fig. 3, C and D) quickly reached a relatively stable value, where high NO concentrations allow rapid alkoxy steps and make C_10_H_15_O_X_• chemistry dominant during the first 15-min reaction time. Moreover, concentration ratios of C_10_H_15_O_X_•-related C_10_-HOM over C_10_H_17_O_X_•-related C_10_-HOM are higher at high NO (~10) than at low NO (~2), also indicating the important role of NO in the formation of C_10_H_15_O_X_•-related C_10_-HOM from OH oxidation of α-pinene. The observed time series of C_10_H_15_O_X_•-related C_10_-HOM to C_10_H_17_O_X_•-related C_10_-HOM in Fig. 3 are generally similar to modeled results (fig. S22) after adding C_10_H_17_O_X_• chemistry (*27*) to MCM (Master Chemical Mechanism). Detailed descriptions are given in section S12; briefly, we followed C_10_H_17_O_X_• chemistry by Xu *et al.* (*27*) and incorporated full reactions leading to highly oxygenated products (O > 6) into our MCM calculations. For missing branching ratio and rate coefficients, uniform values were applied according to empirical knowledge from literature (*50*, *54*–*56*). Although the modeled concentration ratios of C_10_H_15_O_X_•-related HOM to C_10_H_17_O_X_•-related HOM match the trend of the observed ones well, they did not exactly reproduce the time scale of the initial increase at high NO (Fig. 3 and fig. S22). This may be attributed to the fact that the C_10_H_15_O_X_• HOM chemistry proposed in this study is still not complete, as only the most likely formation pathways were included, and some important pathways may be missing, particularly alkoxy-peroxy pathways for highly oxygenated RO_2_• (O > 6). Similarly, C_10_H_17_O_X_• HOM chemistry included in our MCM model study is also not complete, as many rate constants are not available in the literature. Similar patterns to C_10_-HOM were observed for the time series of C_7_-HOM at both low and high NO, which is discussed in detail in the Supplementary Materials (section S6). In all cases, C_10_H_15_O_X_•-related products became dominant over C_10_H_17_O_X_•-related products after 3 to 4 min or 1 to 2 min at low and high NO, respectively (Fig. 3 and fig. S11), underlining the importance of the C_10_H_15_O_X_• peroxy radical family for HOM formation.

Compared to the pathways involving the C_10_H_17_O_X_• peroxy radical family (*25*–*27*), our mechanism for the OH-induced formation of the C_10_H_15_O_X_• peroxy radical family includes the minor H-abstraction channel as a rate-limiting step as well as a minimum of two bimolecular reaction steps. The question arises whether the bimolecular reaction steps are sufficiently fast to explain the observed HOM formation. We applied the mechanism presented in Fig. 2 in model calculations using explicit reaction rate constants for the individual peroxy radicals and calculated the time evolution of C_10_H_15_NO_9_ as an example (see details in section S12). It appeared that our mechanism indeed explains the observed C_10_H_15_NO_9_ concentrations in both the low- and high-NO experiments (fig. S19 and section S12). The calculations reproduce the delay period in the low-NO experiment and the accelerated C_10_H_15_NO_9_ formation at high NO. The delayed formation time at low NO is indeed related to the alkoxy production rate as shown in fig. S20. The sensitivity of the model results to the model parameters is also discussed in more detail in the Supplementary Materials.

We conclude that the H-abstraction channel with subsequent alkoxy steps is sufficient to explain an OH-induced C_10_H_15_O_X_•-related HOM chemistry on minute time scales. We further conclude that OH-induced HOM formation (from α-pinene) must be preceded by the formation and intramolecular rearrangements of alkoxy radicals to open the ring structures and thereby enabling intramolecular H-shifts in peroxy radicals (autoxidation steps).

The formation of the C_10_H_15_O_X_• peroxy radical family and the uniquely related HOM families C_10_H_14_O_X_ and C_10_H_15_NO_X_ have been reported before in α-pinene + OH experiments. A dominance of a C_10_H_15_O_X_•-based over a C_10_H_17_O_X_•-based chemistry was also observed by Kang (*57*) and Pullinen *et al.* (*41*) at low and high NO_X_, although the HOM chemistry may differ as their experiments contained O_3_ and were conducted in the continuous stirred tank reactor JPAC under steady-state conditions with typical reaction times of several tens of minutes. In the same sense, Yan *et al.* (*46*) observed similar C_10_H_15_O_X_•-related products in the CLOUD chamber, including C_7_H_8_O_X_ and C_7_H_9_NO_X_ and accretion products C_20_H_30_O_X_ and C_20_H_32_O_X_, although the contribution of ozonolysis of α-pinene to the formation of these compounds cannot be ruled out. Other laboratory studies (*25*–*27*) on HOM from α-pinene + OH reported only the formation of C_10_H_17_O_X_•-related products. The missing C_10_H_15_O_X_• products may be attributed to the different experimental conditions. For example, at very short reaction times of 2.6 to 7.9 s (*25*, *26*), the importance of alkoxy formation from bimolecular reactions may be reduced (with an estimated bimolecular RO_2_• lifetime of 1.7 min at low NO), and the OH addition channel dominates the HOM formation via C_10_H_17_O_X_• peroxy radicals. This is not in contradiction to our study, where also in the first 1 to 2 min at low NO, C_10_H_17_O_X_•-based products are most abundant, consistent with the findings of Berndt *et al.* (*26*). Varying the concentrations of NO, Berndt (*25*) still only observed C_10_H_17_O_3–7_• using C_2_H_5_NH_3_^+^ and I^−^ reagent ions. While the reason for the absence of C_10_H_15_O_X_• and C_10_H_17_O_>7_• is not completely clear, there may be two possible reasons. First, the concentrations of C_10_H_15_O_X_•-related C_10_-HOM may be relatively low, particularly C_10_H_15_O_<8_• (fig. S6) due to fast H shifts and the reaction time (7.5 s) in the experiments of Berndt (*25*). This is consistent with the likely dominant role of C_10_H_17_O_X_• chemistry in the low oxygenated molecules (O < 6) from OH addition channel but the important C_10_H_15_O_X_• chemistry in the formation of HOM with six and more oxygen atoms. In addition, the peaks of C_10_H_15_O_X_• in mass spectra are very close to C_10_H_17_NO_X_ (differing by 0.024 Th), which may be thus covered in the peaks of C_10_H_17_NO_X_. Second, although C_2_H_5_NH_3_^+^ ionization is generally sensitive to detect oxygenated compounds with a wide range of oxygenation, it may not be sensitive to every HOM as shown for products in the reaction of isoprene with OH (*58*). I^−^ ionization has been shown to be not sensitive to more oxygenated organics (*59*). As discussed above, it is unlikely that the same ionization scheme [here, NO_3_^−^ reagent ions in an Eisele type inlet (*39*, *40*, *60*, *61*)] biases C_10_H_15_O_X_•-related over C_10_H_17_O_X_•-related compounds as both families should carry similar functional groups (for the same number of O-atoms) despite the different number of H-atoms.

Last, HOM products based on the C_10_H_15_O_X_• family have been observed in the reaction of α-pinene with Cl•-atoms, which has a stronger preference to abstract a hydrogen atom from α-pinene compared to OH (*62*). Recent α-pinene + Cl• laboratory studies have found monomers of the C_10_H_14_O_X_ family and C_20_H_30_O_X_ accretion products as main HOM products and attributed those to the hydrogen abstraction channel. However, the site specificity is still unknown (*63*, *64*). This finding provides strong support that HOM can be formed indeed from a hydrogen abstraction channel of α-pinene.

### HOM molar yields of OH induced C_10_H_15_O_X_•-related formation pathways

The C_10_H_15_O_X_•-related HOM molar yields from OH oxidation of α-pinene were estimated to be 0.8% (−0.4%/+1.2%) at low NO and 0.7% (−0.4%/+1.0%) at high NO, accounting for about 70 and 82% of the total HOM yield, respectively. The total HOM yield, 1.1% (−0.6%/+1.6%) at low NO and 0.8% (−0.4%/+1.2%) at high NO, is roughly within the range of reported values 0.05 to 1.5% [based on the measurements with a nitrate CIMS (*11*, *12*, *21*, *26*) in table S5], although the chemistry may differ in these different experiments. This yield is lower than the HOM yield measured using acetate and ethylaminium ionization schemes (table S5) (*25*, *26*, *65*). The yields were calculated for the first 15-min reaction time and include wall loss and dilution corrections (see details in Materials and Methods). Note that the HOM yields here using the calibration coefficient derived from H_2_SO_4_ (*58*) represent lower limit values with an uncertainty of a factor of ~2. The slightly lower molar yields at high NO concentrations may be attributed to fast alkoxy fragmentation processes leading to small molecules (*51*), which can only be measured with limited sensitivity by the nitrate CIMS instrument (*39*, *40*).

## DISCUSSION

Our study provides clear evidence that the hydrogen abstraction channel in the α-pinene + OH reaction dominates the HOM formation via C_10_H_15_O_X_• on time scales of several minutes, at both low-NO (30 to ~100 ppt) and high-NO (~20 ppb) mixing ratios. This indicates that the HOM formation takes another route other than the major route via the OH addition channels (e.g., MCM v.3.1.1). HOM formation initiated by H-abstraction by OH radicals can explain the HOM product distribution found in the ambient atmosphere in previous studies. For example, on the basis of the source apportionment analysis of time-resolved HOM mass spectra, Yan *et al.* (*66*) isolated a daytime factor containing C_10_H_15_NO_8_ as the major peak in Hyytiälä, a forest site in north Finland. This was supposed to be controlled by OH oxidation of monoterpenes although some contribution of ozonolysis could not be excluded. Jokinen *et al.* (*67*) suggest photochemical production pathways involving the OH oxidation for C_10_H_15_NO_8_ and C_10_H_15_NO_9_ in Hyytiälä because of their considerable decreased concentrations during a solar eclipse. Field results (*68*) from the Southern Oxidant and Aerosol Study campaign showed a main peak concentration of C_10_H_15_NO_7–11_ after sunrise when the concentrations of NO were high and O_3_ concentrations were low, with an estimated C_10_H_17_NO_6–9_–to–C_10_H_15_NO_7–11_ concentration ratio of ~1:2. If these products were formed by the OH addition as previously thought, the major peak should be C_10_H_17_NO_6–9_ rather than C_10_H_15_NO_7–11_.

HOM from hydrogen abstraction by OH can be an important source of HOM in areas with high monoterpene emission rates. Pye *et al.* (*69*) found that OH dominates monoterpene loss rates in many regions such as Europe, a large part of the United States, and China [fig. S9D in the Supporting Information (*69*)]. The HOM production rate by OH via the hydrogen abstraction channel could reach up to 25 to 30% of the total C_10_H_15_O_X_•-related HOM production rate by OH and O_3_, calculated by using average concentrations of 2 × 10^6^ and 7 × 10^11^ molecule cm^−3^ for OH and O_3_ (*70*) and respective molar yields of 0.7 to 0.8% for OH-induced HOM (this study) and 2.9 to 3.4% for O_3_-induced HOM (*12*, *21*). In regions with high OH concentrations of up to 1.5 × 10^7^ cm^−3^ such as Southern China (*71*), isomerization reactions of alkoxy radicals from the hydrogen abstraction channel can contribute to 71 to 76% of the production rate of the C_10_H_15_O_X_•-related HOM from both OH and O_3_. Hence, the OH oxidation pathway via the hydrogen abstraction channel is not negligible in the formation of HOM; in particular, highly oxygenated organic nitrates of the C_10_H_15_NO_X_ family were observed in the above field studies (*66*, *68*) and may play a potentially competitive or even dominant role in the OH-rich atmosphere. The hydrogen abstraction by OH may also be important for HOM formation from other monoterpenes (*35*, *72*) as well as from other unsaturated VOCs if ring opening is a key prerequisite.

Because HOM can account for a substantial mass fraction of SOA, especially in the early growth of particles in new particle formation events, the hydrogen abstraction channel should be taken into account in numerical models to accurately simulate HOM and SOA concentrations and predict new particle formation and SOA growth, particularly in OH-rich areas (*69*), such as the Southeastern United States and Southern China. However, current chemical mechanisms, such as α-pinene + OH reactions in MCM v3.1.1, ignore the hydrogen abstraction channel and typically only include OH addition pathways, leading to C_10_H_17_O_X_•-related first-generation products as mentioned above. This simplification is mainly due to the low branching ratio (~10%) of hydrogen abstraction compared to that of the OH addition channel (90%) (*29*). Our study indicates that a minor initial reaction pathway can be an important or even dominant pathway for selected processes like HOM formation. Through HOM, the minor H-abstraction channel unexpectedly gains in importance for processes related to submicrometer aerosols, like new particle formation and SOA growth, with consequences for all kinds of impacts of submicrometer aerosols, e.g., activation of cloud condensation nuclei and, thus, climatic impact of aerosols.

Our study also highlights the role of alkoxy radicals in HOM formation besides autoxidation. As NO_X_ is ubiquitous in the ambient atmosphere, forming alkoxy radicals is common as RO_2_• + NO is expected to be a dominant loss path of peroxy radicals in urban areas and in most of the remote regions on the continent because of the wide influence of anthropogenic emissions on the planet except for very remote areas such as Amazonia over certain seasons. In addition, other reactions of RO_2_• such as with RO_2_• or HO_2_• may also form alkoxy radicals. Because of their fast unimolecular reactions such as ring opening, alkoxy steps can facilitate fast autoxidation forming HOM that would be otherwise not possible (*73*). The role of alkoxy radical in HOM formation in other reaction systems (*74*) such as for other VOCs and other oxidants, and in the ambient atmosphere, warrants further studies.

## MATERIALS AND METHODS

### Experimental procedure and instrumental setup

The experiments were conducted in the SAPHIR chamber (August 2013) (*75*) under representative environmental conditions of low NO (30 to ~100 ppt) and high NO (~20 ppb) concentrations. Note that the “low” and “high” designators here are solely used to distinguish the NO concentrations in these two types of experiments, as RO_2_• will mainly react with NO in both cases. Details of the RO_2_• fate in our study are explicitly derived from measured RO_2_•, HO_2_•, and NO concentrations. The details of the experiments have been described previously (*38*). We started our experiments by humidifying the air in the dark chamber to ~75% relative humidity and then the precursor α-pinene (~20 ppb) was added. In the low-NO experiment, no NO was added and the NO in the SAPHIR chamber arises from the photolysis of HONO produced via a well-characterized photolytic source related to the Teflon wall. The source strength is a function of relative humidity and the solar radiation (*52*). In the case of the high-NO experiment, additional NO (~20 ppb) was injected. Then, the shutter system of the roof was opened to start photooxidation of α-pinene using sunlight. In both low- and high-NO experiments, OH radicals were generated from the photolysis of HONO (*52*). No O_3_ was added in either of the experiments. During the early-stage reaction period (0 to 15 min), on which the focus is in this study, mixing ratios of O_3_ remained below the detection limit of 1 ppb (fig. S3) and photo-oxidation reactions were ensured in both low- and high-NO experiments.

A ^15^NO_3_^−^ chemical ionization mass spectrometer (Aerodyne Research Inc., USA) was used to detect gaseous HOM, particularly products with six or more oxygen atoms (*39*, *40*, *60*, *61*). Nitrogen-15 was used to distinguish reagent ion nitrogen from the nitrogen-14 atoms in the chamber-produced organic nitrates. The mass spectra within the mass range of *m/z* (mass/charge ratio) 4 to 1400 were analyzed using the Tofware analysis toolkit (Tofwerk/Aerodyne) in Igor Pro (WaveMetrics Inc.). High-resolution analysis was applied to identify ions with a resolving power *m/z*/Δ*m/z* of ~3000, with example fitting results shown in fig. S5. Other instruments used to characterize both gas- and particle-phase species are described in detail in section S2.

### C_10_H_15_O_X_•-related HOM molar yield quantification

According to the definition in Bianchi *et al.* (*18*), HOMs were identified as organic compounds containing six or more O atoms and formed from the autoxidation pathway. As C_10_H_15_O_X_•-related contributions to C_7_, C_10_, and C_20_ HOM could be identified, the contribution of C_10_H_15_O_X_•-related HOM to the total HOM concentration was estimated by summing over these contributions. Note that detailed calculations to separate the contribution of C_10_H_17_O_X_•-related carbonyl compounds to C_10_H_16_O_X_ can be found in section S3, with similar kinetics of C_10_H_15_O_X_• and C_10_H_17_O_X_• assumed. A calibration coefficient (*C*) was used to convert the mass spectra signals of HOM to concentrations. The calibration coefficient was derived from calibrations with H_2_SO_4_, whose charge efficiency is assumed to be the same as for HOM. The details of the calibration with H_2_SO_4_ can be found in Zhao *et al.* (*76*) and Shen *et al.* (*44*). A value of 2.5 × 10^10^ molecule cm^−3^ nc^−1^ (nc = normalized counts) with an uncertainty of −52%/+143% was used in this study. Note that accretion products are formed from two monomer peroxy radicals, so that molar concentrations of accretion products were multiplied by 2. The molar yield of total HOM was calculated as below

$$Y=\frac{\left[{\text{HOM}}_{C\leq 10}]+2\times [{\text{HOM}}_{C>10}\right]}{{\left[\alpha ‐\text{pinene}\right]}_{\text{reacted}}}=\frac{(I({\text{HOM}}_{C\leq 10})+2\times I({\text{HOM}}_{C>10}))\times C}{{\left[\alpha ‐\text{pinene}\right]}_{\text{reacted}}}$$ (1)

where [HOM] is the concentration of HOM, *I*(HOM) is peak intensity of HOM normalized to the total mass spectra signals, and [α-pinene]_reacted_ is the concentration of α-pinene reacted. The concentrations of HOM were also corrected for wall loss using a wall loss rate of 6 × 10^−4^ s^−1^ (low NO) (*77*) and 2.2 × 10^−3^ s^−1^ (high NO), and a dilution rate of 1.5 × 10^−5^ s^−1^ (both low and high NO). The higher wall loss rate at high NO is due to running fans in the chamber. The uncertainty of HOM molar yields is estimated to be −55%/+144%, with details shown in section S4.

### Theoretical kinetic predictions

The theoretical methodologies are described in detail in the Supplementary Materials. Briefly, quantum chemical calculations were performed at the CCSD(T)/aug-cc-pVTZ//M06-2X-D3/aug-cc-pVTZ level of theory for 30 reactions of RO_2_• radicals formed in the α-pinene H-abstraction channel. These data are used to derive rate coefficients using multiconformer transition state theory with Eckart tunneling correction, accounting for all conformers of reactants and transition states. For the reactions of noncyclic RO_2_• radicals and for alkoxy radicals in the oxidation scheme, no direct calculations were performed but rate coefficients were derived on the basis of SARs available in the literature; the procedure is described in detail in the Supplementary Materials.

## Supplementary Materials

### This PDF file includes:

Sections S1 to S12 Figs. S1 to S22 Tables S1 to S5 References
