# Supplementary material for: Unexpected significance of a minor reaction pathway in daytime formation of biogenic highly oxygenated organic compounds
Source: Sci Adv. 2022 Oct 21;8(42):eabp8702. doi: 10.1126/sciadv.abp8702 (PMC9586481; doi:10.1126/sciadv.abp8702)
Supplement: Supplementary file 1 — Sections S1 to S12 Figs. S1 to S22 Tables S1 to S5 References [file sciadv.abp8702_sm.pdf]

Science Advances
AAAS

Supplementary Materials for

Unexpected significance of a minor reaction pathway in daytime formation of biogenic highly oxygenated organic compounds

Hongru Shen *et al.*

Corresponding author: Defeng Zhao, dfzhao@fudan.edu.cn

Sci. Adv. **8**, eabp8702 (2022)
DOI: 10.1126/sciadv.abp8702

This PDF file includes:

Sections S1 to S12
Figs. S1 to S22
Tables S1 to S5
References

Section S1. General unimolecular and bimolecular reactions of RO_2•

Autoxidation has been recently identified as the key step leading to rapid formation of highly oxygenated organic molecules (HOM) (*11, 19*), via successive intramolecular hydrogen shift and O_2 addition, as shown in Fig. S1.

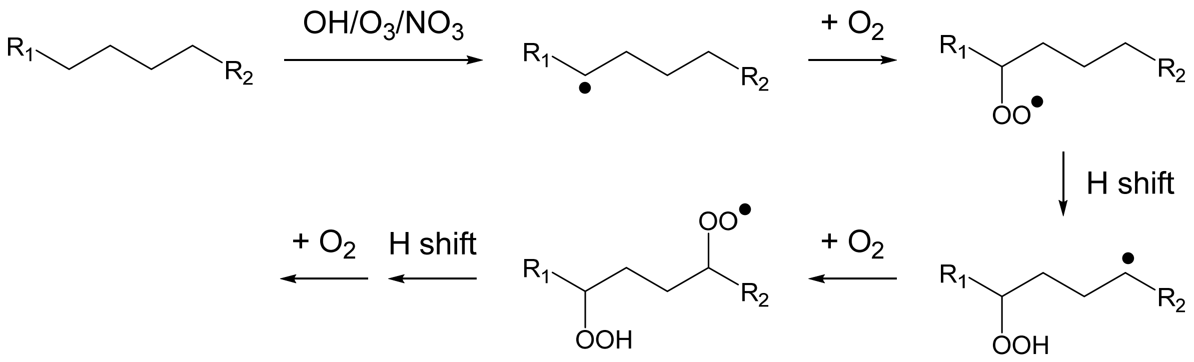

Fig. S1. Autoxidation steps. Successive intramolecular hydrogen shift and O_2-addition to form highly oxygenated organic molecules.

Both unimolecular and bimolecular reactions (*18, 37, 78*) of RO_2• can produce closed-shell products maintaining the C atom number, while gaining one more hydrogen atom, preserving hydrogen atom, or losing one hydrogen atom, as shown below (Fig. S2).

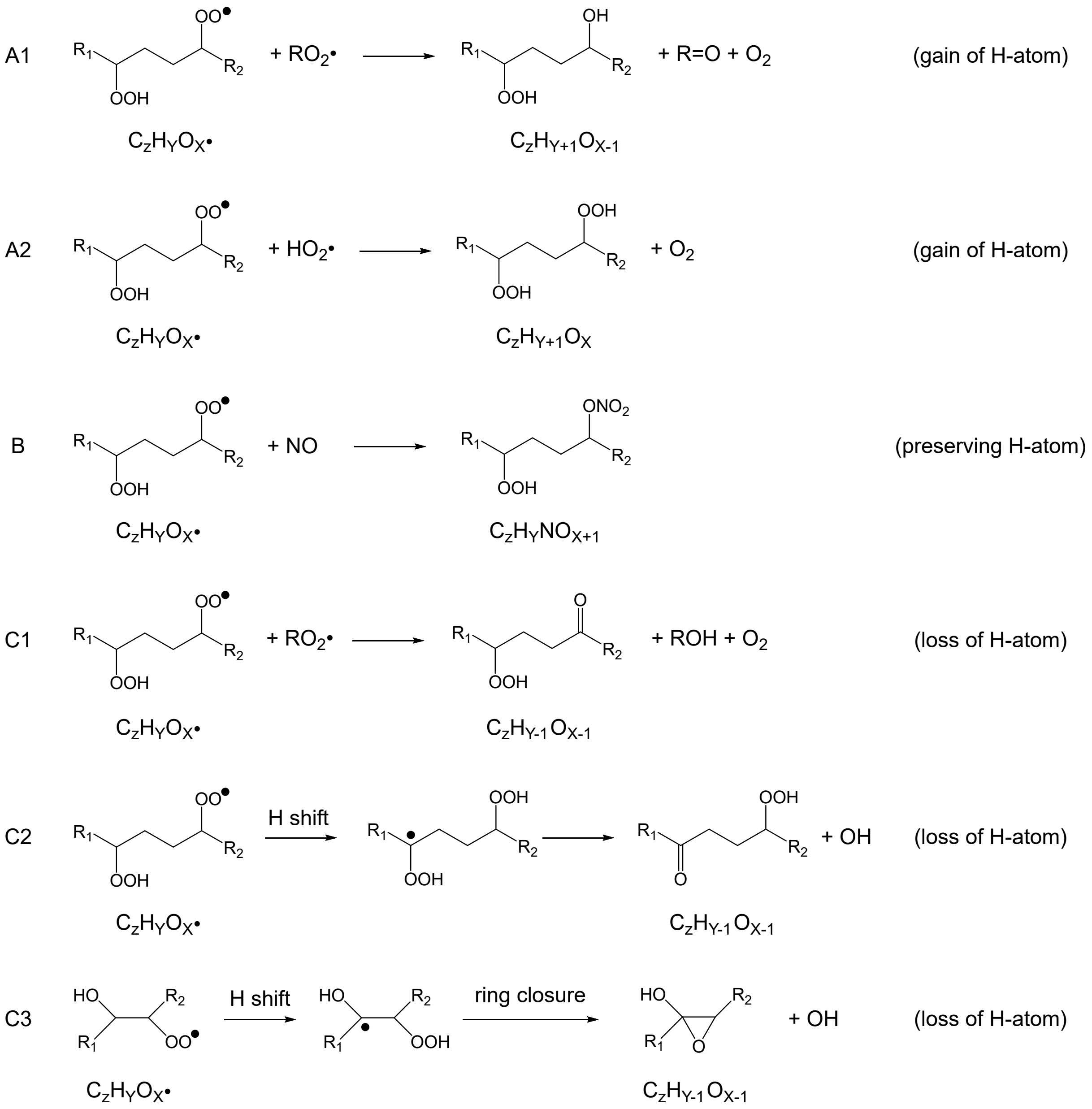

Fig. S2. Termination reactions of $C_ZH_YO_X$• peroxy radical to produce closed-shell products maintaining the number of C-atoms. Bimolecular reactions of individual peroxy radical $C_ZH_YO_X$• with (**A1**) RO_2• and (**A2**) HO_2• lead to closed-shell products gaining one more hydrogen atom as alcohols $C_ZH_{Y+1}O_{X-1}$ (ROH) or hydroperoxides $C_ZH_{Y+1}O_X$ (ROOH). Organic nitrates $C_ZH_YNO_{X+1}$ ($RONO_2$) are closed-shell products that preserve the number of hydrogen atoms, formed from (**B**) bimolecular reactions of $C_ZH_YO_X$• with NO. Closed-shell products losing one hydrogen atom are $C_ZH_{Y-1}O_{X-1}$, carbonyls (R=O) formed from (**C1**) bimolecular reactions of $C_ZH_YO_X$• with RO_2• and (**C2**) self-OH elimination of $C_ZH_YO_X$•, and epoxides formed from (**C3**) ring closure.

Section S2. Instrumentation & experimental overview

For gas-phase species, a set of instruments was applied, including a laser-induced fluorescence system (*79*) to measure concentrations of OH, HO_2•, and RO_2•, a proton-transfer-reaction time-of-flight mass spectrometer (PTR-ToF-MS, Ionicon Analytik, Austria) for concentrations of α-pinene, as well as a NO_X analyzer (Eco Physcis TR480) and an O_3 analyzer (Ansyco, model O341M). Their concentrations within the first 15 min reaction time are shown in Fig. S3. Note that concentrations of O_3 are under the detection limit (detection limit of 1 ppb and a precision of 0.5 ppb) in both low and high NO experiment and thus cannot be seen in the figure. This low O_3 concentrations also ensure that oxidation by OH radicals is overwhelming in our experiments. Further note that in the low NO experiment, the NO_X analyzer encountered a technical problem. The NO concentration based on the average concentration in the three other experiments at the same conditions were provided (solid black markers in Fig. S3A), which show good agreement with the NO concentrations (grey line) in Fig. S3A derived from the established methods in the SAPHIR chamber by Rohrer *et al.* (*52*). For all the low NO experiments in SAPHIR, a typical background concentration before roof opening is 10-30 ppt NO, which is also included in the Master Chemical Mechanism (MCM) HOM modeling for α-pinene + OH shown in this study. Measurements in the other low NO experiments (solid black markers) and simulated calculations (grey lines) in Fig. S3A showed that NO concentrations increased from 0-30 ppt to ~100 ppt during the first 15 min reaction time in this study.

For the particles, the number and size distribution were measured using a Scanning Mobility Particle Sizer (SMPS, TSI, DMA 3081/CPC 3785), with total number concentrations and surface area concentrations shown in Fig. S3. In both low and high NO experiments, negligible particles were formed during the first 15 min reaction time, with total number concentrations of <40 cm^{-3} at low NO and <20 cm^{-3} at high NO.

The physical parameters including temperature, pressure, relative humidity (RH), and solar irradiation (photolysis frequencies of J values such as J_{NO2}, J_{O1D}, J_{H2O2}, and J_{HONO}) were recorded throughout the chamber experimental runs.

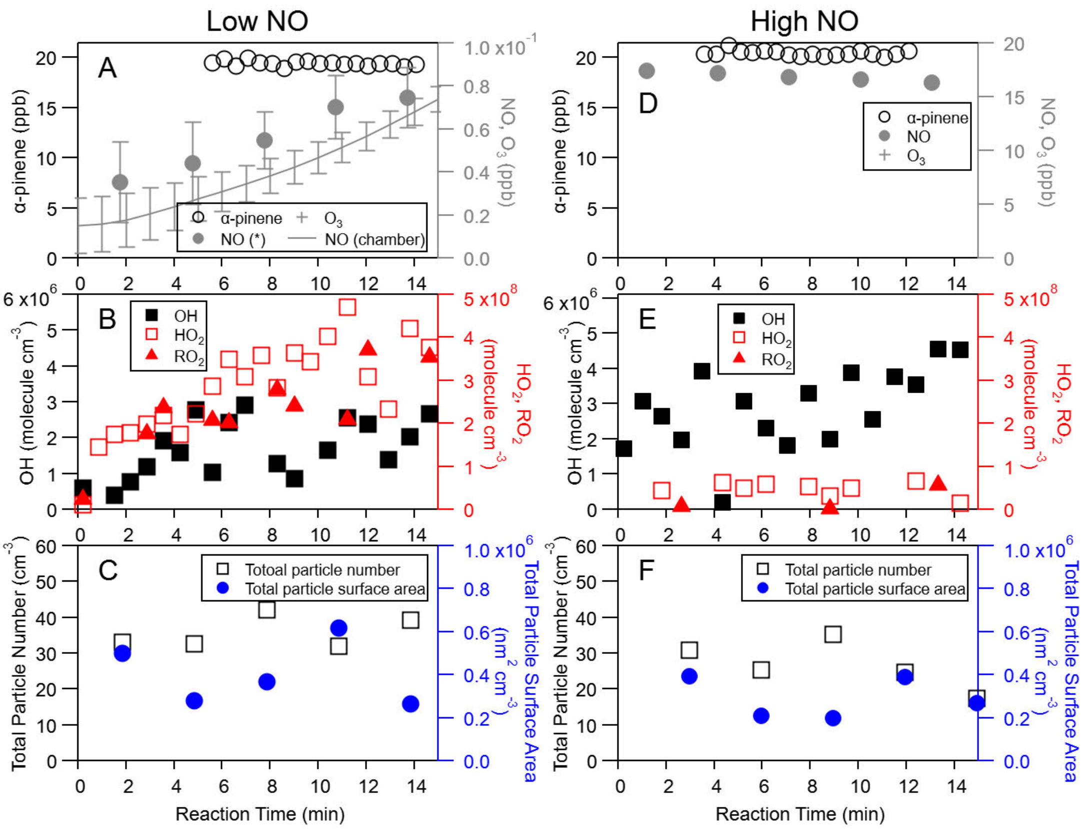

Fig. S3. Overview of low NO (30 - ~100 ppt) and high NO (~20 ppb) experimental conditions. Concentrations of gas phase species (α-pinene, NO, and O_3), radicals (OH, HO_2•, and RO_2•), and particles (total number concentrations and total surface area concentrations) within the first 15 min reaction time of (**A-C**) low NO and (**D-F)** high NO experiments. Concentrations of O_3 are under the detection limit (detection limit of 1 ppb and a precision of 0.5 ppb) in both low and high NO experiment and thus cannot be seen in the figure. NO(*) in (**A**) low NO experiment was the average NO concentration of the other three experiments performed at the same conditions, as no direct data was available due to a technical problem of the NO_X analyzer during this experiment. NO concentrations due to well-characterized emissions from the chamber wall of our SAPHIR chamber according to Rohrer *et al.* (*52*) are also shown (grey, denoted as NO (chamber)). All error bars in the figure represent one standard deviation.

Section S3. Calculations of carbonyls' contribution to $C_{10}H_{16}O_X$

According to the C_{10} closed-shell products from unimolecular and bimolecular reactions of the two C_{10}-HOM-RO_2• families ($C_{10}H_{15}O_X$• and $C_{10}H_{17}O_X$•, Fig. 1E in the main text and Fig. S2 in the Supplementary Materials), $C_{10}H_{16}O_X$ can be carbonyls (R=O) and epoxides from $C_{10}H_{17}O_X$• peroxy radicals, as well as alcohols (ROH) and hydroperoxides (ROOH) from $C_{10}H_{15}O_X$• peroxy radicals. Note that carbonyls and epoxides from $C_{10}H_{17}O_X$• share the same elemental composition and cannot be separated in this study. According to literature and quantum chemical calculations, carbonyls are likely to dominate over epoxides, which thus will not affect the calculations here. The quotient $\frac{d[ROH]/dt+d[ROOH]/dt}{d[R=O]/dt}$ was used to calculate the contribution of $C_{10}H_{17}O_X$•-related products to $C_{10}H_{16}O_X$, as shown below:

$$\frac{d[ROH]/dt+d[ROOH]/dt}{d[R_H=O]/dt}=\frac{\alpha k_{RO2+RO2}[RO_2][RO_2]^T+k_{RO2+HO2}[RO_2][HO_2]}{(1-\alpha)k_{RO2+RO2}[RO_2][RO_2]^T+k_{RO2}[RO_2]}=\frac{\alpha k_{RO2+RO2}[RO_2]^T+k_{RO2+HO2}[HO_2]}{(1-\alpha)k_{RO2+RO2}[RO_2]^T+k_{RO2}}$$
(Eq. S1)

where $[RO_2]^T$ is the total concentration of RO_2•, and [HO_2•] is the concentration of HO_2• in the reaction system. Herein $k_{RO2+RO2}$ and $k_{RO2+HO2}$ are the rate coefficients of the bimolecular reactions of RO_2• with RO_2• and HO_2•, and k_{RO2} is the rate coefficient of the unimolecular termination of RO_2•. In reactions of RO_2• + RO_2•, α represents the carbonyl yield and thus 1-α is the alcohol yield. We assume the same $k_{RO2+RO2}$, α, $k_{RO2+HO2}$, and k_{RO2} for a certain $C_{10}H_{15}O_X$•- and $C_{10}H_{17}O_X$• pair. As a consequence, the ratios of $\frac{d[ROH]+d[ROOH]}{d[R=O]}$ for $C_{10}H_{15}O_X$•-chemistry (Eq. S2) and $C_{10}H_{17}O_X$•-chemistry (Eq. S3) are the same. By combining Eq. S2 and S3 we obtain Eq. S4 which allows for calculating [R=O]%, the fractional contribution of the carbonyls produced from $C_{10}H_{17}O_X$• to $C_{10}H_{16}O_X$. Wall loss was neglected due to its minor effects on the concentrations of the products (<1%) and derived [R=O]% values (<3%) during the first 15 min reaction time in this study.

$$\left.\frac{d[ROH]/dt+d[ROOH]/dt}{d[R=O]/dt}\right|_{C_{10}H_{17}O_x\bullet}=\frac{d[C_{10}H_{18}O_x]}{d[C_{10}H_{16}O_x]_{R=O}}=\frac{d[C_{10}H_{18}O_x]}{d[C_{10}H_{16}O_x]\times[R=O]\%} \quad \text{(Eq. S2)}$$

$$\left.\frac{d[ROH]/dt+d[ROOH]/dt}{d[R=O]/dt}\right|_{C_{10}H_{15}O_x\bullet}=\frac{d[C_{10}H_{16}O_x]_{ROH+ROOH}}{d[C_{10}H_{14}O_x]}=\frac{d[C_{10}H_{16}O_x]\times(1-[R=O]\%)}{d[C_{10}H_{14}O_x]} \quad \text{(Eq. S3)}$$

$$\frac{d[C_{10}H_{16}O_x]\times(1-[R=O]\%)}{d[C_{10}H_{14}O_x]}=\frac{d[C_{10}H_{18}O_x]}{d[C_{10}H_{16}O_x]\times[R=O]\%} \quad \text{(Eq. S4)}$$

About 97% and 78% of $C_{10}H_{16}O_X$ were estimated to be carbonyls from $C_{10}H_{17}O_X$• at low and high NO, respectively. Similar concentration ratios of carbonyl families ($C_{10}H_{14}O_X$/$C_{10}H_{16}O_X$) and organic nitrate families ($C_{10}H_{15}NO_X$/$C_{10}H_{17}NO_X$) are obtained, as shown in Fig. 1C, 1D in the

main text. In C_7-HOM, as discussed in Section S5 in the Supplementary Materials, $C_7H_{10}O_X$ can be carbonyls formed from $C_7H_{11}O_X$•, as well as alcohols and hydroperoxides from $C_7H_9O_X$•. Same concentration ratios of carbonyls ($C_7H_8O_X$/$C_7H_{10}O_X$) to that of organic nitrates ($C_7H_9NO_X$/$C_7H_{11}NO_X$) were used to separate carbonyls' contribution to $C_7H_{10}O_X$.

Section S4. Uncertainties of HOM yield

The direct calibration method using HOM standards is not possible so far, mainly due to the difficulty to synthesize pure HOM and unknown chemical structures of many HOM. However, as discussed by Ehn *et al.* (*11*), the sensitivity of NO_3^--CIMS to H_2SO_4 is similar to HOM, and thus the calibration coefficient of H_2SO_4 is commonly applied to HOM (*11, 12, 21, 78*). Effective deprotonation of H_2SO_4 leads to formation of clusters with NO_3^- ions, with rates close to the kinetic collision limit. In this study, most of detected HOM are binding with NO_3^- reagent ions, also near the collision limit as H_2SO_4. Combining the theoretical calculations (*39, 40*) and our previous experimental results (*41*) discussed here and in the main text, we conclude that the sensitivity of NO_3^--CIMS to all HOM is similar to that of H_2SO_4 in this study and the calibration coefficient of H_2SO_4 can be applied to all HOM. If the HOM charge at rates lower than the collision limit, the calibration coefficient would be overestimated and the HOM concentrations derived using the calibration factor of H_2SO_4 thus constitute a lower limit with an uncertainty by a factor of 2 (*58*).

The peak intensities of H_2SO_4 can be converted to concentrations following Eq. S5.

$$[H_2SO_4] = C \times I \qquad \text{(Eq. S5)}$$

where $[H_2SO_4]$ is the concentration of H_2SO_4 (molecule cm^{-3}), C is the calibration coefficient, and I is peak intensities in the unit of normalized count (nc), *i.e.* the peak area of H_2SO_4 divided by total signals. The detailed method deriving the calibration coefficient of H_2SO_4 can be found in Zhao *et al.* (*76*). Briefly, oxidation of SO_2 by OH was performed in the SAPHIR chamber to produce *in-situ* H_2SO_4, by injecting SO_2 (~15 ppb) in the chamber and opening the roof to produce OH radicals from the photolysis of HONO. Additional NO (~20 ppb) was added to enhance OH production. Following the reaction kinetics, concentrations of H_2SO_4 can be described as:

$$\frac{d[H_2SO_4]}{dt} = k \times [SO_2] \times [OH] - k_{wall\ loss} \times [H_2SO_4] - k_{dilution\ loss} \times [H_2SO_4] \qquad \text{(Eq. S6)}$$

where $[H_2SO_4]$, $[SO_2]$, and [OH] are the concentrations of H_2SO_4, SO_2, and OH (molecule cm^{-3}), respectively, k is the rate coefficient (1.1×10^{-12} cm^3 $molecule^{-1}$ s^{-1}, Atkinson *et al.* (*80*)) of the OH oxidation reaction of SO_2, and $k_{wall\ loss}$ and $k_{dilution\ loss}$ are the wall loss rate (~6.0×10^{-4} s^{-1}, Zhao *et al.* (*38*)) and dilution loss rate (~1×10^{-5} s^{-1}) of H_2SO_4, respectively. Combining Eq. S5 and Eq. S6, C can be obtained as:

$$C = \frac{k \times [SO_2] \times [OH]}{\frac{I - I_0}{t} + (k_{wall\ loss} + k_{dilution\ loss}) \times I} \qquad \text{(Eq. S7)}$$

where I_0 is the peak intensity at the reaction time zero. Based on the uncertainties on SO_2 concentration (~7%), OH concentration (~10%), I (~10%), k (Δlogk=±0.3), transmission efficiency (-0%/+14%), and sensitivity (-0%/+100%), the uncertainty of C was estimated to be -52%/+143%. Integrating the uncertainties of HOM peak intensity (~10%) and the α-pinene concentration (~15%), an uncertainty of HOM yield is estimated to be -55%/+144%, following equation 1 in the main text.

Moreover, HOM concentrations were corrected for wall loss and dilution. The value of 6×10^{-4} s^{-1} and 2.2×10^{-3} s^{-1} was respectively used for the wall loss correction $k_{wall\ loss}$ at low and high NO based on our previous study (*38*) and our recent updates for different conditions. Acting mixing due to a running fan in our high NO experiments leads to a larger wall loss rate. Although wall loss rates of less oxygenated compounds can change during an experiment (*81*), in this study, we focus on HOM and short reaction times (0-15 min), therefore HOM yields are not expected to be sensitive to the wall loss rate. Sensitivity analyses showed that variation of the wall loss rate from +100% to -50% led to changes in the molar HOM yield between +28.2% and -13.9% in the low NO experiment and between +76.0% and -36.2% in the high NO experiment. The dilution rate $k_{dilution\ loss}$ was derived from a constant dilution flow rate of the chamber, leading to a value of 1.5×10^{-5} s^{-1}.

Section S5. C_{10} HOM distribution

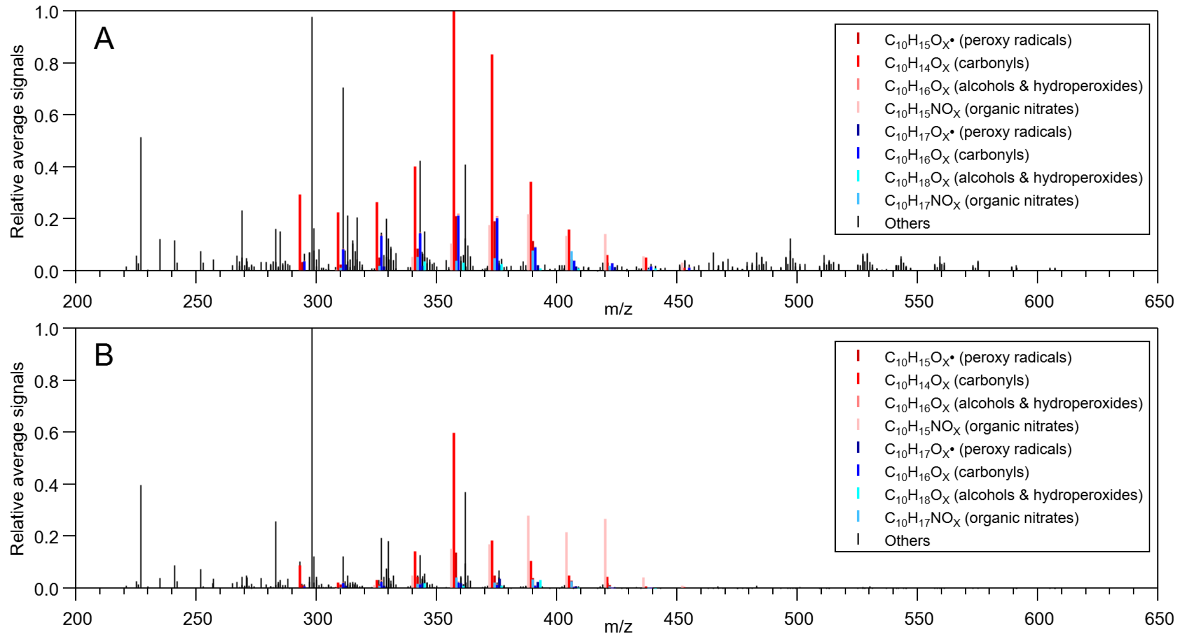

Fig. S4. Average mass spectrum of identified HOM from OH oxidation of α-pinene during the first 15 min reaction time at (**A**) low NO (30 - ~100 ppt) and (**B**) high NO (~20 ppb), with a resolving power *m/z/Δm/z* of ~3000. Identified HOM are detected as clusters with reagent ion $^{15}NO_3^-$. Note that background signals (-10 - 0 min reaction time) were subtracted.

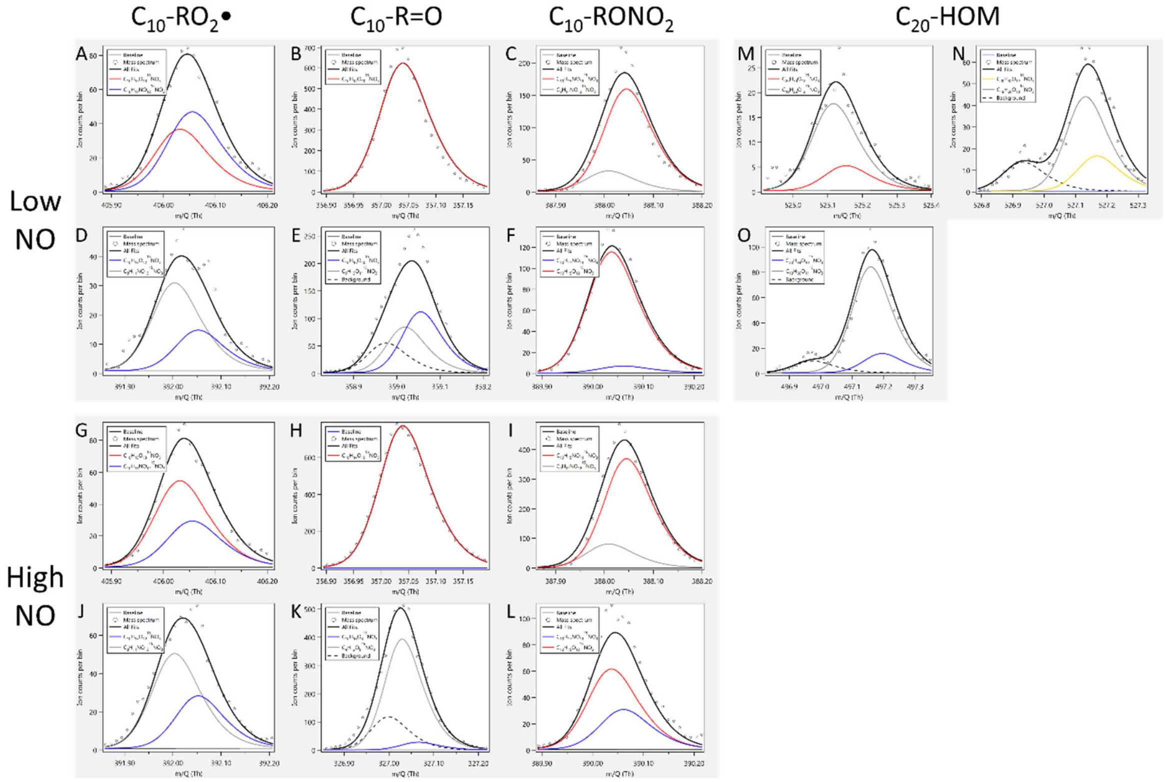

Fig. S5. Examples of high-resolution peak fittings of $C_{10}H_{15}O_X$• and $C_{10}H_{17}O_X$• related C_{10}- and C_{20}-HOM. Assignment of representative peroxy radicals, carbonyls, organic nitrates, and accretion products of $C_{10}H_{15}O_X$• (red) and $C_{10}H_{17}O_X$• (blue) related C_{10}- and C_{20}-HOM in high resolution spectra at (**A-F** and **M-O**) low NO (30 - ~100 ppt) and (**G-L**) high NO (~20 ppb).

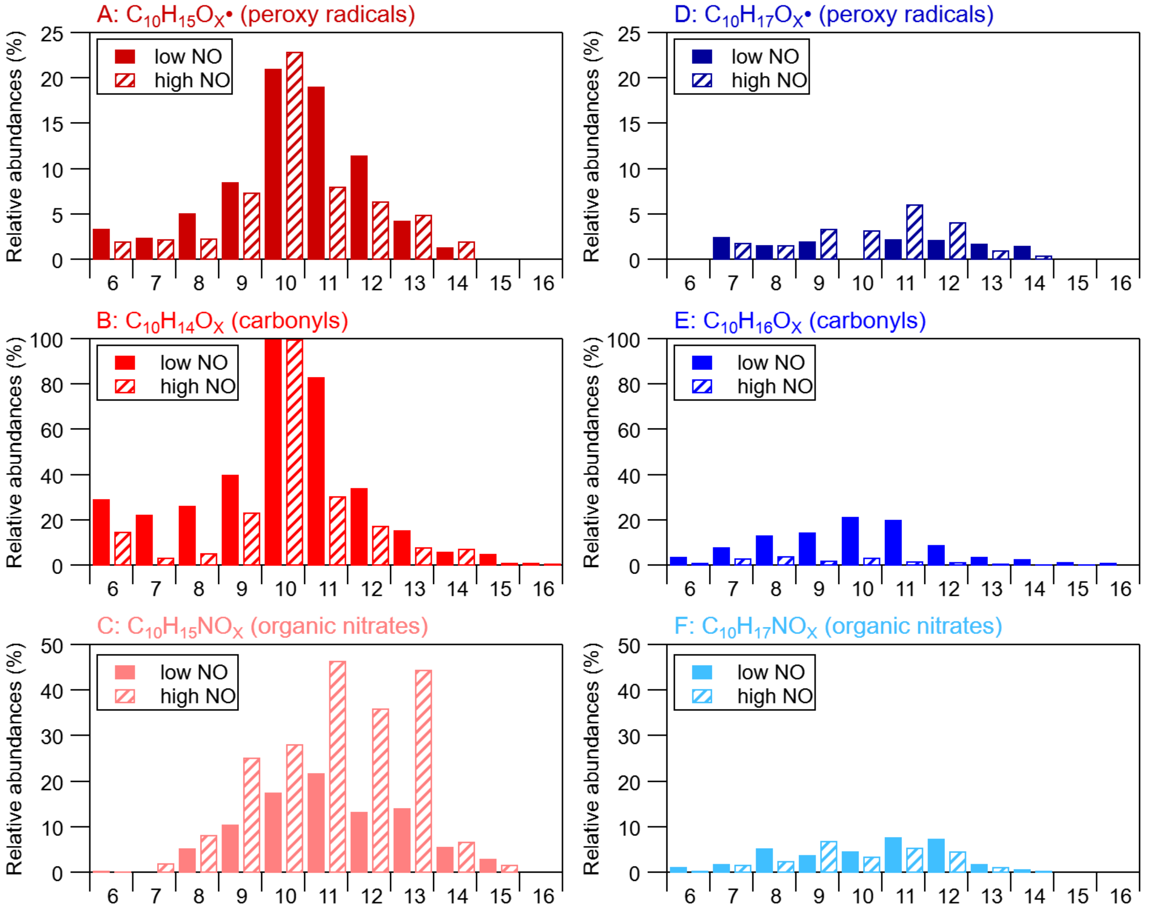

Fig. S6. Relative abundances of individual products during the first 15 min reaction time at low NO (30 - ~100 ppt, solid bars) and high NO (~20 ppb, patterned bars). Left-hand side: (**A**) $C_{10}H_{15}O_X$• (peroxy radicals, X=6-14) and its related products of (**B**) $C_{10}H_{14}O_X$ (carbonyls, X=6-16) and (**C**) $C_{10}H_{15}NO_X$ (organic nitrates, X=6-15). Right-hand side: (**D**) $C_{10}H_{17}O_X$• (peroxy radicals, X=7-14) and its related products of (**E**) $C_{10}H_{16}O_X$ (carbonyls, X=6-16) and (**F**) $C_{10}H_{17}NO_X$ (organic nitrates, X=6-14). $C_{10}H_{15}O_X$•-related C_{10}-HOM are denoted in red while $C_{10}H_{15}O_X$•-related C_{10}-HOM are in blue. All products are normalized to the signals of $C_{10}H_{14}O_{10}$, the most abundant individual product at both low and high NO. Carbonyls $C_{10}H_{16}O_X$ were estimated following Section S3.

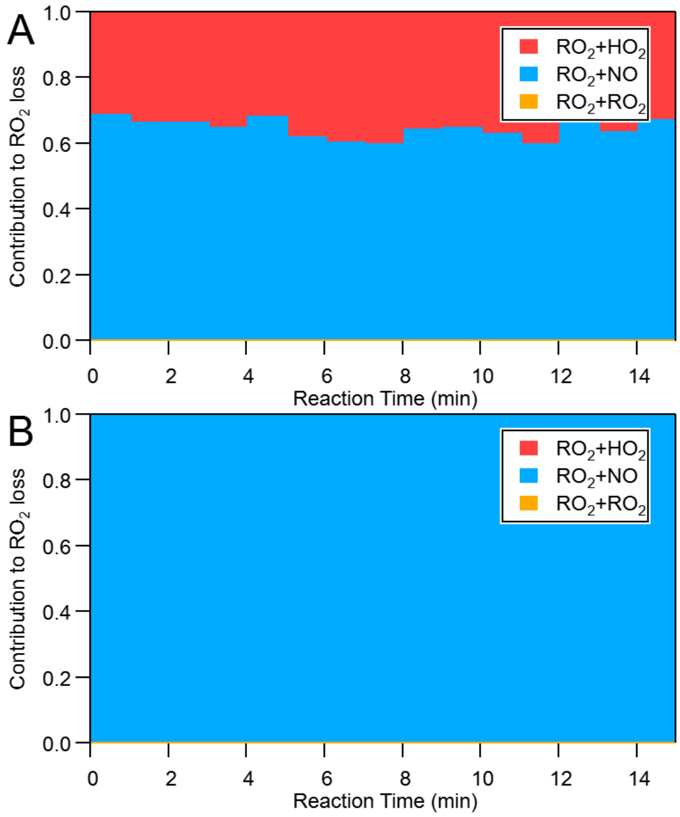

Fig. S7. Bimolecular loss fate of peroxy radicals. Contribution of RO_2• loss via the reaction with HO_2 (red), NO (blue), and RO_2• (yellow) to total RO_2• loss during the first 15 min reaction time at (**A**) low NO (30 - ~100 ppt) and (**B**) high NO (~20 ppb), based on measured data. The loss rate of RO_2•+ RO_2• was estimated using a reaction coefficient of 2.5×10^{-13} $molecule^{-1}$ cm^3 s^{-1} (*82*).

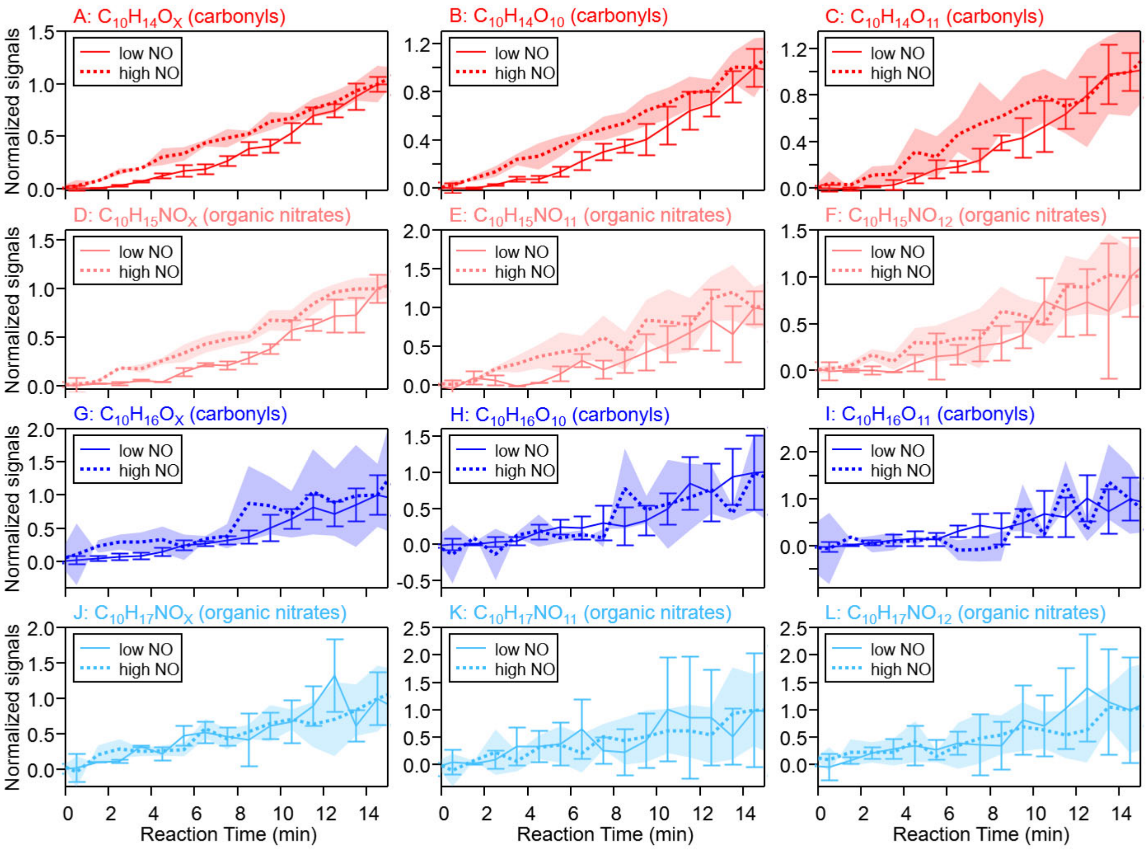

Fig. S8. Time series of lumped product families and representative individual products. Normalized signals of $C_{10}H_{15}O_X$• related C_{10}-HOM (in red colors), including (**A-C**) $C_{10}H_{14}O_X$ family, $C_{10}H_{14}O_{10}$, $C_{10}H_{14}O_{11}$, and (**D-F**) $C_{10}H_{15}NO_X$ family, $C_{10}H_{15}NO_{11}$, $C_{10}H_{15}NO_{12}$, and $C_{10}H_{17}O_X$• related C_{10}-HOM (in blue colors), including (**G-I**) $C_{10}H_{16}O_X$ family, $C_{10}H_{16}O_{10}$, $C_{10}H_{16}O_{11}$, and (**J-L**) $C_{10}H_{17}NO_X$ family, $C_{10}H_{17}NO_{11}$, $C_{10}H_{17}NO_{12}$, as a function of reaction time during the first 15 min reaction time at low NO (30 - ~100 ppt, solid lines) and high NO (~20 ppb, dashed lines). Note that the error bars (low NO) and error shades (high NO) represent one standard deviations.

Section S6. C_7 HOM

Besides C_{10}-HOM, both $C_{<10}$-HOM and $C_{>10}$-HOM were observed, as shown Fig. S9. As discussed in the main text, $C_{>10}$-HOM observations (with C_{20}-HOM as an example) also indicated a chemistry dominated by $C_{10}H_{15}O_x$•. For $C_{<10}$-HOM, C_7-HOM are the most abundant products in both low and high NO cases. Their detailed formation chemistry of C_7-HOM and their relation to C_{10}-HOM is discussed below.

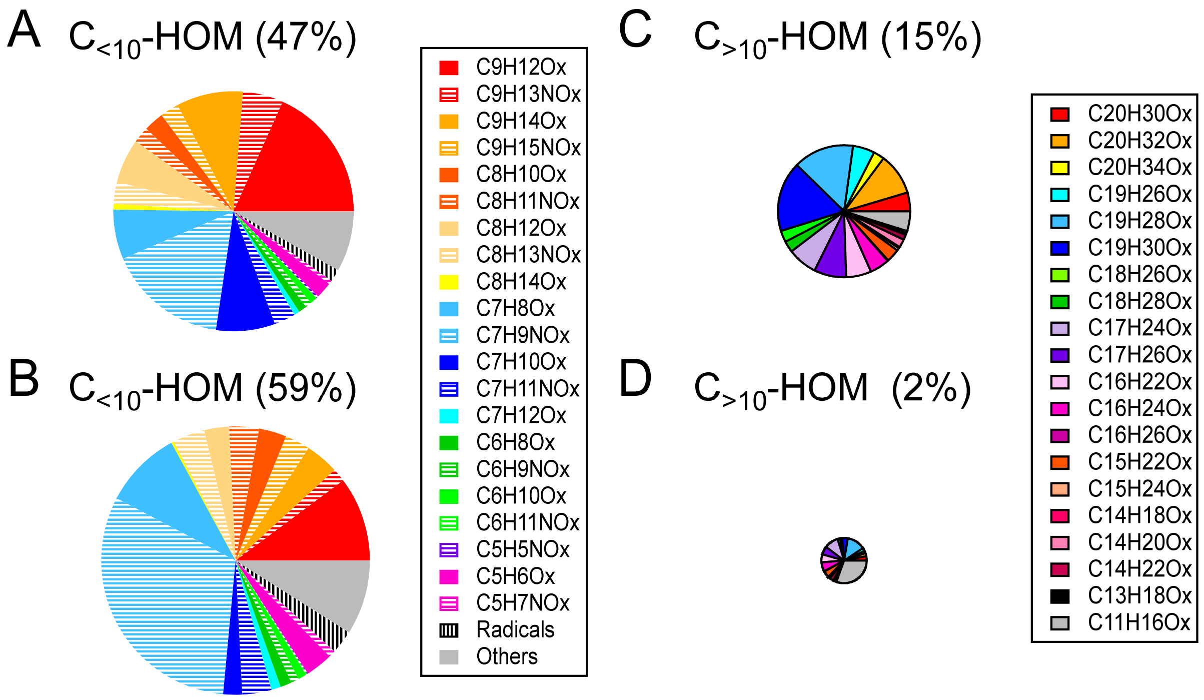

Fig. S9. Relative abundance of other HOM. Relative abundances of $C_{<10}$-HOM at (**A**) low NO (30 - ~100 ppt) and (**B**) high NO (~20 ppb), with carbonyls/alcohols/hydroperoxides in solid, organic nitrates in horizontal stripes, and peroxy radicals in vertical stripes, and $C_{>10}$-HOM (in black boxes) at (**C**) low and (**D**) high NO. Their contributions to all observed HOM in low and high NO experiments are denoted in parenthesis. The area of the pie charts are sized proportionally to the relative contributions to the total HOM.

Two families of peroxy radicals, $C_7H_9O_x$• and $C_7H_{11}O_x$•, are identified in C_7-HOM (Fig. S9), together with their corresponding C_7 closed-shell products, carbonyls ($C_7H_8O_x$ and $C_7H_{10}O_x$) and organic nitrates ($C_7H_9NO_x$ and $C_7H_{11}NO_x$). Higher concentrations of $C_7H_9O_x$•-related C_7 closed-shell products were observed compared to $C_7H_{11}O_x$•, with concentration ratios of carbonyls ($C_7H_8O_x/C_7H_{10}O_x$) of 9.8 and 7.1 at low and high NO, respectively, and concentration ratios of organic nitrates ($C_7H_9NO_x/C_7H_{11}NO_x$) of 5.7 and 8.8 at low and high NO. According to the current master chemical mechanism MCM v.3.3.1 (*48, 83*) and calculations by Vereecken and Peeters (*29*), $C_7H_{11}O_x$• are expected to be formed from the decomposition of $C_{10}H_{17}O_x$• with acetone as a byproduct as shown in Fig. S10A. $C_7H_9O_x$• are proposed to be produced from decomposition of $C_{10}H_{15}O_x$•. As shown in Fig. S10, one alkoxy step for R6OO, R9OO, and R10OO (Fig. 2 in the main text) leads to alkoxy radicals R6O, R9O, and R10O, whose subsequent decomposition reaction is expected to form alkyl radicals $C_7H_9O_{2,4}$• and acetone. The following autoxidation of successive O_2-addition and H-shift is proposed to form highly oxygenated $C_7H_9O_x$•. Thereby, the C_7-HOM product distribution is consistent with C_{10}-HOM, further confirming the importance of $C_{10}H_{15}O_x$• in α-pinene + OH.

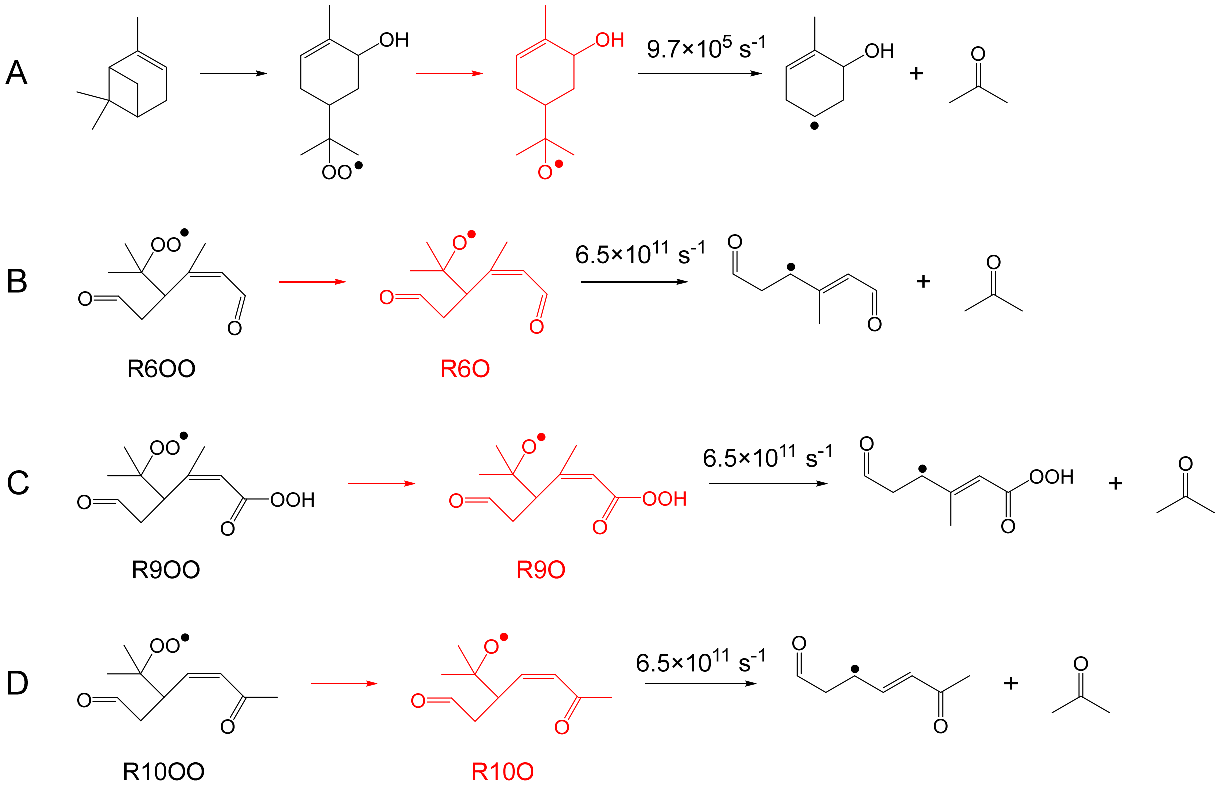

Fig. S10. Example formation pathway of C_7 products. Example formation pathway of (**A**) $C_7H_{11}O$• alkyl radicals from $C_{10}H_{17}O_3$• peroxy radicals and (**B, C, D**) $C_7H_9O_{2,4}$• alkyl radicals from $C_{10}H_{15}O_{4,6}$• peroxy radicals; decomposition rates calculated as described in Vereecken and Peeters (*51*). The formation pathway of R6OO, R9OO, and R10OO are shown in Fig. 2 in the main text. Alkoxy steps are marked in red. Note that alkoxy steps in **A-D** are more likely to occur at high NO

while unimolecular reactions are more likely at low NO (Fig. S12A for $C_{10}H_{17}O_3$•; Fig. 2 for R6OO, R9OO, and R10OO).

Moreover, higher concentrations of C_7-HOM at high NO (Fig. S9B) than those at low NO (Fig. S9A) are also consistent with more active alkoxy steps at high NO than at low NO, where unimolecular reactions of C_{10}-RO_2• are more competitive due to the longer lifetime at low NO. Overall, the distributions of C_7-HOM at both low and high NO are consistent with the important role of alkoxy radicals in HOM formation, particularly for $C_{10}H_{15}O_X$• and its related HOM in this study.

Similar to the C_{10}-HOM case, more $C_7H_9O_X$•-related HOM (carbonyls $C_7H_8O_X$ and organic nitrates $C_7H_9NO_X$) are formed at earlier reaction time in high NO experiments compared to low NO experiments, as shown in Fig. S11. As proposed, $C_7H_9O_X$• and $C_7H_{11}O_X$• are respectively formed from $C_{10}H_{15}O_X$• and $C_{10}H_{17}O_X$•. As discussed in the main text, the formation rate of $C_{10}H_{15}O_X$• depends on NO concentrations, which does not influence the formation of $C_{10}H_{17}O_X$•. Concentration ratios of their related C_7 closed-shell products ($C_7H_8O_X$/$C_7H_{10}O_X$ and $C_7H_9NO_X$/$C_7H_{11}NO_X$) reached values of 1, where $C_7H_9O_X$• chemistry is considered to be equivalent to $C_7H_{11}O_X$, at a reaction time of >5 min at low NO and 1-2 min at high NO. This faster appearance of $C_7H_9O_X$•-dominated chemistry at high NO again agrees with the temporal behavior of C_{10} HOM (Fig. 3 in the main text) and provides additional evidence for the importance of alkoxy steps in this study. Additionally, the formation of C_7 HOM is an evident support for the existence of alkoxy steps in α-pinene + OH.

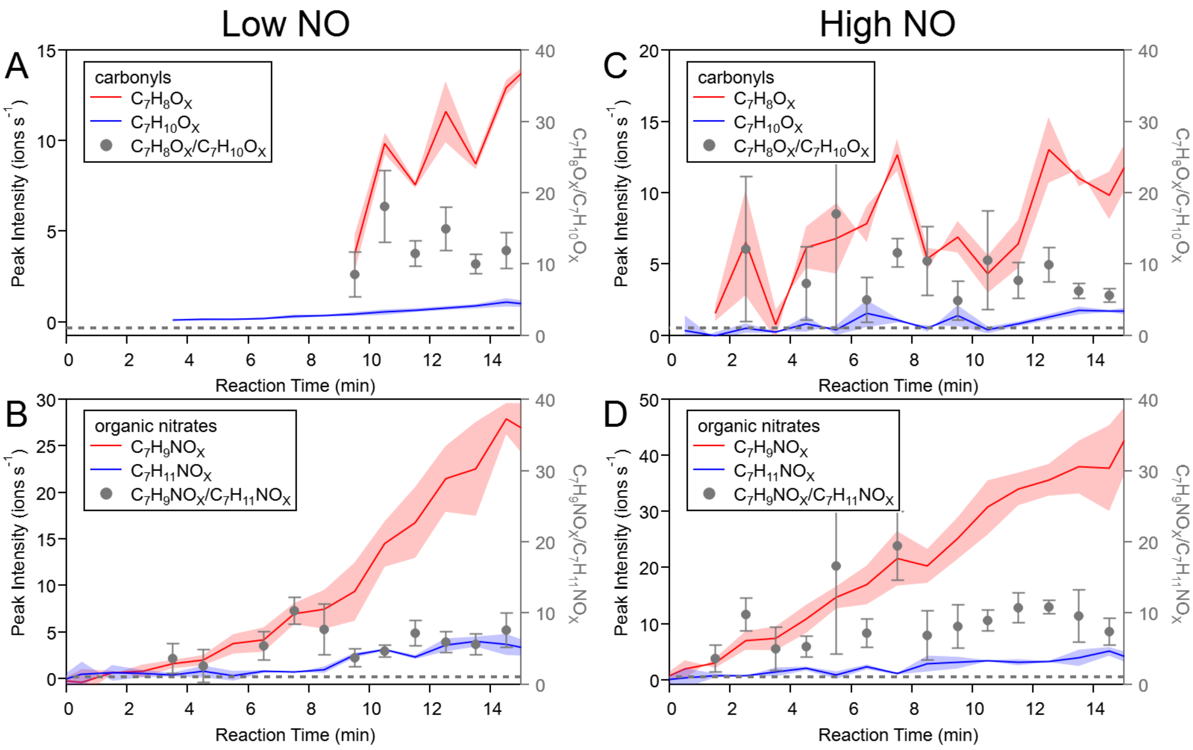

Fig. S11. Time series of C_7 carbonyls and organic nitrates in low NO (30 - ~100 ppt) and high NO (~20 ppb) experiments. The carbonyl family $C_7H_8O_X$ (red), $C_7H_{10}O_X$ (blue), and their concentration ratio ($C_7H_8O_X/C_7H_{10}O_X$, grey dots) during the first 15 min reaction time are shown in (**A**) low and (**C**) high NO experiments. Time series of the organic nitrate family, including $C_7H_9NO_X$ (red), $C_7H_{11}NO_X$ (blue), and their concentration ratio ($C_7H_9NO_X/C_7H_{11}NO_X$, grey dots), are similarly demonstrated in (**B**) low and (**D**) high NO experiments. The horizontal dashed lines represent a concentration ratio of 1, where $C_7H_9O_X$• chemistry is equivalent to $C_7H_{11}O_X$• chemistry. All data are averaged to a time resolution of 1 min. In each panel, error bars of C_7 carbonyls and organic nitrates represent one standard deviation (left axes, peak intensities lower than background signals are not shown) and error bars of concentration ratios (right axes, uncertainties more than 200% are not shown) were calculated using error propagation.

Section S7. Pinonaldehyde production from OH addition channel

For the established OH addition channel, direct autoxidation is expected to occur in channel A in Fig. S12, leading to $C_{10}H_{17}O_X$• (*25-27*). The branching ratio for this ring-opened RO_2• ranges from 7.5% to 22% (MCM v3.3.1 and Peeters *et al.* (*28*), respectively). This limited reaction contribution can be one possible reason for the low concentrations of $C_{10}H_{17}O_X$• related HOM observed in this study. Channels B and C lead to a major product pinonaldehyde $C_{10}H_{16}O_2$ (*84*), whose aldehydic hydrogen atom can be readily abstracted by OH radical again, leading to the indirect formation of $C_{10}H_{15}O_X$• via following autoxidation steps (Fig. S12B, S12C). The limited role of hydrogen abstraction from pinonaldehyde for production of $C_{10}H_{15}O_X$• related HOM in this study will be discussed in the following.

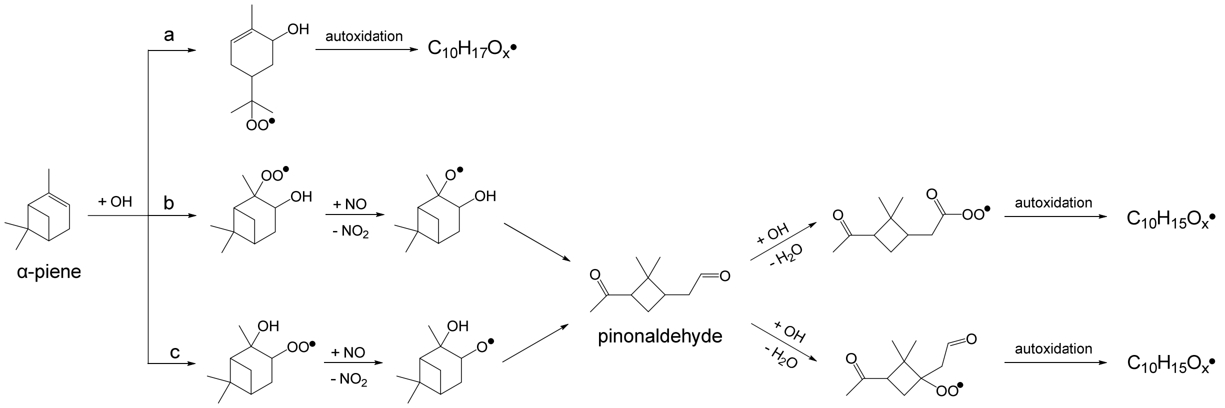

Fig. S12. Simplified mechanism of OH addition channel of α-pinene. The formation of (**A**) $C_{10}H_{17}O_X$• from α-pinene + OH via the addition channel according to the established literature (*25-27*). The formation of (**B, C**) $C_{10}H_{15}O_X$• from hydrogen abstraction from pinonaldehyde, the main product after OH addition to α-pinene. Note that autoxidation pathways in channel (**A**) likely play a more important role at low NO than alkoxy steps (Fig. S10A) which is expected to be more important at high NO.

Compared to the direct hydrogen abstraction from α-pinene, the hydrogen abstraction from pinonaldehyde pathway is expected be occur later since pinonaldehyde must first be formed via OH addition to α-pinene (*85*). Considering the rapid formation of $C_{10}H_{15}O_X$• and related HOM (within 3 min) observed in this study, first-generation peroxy radicals are likely to dominate in $C_{10}H_{15}O_X$• formation compared to any second generation peroxy radicals from pinonaldehyde. In order to quantify the relative importance of the two pathways, we calculated the relative reaction rate (Equation S8) of hydrogen abstraction from α-pinene to that of pinonaldehyde by OH radicals, as shown below:

$$\frac{R[AP+OH]_{H\ abstraction}}{R[PINAL+OH]} = \frac{k[AP+OH]\times[AP]\times[OH]\times BR[H\ \ abstraction]}{k[PINAL+OH]\times[PINAL]\times[OH]} = \frac{k[AP+OH]\times[AP]\times BR[H\ \ abstraction]}{k[PINAL+OH]\times[AP]_r\times Y[PINAL]}$$

(Eq. S8)

where k[AP+OH] and k[PINAL+OH] are temperature-dependent reaction rate constants taken from MCM v3.3.1 (*48, 83*). Here, BR[H-abstraction] is the branching ratio for α-pinene + OH reactions to undergo H-abstraction channel. As the H-abstraction channel is not included in current MCM v3.3.1, we added it with branching ratio of 0.09 (*28*). Concentrations of α-pinene were measured in the experiments while concentrations of pinonaldehyde were estimated according to their NO-dependent yields (Y[PINAL] in Equation S8), with a range of 0.05-0.13 at low NO and 0.3-0.45 at high NO (*85-87*). In this study, 0.05 and 0.3 were used for the yield Y[PINAL] in low NO and high NO experiments, respectively. The uncertainties of $\frac{R[AP+OH]_{H\ abstraction}}{R[PINAL+OH]}$ was estimated to be -40%/+168% at low NO and -40%/+71% at high NO, from the uncertainty of α-pinene concentration (~15%), k[AP+OH] (Δlogk=±0.08), k[PINAL+OH] (Δlogk=±0.15), and Y[PINAL] (-0%/+160% at low NO and -0%/+50% at high NO) using error propagation.

The relative reaction rate $\frac{R[AP+OH]_{H\ abstraction}}{R[PINAL+OH]}$ along with uncertainties during the first 15 min reaction are shown in Fig. S13. Hydrogen abstraction from α-pinene is more than 70 times faster than that from pinonaldehyde at low NO and more than 8 times at high NO. Note that the concentrations of pinonaldehyde were estimated from consumed α-pinene and yields of pinonaldehyde, thus reflecting only the production. As pinonaldehyde is continuously consumed by OH, its true concentration should be lower, and thus its relative importance is even overestimated using this method. Overall, we thus conclude that pinonaldehyde has only a minor to negligible contribution to HOM formation at early stages of the experiments.

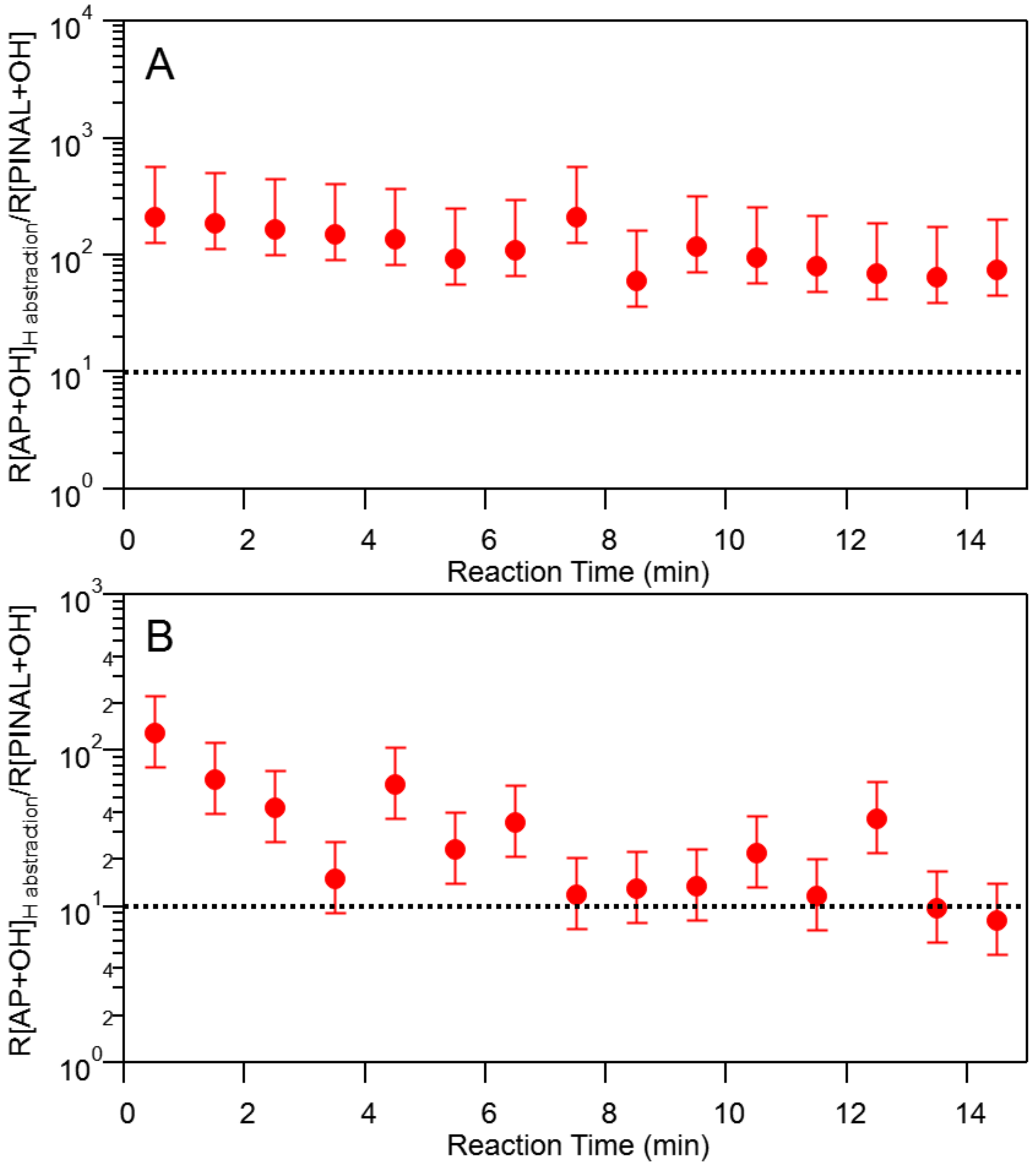

Fig. S13. Relative reaction rate of hydrogen abstraction by OH from α-pinene to that from pinonaldehyde. Data within the first 15 min reaction time of (**A**) low NO (30 - ~100 ppt) and (**B**) high NO (~20 ppb) experiments, with dashed lines as the value of 10. Note that the y axes in panel (**A**) and (**B**) are different, but both on log scale.

Section S8. Theoretical methodology

The geometries of the intermediates and transition states in the mechanism were first optimized using the M06-2X/cc-pVDZ methodology (*88, 89*), with an exhaustive characterization of all conformers for each reactant and transition state. All geometries obtained thus were further optimized at the M06-2X-D3/aug-cc-pVTZ level of theory which includes Grimme *et al.* (*90*). D3 diffusion corrections (*90, 91*). Moments of inertia for molecular rotation, and wavenumbers for vibration were obtained at the same level of theory, with a vibrational scaling factor of 0.971 (*89, 92*). The transition states were validated by IRC calculations and by visual verification of the imaginary frequency vibrational mode. Finally, the relative energies were further improved by single-point calculations at the CCSD(T)/aug-cc-pVTZ level of theory (*93, 94*); this final level of theory is referred to as CCSD(T)//M06-2X-D3. T1 diagnostics at that level of theory did not reveal high multi-reference character for any of the structures ($T1 \leq 0.028$). The expected uncertainty on the reaction barrier heights at this level of theory is ±0.5 kcal mol^{-1}. All quantum chemical calculations were performed using the Gaussian-16 software suite (*95*). The quantum chemical data underlying the theoretical kinetic calculations can be found as a text file at https://doi.org/10.26165/JUELICH-DATA/GLU9BW.

The rate coefficients for the individual reactions at the high-pressure limit were then calculated using multi-conformer transition state theory, MC-TST (*96*), incorporating the data for all conformers obtained as described above. Tunneling is accounted for using an asymmetric Eckart barrier correction (*97, 98*). Based on earlier work at a similar level of theory in comparison with experimental data on H-migration in RO_2• radicals with no or only one oxygenated functionality (*50, 99*) and available theoretical literature data on ring closure reactions (*56*), we estimate the thermal rates to be accurate to a factor 2 to 3.

Section S9. Theoretical kinetic study of RO_2• radicals after H-abstraction

As already studied in prior literature (*28, 29, 33*), the dominant H-abstraction sites in α-pinene are those that lead to allyl-resonance stabilized alkyl radicals. Under atmospheric conditions, these allyl radicals will undergo O_2 addition, which can occur on either side of the ring for endocyclic radical sites. Formally, there are then 7 distinct RO_2• radicals that need to be examined (see Fig. S14). Of these, R2OO is expected to be dominant under atmospheric conditions, with an estimated 40:60 *syn* to *anti* ratio.

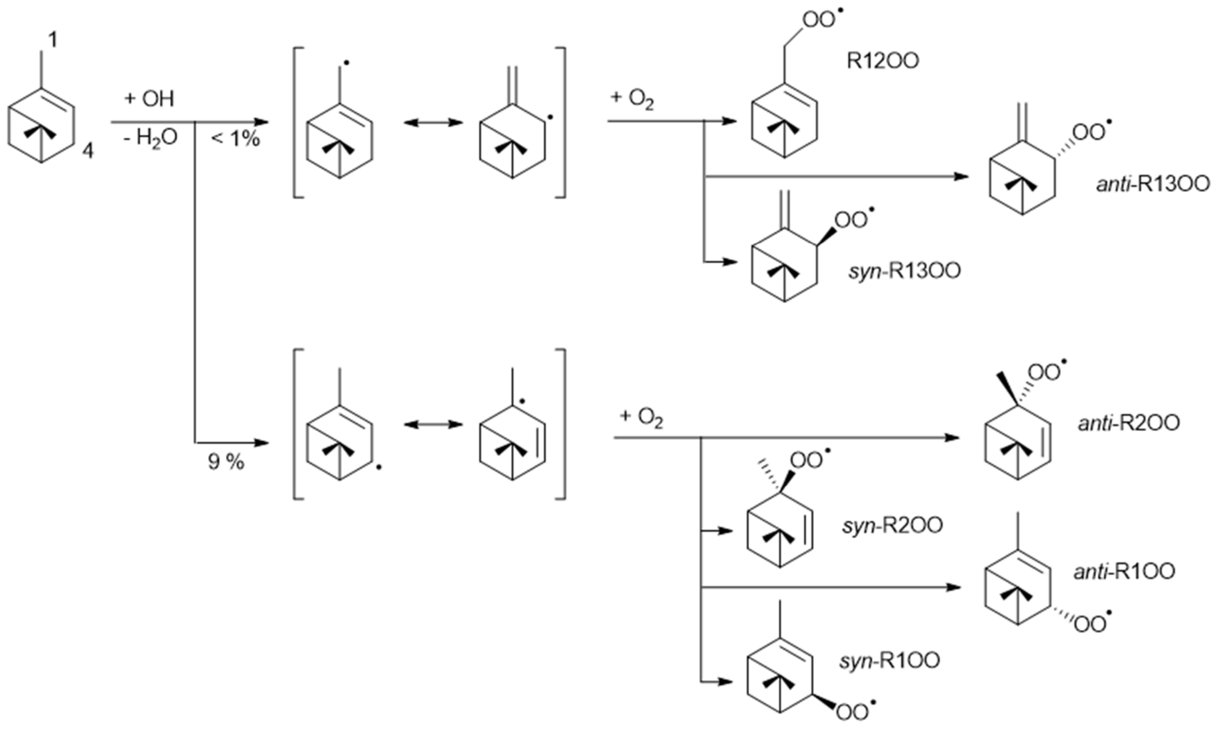

Fig. S14. Peroxy radicals formed from α-pinene H-abstraction. Seven peroxy radicals formed from hydrogen abstraction from α-pinene and following O_2 addition.

For each of these RO_2• radicals, we examined all geometrically accessible intramolecular H-migrations; ring closure reactions leads to strained tricyclic structures and are either geometrically not feasible or energetically not competitive even without the additional ring strain of the bicyclic molecular frame (*56*). The calculated rate coefficients are tabulated below in Table S1. As can be seen, all unimolecular reactions are slow, and not competitive with bimolecular reactions under the experimental conditions, where RO_2• lifetimes are of the order of 1 minute or less ($\leq 10^2$ s).

Table S1. H-migrations in RO_2• radicals formed from α-pinene H-abstraction, listing barrier heights E_b (kcal mol^{-1}), rate coefficient k (s^{-1}) at 298 K, and parameters for a Kooij equation fit $k(T)=A\times T^n\times exp(-E_a/T)$ for the temperature range 200-450 K, calculated using the CCSD(T)//M06-2X-D3 level of theory.

**RO_2•**	**Reaction type**	E_b	k**(298K)**	A / s^{-1}	n	E_a / K
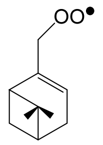 R12OO	1,7-H-shift	23.9	5.4×10^{-4}	1.17E-88	31.73	-4234
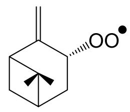 *anti*-R13OO	1,6-H-shift	28.2	5.9×10^{-6}	4.16E-111	39.57	-4988
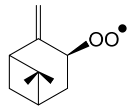 *syn*-R13OO	1,7-H-shift	29.0	7.6×10^{-7}	2.52E-117	41.56	-5258
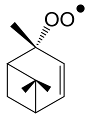 *anti*-R2OO	1,5-H-shift	25.0	2.0×10^{-4}	9.13E-99	35.81	-3944
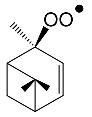 *syn*-R2OO	1,6-H-shift	26.0	9.9×10^{-5}	4.98E-103	37.07	-4529
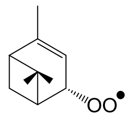 *anti*-R1OO	1,5-H-shift	27.4	4.7×10^{-6}	6.12E-111	39.63	-4695
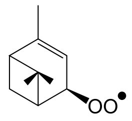 *syn*-R1OO	1,6-H-shift	26.8	2.8×10^{-5}	1.03E-105	37.75	-4824

Section S10. Theoretical kinetic study of RO_2• radicals formed from R1OO and R2OO

Due to the long unimolecular lifetime of the R1OO and R2OO peroxy radicals, they are likely to undergo bimolecular reactions, where reactions with other peroxy radicals or with NO will yield alkoxy radicals in significant fractions (*100*). The formation routes of RO_2• radicals formed from R1OO and R2OO are shown in Fig. S15. *Anti*-R2O will readily open the 6-membered ring, k ~ 2×10^5 s^{-1} (*28*), forming a cyclobutyl radical that reacts with O_2 under atmospheric conditions, forming *syn*- and *anti*-R4OO. *Syn*-R2O is expected to undergo a 1,5-H-migration, k ~ 3×10^6 s^{-1} (*28*) forming a chemically excited alkyl radical which, due to the ring strain in the 4-membered ring, is expected to undergo ring opening for a large fraction. The resulting cyclo-allyl radical will then add O_2 on either side of the ring, forming *syn*- and *anti*-R5OO and *syn*- and *anti*-R14OO. The fate of R1O is not discussed in detail. We provide a partial reaction scheme in the main text, and in Fig. S15C, S15D.

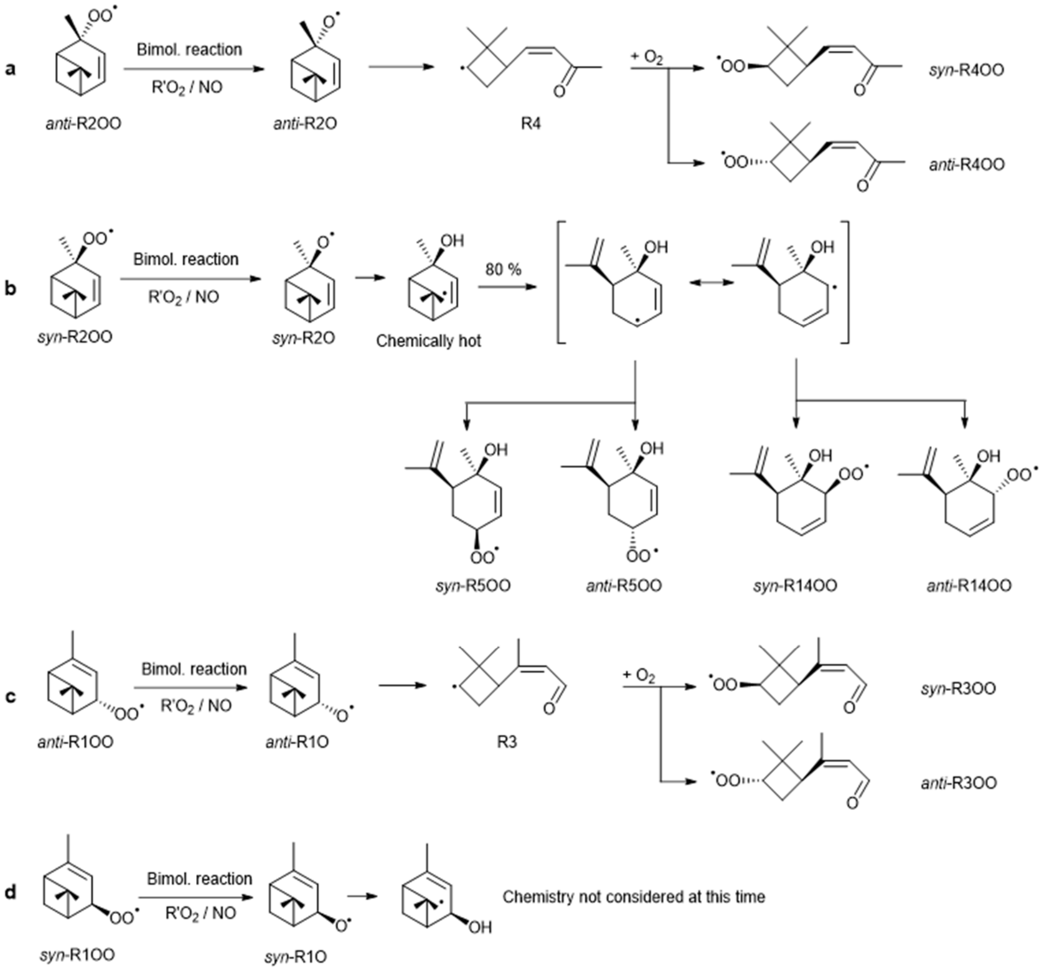

Fig. S15. Peroxy radicals formed from R1OO and R2OO. The formation pathway of peroxy radicals from (**A**) *anti*-R2OO, (**B**) *syn*-R2OO, (**C**) *anti*-R1OO, and (**D**) *syn*-R1OO with one alkoxy step and subsequent O_2 addition are shown.

We have performed theoretical kinetic calculations on the fate of the R3OO, R4OO, R5OO and R14OO radicals, which can undergo H-migration reactions and ring closure reactions. The calculation results are tabulated in Table S2. As can be seen, only unimolecular reactions in *syn*-R14OO and the fast 1,8-H-migration in *syn*-R3OO (included in Fig. 2 in the main text) are competitive against bimolecular reactions under the current reaction conditions with RO_2• lifetimes of less than 1 min. The majority of the peroxy radicals are thus expected to undergo bimolecular reactions, which for a significant fraction will yield the corresponding alkoxy radicals (R3O, R4O, R5O, and R14O, shown in Fig. S16). Their fate can be estimated using established structure-activity relationships and available theoretical work (*51, 101, 102*). The dominant loss process for R3O and R4O radicals will be the opening of the 4-membered ring, forming R6OO and R10OO molecules, respectively, after reaction with O_2 (see Fig. S16). Similarly, R14O radicals will instantly open the 6-membered ring, forming an α-OH alkyl radical that reacts readily with O_2 to form a ketone + HO_2•, terminating the radical chain. For R5O radicals we estimate that the ring structure will hamper H-migration and ring closure reactions, leaving the favorable opening of the 6-membered ring as the dominant fate, forming R11OO after O_2 addition.

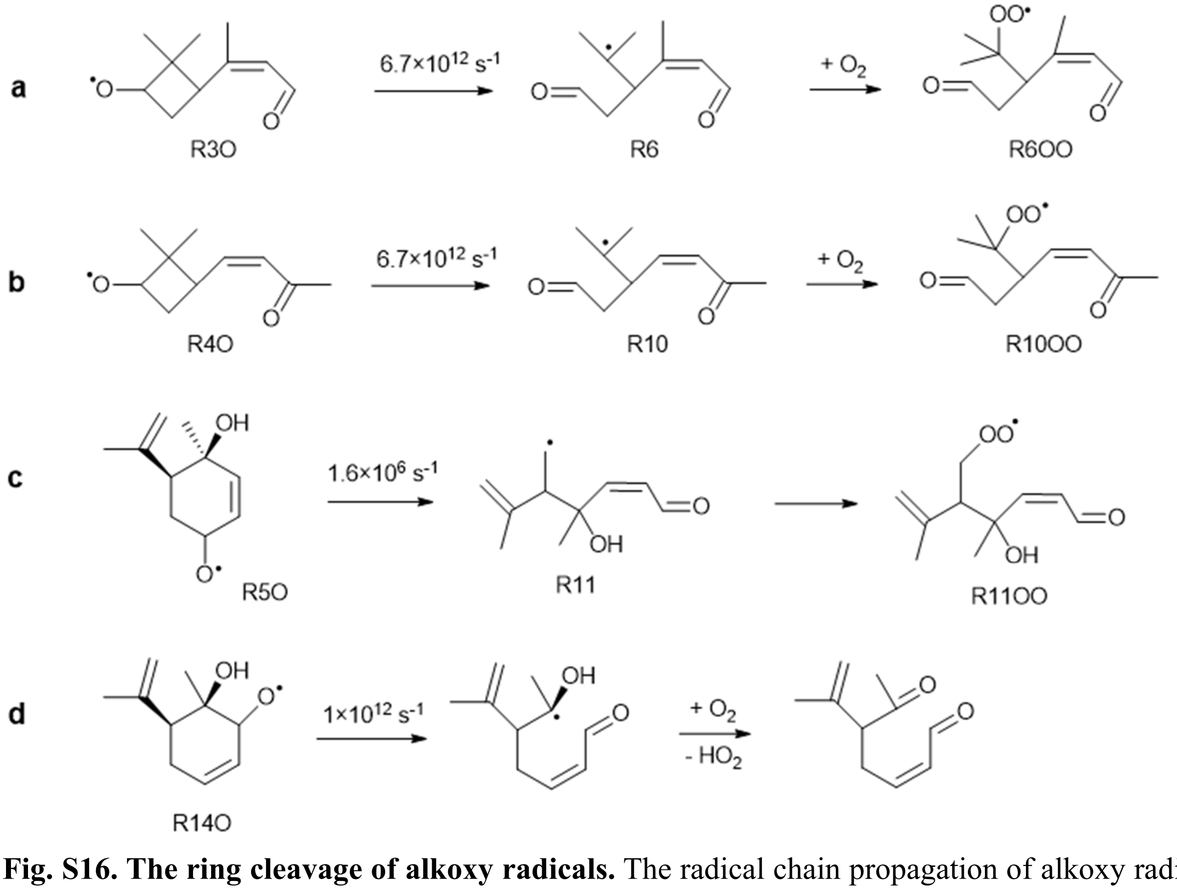

Fig. S16. The ring cleavage of alkoxy radicals. The radical chain propagation of alkoxy radials of (**A**) R3O, (**B**) R4O, and (**C**) R5O, and radical termination pathway of alkoxy radical (**D**) *anti*-R14O. Reaction rates calculated as described in Vereecken and Peeters (*51*).

Table S2. H-migrations in RO_2• radicals formed from α-pinene H-abstraction after a first alkoxy stage, listing barrier heights E_b (kcal mol^{-1}), rate coefficient k (s^{-1}) at 298 K, and parameters for a Kooij equation fit $k(T)=A\times T^n\times exp(-E_a/T)$ for the temperature range 200-450 K, calculated using the CCSD(T)//M06-2X-D3 level of theory.

RO_2•	Reaction type	E_b	k(298K)	A / s^{-1}	n	E_a / K
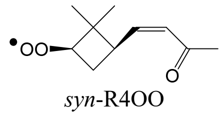 *syn*-R4OO	1,5-H-shift (prim.)	23.5	6.9×10^{-4}	1.97E-92	33.52	-3852
	1,9-H-shift (vinoxy)	29.8	4.1×10^{-8}	2.81E-114	39.64	-5554
	6-ring closure	21.7	6.0×10^{-5}	9.98E2	2.51	9223
	7-ring closure	30.4	4.8×10^{-12}	3.03E+07	0.84	14326
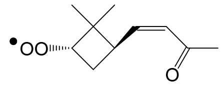 *anti*-R4OO	1,5-H-shift (prim.)	24.1	2.1×10^{-3}	3.89E-90	32.95	-3586
	1,5-H-shift (tert.)	31.8	2.9×10^{-6}	1.33E-102	35.97	-5034
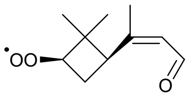 *syn*-R3OO	1,5-H-shift (prim.)	23.4	1.7×10^{-4}	1.06E-91	33.60	-3504
	1,7-H-shift (allyl)	26.3	6.8×10^{-5}	1.58E-93	33.09	-4638
	1,8-H-shift (aldeh.)	19.8	2.8×10^{-1}	3.86E-68	25.21	-3084
	6-ring closure	26.1	2.5×10^{-8}	2.98E-09	6.40	10242
	7-ring closure	30.0	4.2×10^{-11}	1.87E+08	0.93	14384
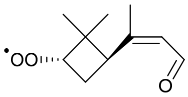 *anti*-R3OO	1,5-H-shift (prim.)	23.3	4.4×10^{-3}	1.95E-88	32.51	-3372
	1,5-H-shift (tert.)	30.1	4.6×10^{-6}	7.48E-107	37.67	-5208
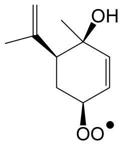 *syn*-R5OO	6-ring closure	19.8	1.9×10^{-4}	4.16E-01	3.56	8340
	7-ring closure	18.8	3.8×10^{-3}	1.08E+07	1.20	8521
	1,7-H-shift (allyl)	22.3	4.0×10^{-3}	9.17E-80	28.75	-3788
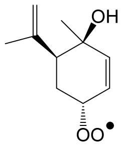 *anti*-R5OO	1,5-H-shift (tert.)	232	7.1×10^{-3}	1.69E-84	30.90	-3566
	1,7-H-shift (prim)	34.1	2.6×10^{-10}	6.37E-139	48.21	-6406
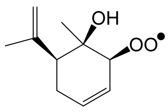 *syn*-R14OO	6-ring closure	16.9	3.3×10^{-2}	3.55E+05	1.51	7390
	7-ring closure	17.8	1.8×10^{-2}	4.71E+09	0.28	8312
	1,7-H-shift (allyl)	20.5	2.7×10^{-2}	4.30E-71	25.88	-3274
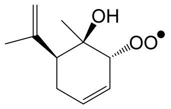 *anti*-R14OO	1,5-H-shift (prim.)	25.3	1.2×10^{-4}	4.15E-101	36.48	-4267
	1,5-H-shift (tert.)	23.2	7.8×10^{-3}	5.78E-88	32.04	-4028

Section S11. Procedure for constructing oxidation schemes after ring opening

Explicit theoretical calculations on the intermediates get increasingly costly as more oxygen atoms are added. At this time, we chose not to do such calculations for intermediates where both rings are opened. Instead, we anticipate that in the absence of ring strain the rate of reactions can be fairly well described based on structure-activity relationships (SARs).

For most H-migrations in RO_2• radicals, we base ourselves on the SAR by Vereecken and Nozière (*50*); this SAR was reported to reproduce the scarce experimental data within a factor of 2, but for multi-functionalized species such as studied in this work the scatter on the data within each SAR category reaches an order of magnitude. For those reaction classes that are not covered by the SAR, we attempt to extrapolate the reactivity trends in that SAR. For example: to estimate the rate of a 1,8-H-migration of an aldehyde H-atom (not covered by the SAR), we could (a) use the rate of an alkyl-1,8-H-migration, augmented by the typical enhancement for an aldehyde H-atom; (b) use the rate of an aldehyde-1,7-H-shift reduced by the difference in rate between an alkyl-1,7- and alkyl-1,8-H-shift; or (c) do both these extrapolations and average the predictions. Such extrapolations are not overly accurate and imply a certain level of arbitrariness in how the extrapolation is done; the expected uncertainty increases by at least an order of magnitude as no reliable data is available to validate the extrapolation. In most cases, however, this is sufficient to determine whether the reaction can contribute or not, as reaction rates typically differ by several orders of magnitude and the SAR covers the most common and favorable reaction classes explicitly. For H-migrations in cycloperoxides, we additionally rely on the systematic study by Vereecken *et al.* (*56*), who explicitly calculated rate coefficients for peroxy radicals formed after RO_2• ring closure reactions. For ring closure reactions in unsaturated RO_2• we rely on the SAR by Vereecken *et al.* (*56*). We assume that the rate of ring closure is not affected by the presence of another cycloperoxide ring; this approximation is unlikely to be accurate, but insufficient data is available to improve the estimates.

In assessing the fate of an RO_2• radical with one or more -OOH groups, we account for the possibility of H-atom scrambling, as described extensively in the literature (*53-55, 99, 103*). The H-migrations between the -OO• and -OOH groups often allow for additional pathways, and are typically much faster than other unimolecular channels, such that the scrambling leads to a fast equilibration among all accessible OOH-substituted RO_2• radicals, and the dominant channel is chosen among those available to the pool of RO_2• radicals. We refer to Vereecken and Nozière (*50*) for a more detailed description of this feature.

In the Fig. S17, we show some explicit examples on how this approach work. For each intermediate, we examine all possible unimolecular reaction channels, estimate its rate coefficient, and select the dominant pathway (shown in bold) as the reactive channel. Following these examples, autoxidation pathways subsequent to R6OO, R9OO, R10OO, and R11OO (Fig. 2 in the main text) were determined and shown in Fig. S18.

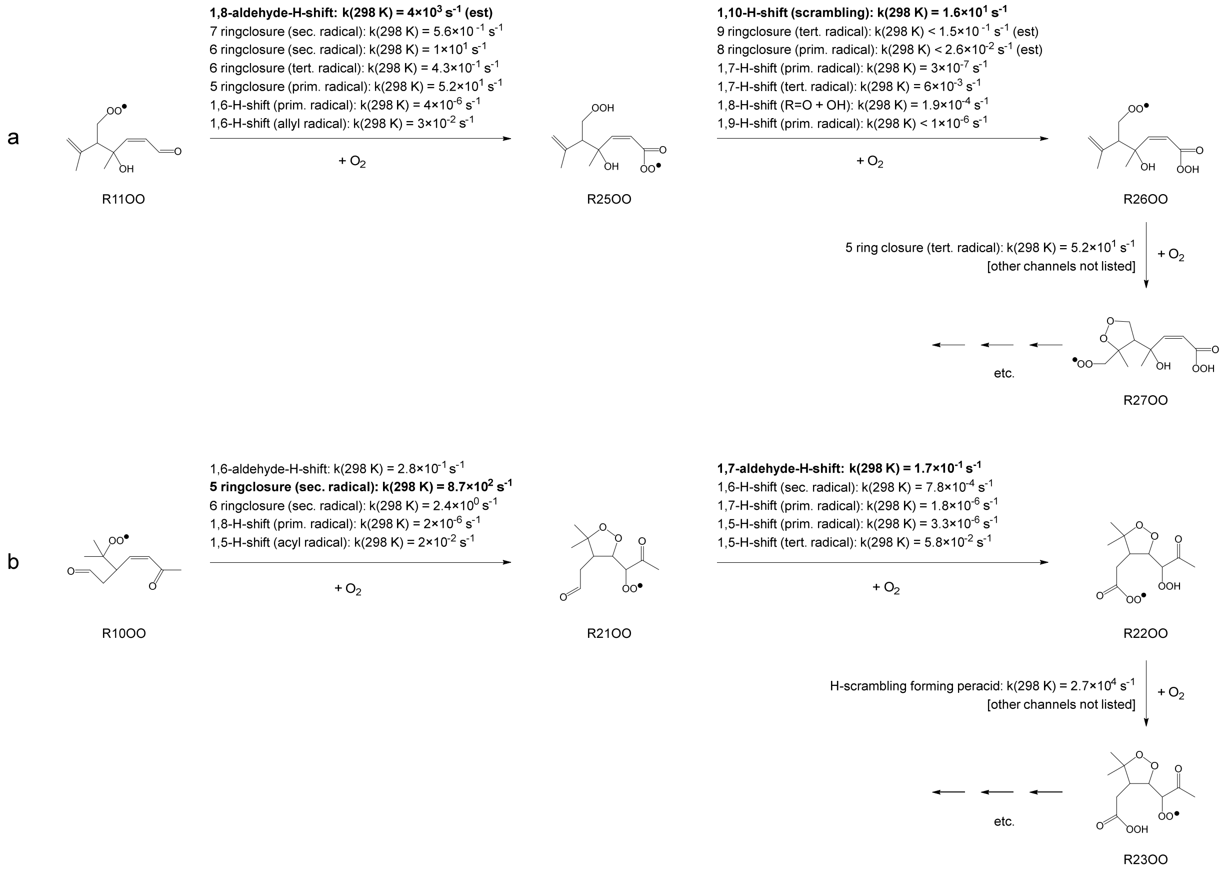

Fig. S17. Determination of the unimolecular reactions of peroxy radical intermediates. Two explicit examples to show the determination approach of the unimolecular reactions for (**A**) R11OO and (**B**) R10OO. All possible unimolecular reaction channels of each peroxy intermediates are listed and the dominant pathway is highlighted in bold. The details of how rate coefficients are determined can be referred to the text in the page above (Section S11).

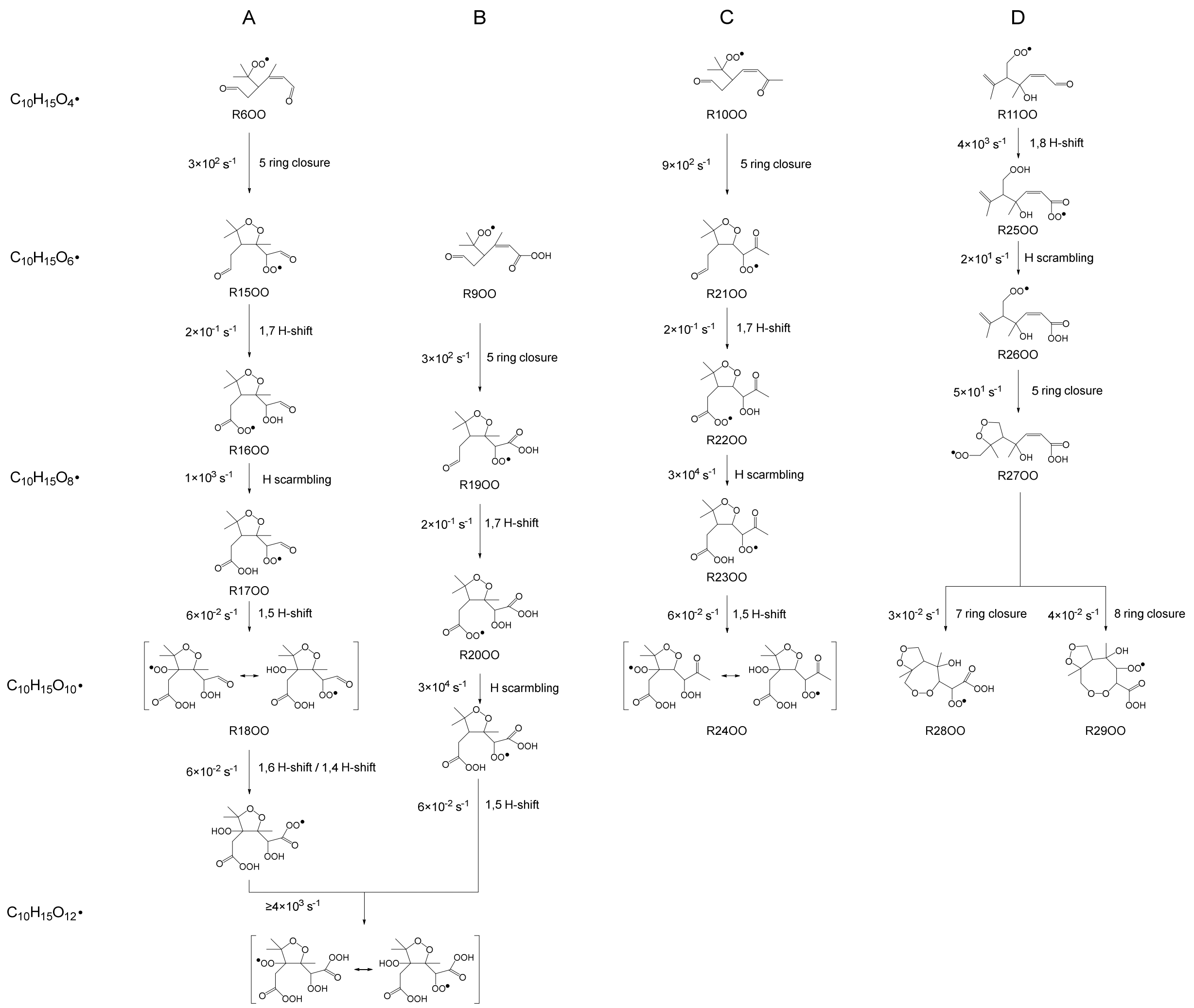

Fig. S18. Subsequent autoxidation pathways of $C_{10}H_{15}O_X$•. Example autoxidation pathways of (**A**) R6OO, (**B**) R9OO, (**C**) R10OO, and (**D**) R11OO. The formation pathway of R6OO, R9OO, R10OO, and R11OO are shown in Fig. 2 in the main text. The chemical formulas of peroxy radicals are denoted on the left side of the figure. Note that alkoxy steps are not included in the figure. All of the rate coefficients shown in this figure were derived from SARs, as described in Section S11.

Section S12. Incorporation of the hydrogen abstraction channel in MCM

The hydrogen abstraction channel is not included in the α-pinene + OH chemistry in the current MCM v3.3.1 (*48*). Based on the calculated reaction rates and mechanism in Fig. 2 in the main text, we added the hydrogen abstraction channel to the MCM (http://mcm.york.ac.uk/home.htt), with detailed reactions and rate coefficients listed in Table S3, which account for the site- and stereo-specific data from the theoretical study described above. In the initial step of α-pinene + OH, a branching ratio of 0.09 is applied to the hydrogen abstraction channel (reactions 4-7 in Table S3) and the branching ratio of OH addition channel is also updated (reactions 1-3 in Table S3). The main loss of RO_2• radical is assigned to bimolecular reactions with NO to produce organic nitrates (10%) and alkoxy radicals (90%). Bimolecular reactions of RO_2• + HO_2• are also included, but bimolecular reactions of RO_2• + RO_2• and OH elimination by unimolecular RO_2• reactions are not included. Reactions for the hydrogen abstraction channel start at α-pinene ($C_{10}H_{16}$) + OH and include the formation of peroxy radicals containing up to ten oxygen atoms ($C_{10}H_{15}O_{10}$•). Besides the RO_2• autoxidation reactions shown in Fig. 2 in the main text and Fig. S18, all other reactions with rate coefficients more than 1×10^{-3} s^{-1} are also included for completeness. While we do not have theoretical calculations for *syn*-R1OO/*syn*-R1O, which accounts for about 20% of the hydrogen abstraction channel, similar chemistry is assumed for *syn*-R1O to *syn*-R2O, shown as reactions of 33-34 and 49-50 in Table S3. Totally, 139 reactions were included for calculations, including peroxy radicals from R1OO to R29OO in this study.

The MCM simulations were calculated using an open access program iChamber (*104*). Production and photolysis of HONO were also incorporated into the calculations according to Rohrer *et al.* (*52*). Physical parameters, including J_{HONO}, J_{NO2}, temperature, and relative humidity, were constrained to measured data with a time resolution of 10 s. In both low and high NO experiments, initial concentrations of α-pinene were set as 20 ppb. Initial concentrations of NO were set as 0 ppb at low NO and 20 ppb at high NO. A reaction period of 15 min was calculated for both low NO and high NO using the above settings. Condensation sink of HOM to particles is negligible due to the absence of nucleation in this time frame. The wall loss rate of 6×10^{-4} s^{-1} (low NO) and 2.2×10^{-3} s^{-1} (high NO) were applied to HOM with six and more oxygen atoms, and a dilution rate of 1.5×10^{-5} s^{-1} was applied to all compounds in our MCM calculations.

The closed-shell product $C_{10}H_{15}NO_9$ is used to compare our calculated results to the observations (CIMS data). These $C_{10}H_{15}NO_9$ species are formed from $C_{10}H_{15}O_8$•. The rate coefficients of RO_2• + NO → $RONO_2$ are known, and organic nitrate formation is included in our calculations. Moreover, in both low and high NO experiments, the observed concentrations of $C_{10}H_{15}NO_9$ are sufficiently high for comparison, with an abundance of 11.3% (low NO) and 27.0% (high NO) relative to the highest closed-shell products $C_{10}H_{14}O_{10}$. In our calculations, $C_{10}H_{15}NO_9$ are formed in reactions 115, 118, 999, 127, 130, and 139 as listed in Table S3. Their calculated time series and CIMS data at low and high NO are shown in Fig. S19. Our simulations agree within the uncertainties with the observations at both low and high NO. The formation of $C_{10}H_{15}NO_9$

accelerates after around 5 min reaction time at low NO, while $C_{10}H_{15}NO_9$ formation starts after about 2 min reaction time at high NO. Note that as mentioned in Section S2, some background NO concentration was present in the low NO experiments; thus a sensitivity test of initial NO concentrations of 0-30 ppt was also conducted for low NO experiment, with dashed lines shown in Fig. S19A. With each increase in the initial NO concentration, it takes less time to accelerate the formation of $C_{10}H_{15}NO_9$. This is consistent with the expectation that higher NO concentrations promote the production of alkoxy radicals. As shown in Fig. S19A, all simulated $C_{10}H_{15}NO_9$ (with an initial NO concentration from 0-30 ppt) are within the standard errors of the observations. The background NO concentrations have negligible influence on our experiments.

The alkoxy production rate was estimated based on the reaction of RO_2• + NO → RO• + NO_2 in this study, as shown in Fig. S20. Concentrations of RO_2• and NO were derived from our MCM simulations and a uniform rate constant of k_{RO2+NO} was used. The organic nitrate yield is assumed to be 0.1. Overall, a lower alkoxy production rate was observed in low NO than in high NO. Also, as expected, the formation of alkoxy radicals is slow at the beginning of the low NO experiment while immediate formation of alkoxy radicals is observed at high NO. This again emphasizes the requirement for ring opening, and NO dependence in the HOM formation from H-abstraction channel in α-pinene + OH reactions in this study.

We also examined the sensitivity of the model results to the model parameters. Two sets of the rate coefficients were applied, including one set derived from explicit calculations in this study (as discussed above), and the other set assigned as fixed values (*50, 56*), as shown in Table S3. Calculated time series of $C_{10}H_{15}NO_9$ using explicit and fixed rate coefficients are shown in Fig. S19. Overall, no significant difference is observed in concentrations of $C_{10}H_{15}NO_9$ using explicit and fixed values, and the delayed $C_{10}H_{15}NO_9$ formation in low NO are both observed using explicit and fixed values. Hence, the model results are not overly sensitive to the rate coefficients used. However, without knowing reaction pathways and chemical structures, which are still derived from explicit calculations, the fixed values cannot be assigned. In this case, it is essential to determine the reaction pathways based on explicit calculations to simulate product formation in the MCM. Further work is needed to incorporate more detailed H-abstraction channel in MCM.

To further compare $C_{10}H_{15}O_X$• and $C_{10}H_{17}O_X$• chemistry in the HOM formation from OH oxidation of α-pinene, autoxidation pathway of $C_{10}H_{17}O_X$• was added to the MCM calculations, with reactions in Table S4 following Xu *et al.* (*27*). In OH addition channel, autoxidation is expected to only occur in the ring-opened RO_2• ($C_{10}H_{17}O_3$•, Fig. S12A). Reactions after the ring-opened RO_2• (>1×10^{-3} s^{-1}), from $C_{10}H_{17}O_3$• to $C_{10}H_{17}O_{11}$•, were incorporated into the calculations, including Scheme 2, 3, and 5 in Xu *et al.* (*27*). Note that reactions leading to low oxygenated termination products (O<6), such as P2 in their Scheme 2, are not included to simplify the model. For rate coefficients or branching ratios missing in Xu *et al.* (*27*), uniform values were applied. For example, Scheme 5 in their paper, only *anti*-$C_{10}H_{17}O_3$• can undergo subsequent rapid autoxidation. A stereospecific branching ratio of 50% was applied to α-pinene+OH, as shown

reactions 1 and 2 in Table S4. The rate coefficients of RO_2• H-shifts were estimated from Vereecken and Nozière (*50*). Rate coefficients of ring-closure, H scrambling, RO• decomposition, and RO• H-shift, were given as 1 s^{-1}, 1×10^{2} s^{-1}, KDEC (1×10^{6} s^{-1}), and 1×10^{5} s^{-1} according to empirical knowledge from literature (*54-56*). Organic nitrate $C_{10}H_{17}NO_8$ was used to compare our calculated results to the observations, as shown in Fig. S21. Overall, our calculation results agree within the uncertainties with the observations at both low and high NO. Similar time series were observed at low NO and high NO and no delay was observed at low NO. This is consistent with independence of $C_{10}H_{17}O_X$• formation pathway on NO.

As only part of the $C_{10}H_{15}O_X$• ($X\leq10$) and $C_{10}H_{17}O_X$• ($X\leq11$) chemistry was incorporated into the MCM calculations, $C_{10}H_{17}NO_8$ and $C_{10}H_{15}NO_9$ are used as an example to compare $C_{10}H_{15}O_X$• and $C_{10}H_{17}O_X$• chemistry in the HOM formation from OH oxidation of α-pinene. They are formed via similar formation pathway, i.e., two autoxidation steps and following bimolecular reactions with NO. Their concentration ratios from MCM calculations are shown in Fig. S22. At low NO, concentration ratios of $C_{10}H_{15}NO_9$ to $C_{10}H_{17}NO_8$ increased with reaction time. This is expected as a result of an accelerated $C_{10}H_{15}O_X$• chemistry and $C_{10}H_{17}O_X$• chemistry independent of NO as reaction proceeds, with increasing NO from HONO photolysis. Higher initial NO concentration leads to higher concentration ratio of $C_{10}H_{15}NO_9$ to $C_{10}H_{17}NO_8$, also supporting the acceleration of $C_{10}H_{15}O_X$• chemistry by NO. At high NO, concentration ratio of $C_{10}H_{15}NO_9$ to $C_{10}H_{17}NO_8$ is relatively stable, with a quick rise at the start of the reaction and a slight decrease over later reaction times. As high NO concentrations lead to rapid alkoxy steps, $C_{10}H_{15}O_X$• chemistry is expected to dominate over $C_{10}H_{17}O_X$• chemistry and to lead to a relatively stable concentration ratio of $C_{10}H_{15}NO_9$ to $C_{10}H_{17}NO_8$. This time series pattern is similar to the observed time series of $C_{10}H_{15}NO_X$ and $C_{10}H_{17}NO_X$ (Fig. 3B, 3D), with an increasing concentration ratio of $C_{10}H_{15}NO_X$ to $C_{10}H_{17}NO_X$ at low NO and stabilization at low NO. However, it takes more time for observed concentration ratios (~5 min) to achieve stabilization than in MCM calculations (almost immediately after the reactions). This difference may be attributed to the incomplete mechanism of $C_{10}H_{15}O_X$• chemistry, particularly for different chemistry of various isomers as only the most likely pathway is proposed in this study, and to incomplete $C_{10}H_{17}O_X$• chemistry where only example pathways are available in the literature (*27*) and included in MCM. Part of the difference can also attribute to large uncertainties of observed concentration ratios (Fig. 3). Further studies are warranted for more detailed H-abstraction channel in OH oxidation of α-pinene.

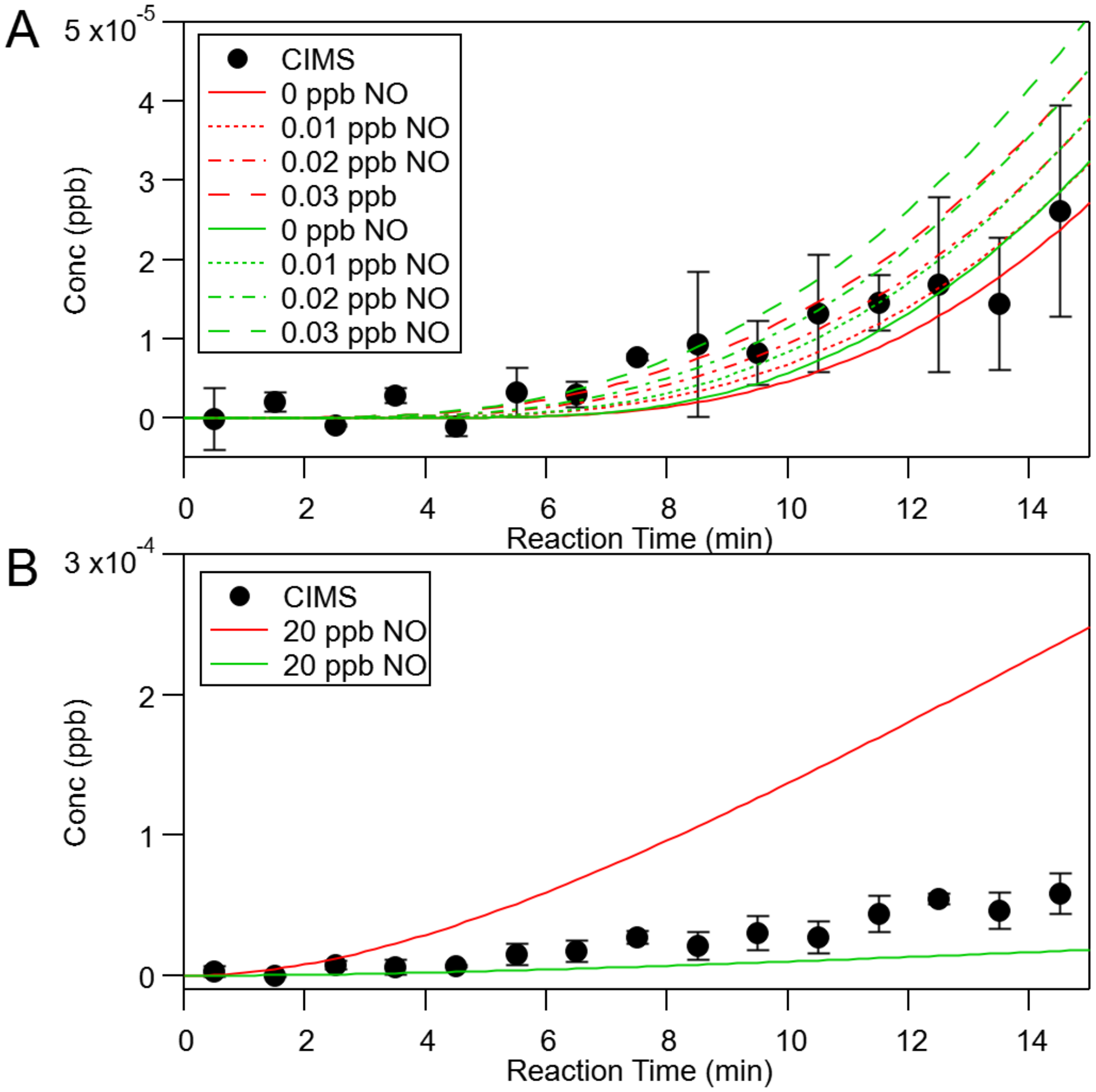

Fig. S19. Time series of calculated $C_{10}H_{15}NO_9$ concentration and measured one using CIMS. Based on our modified MCM mechanism of α-pinene + OH, time series of $C_{10}H_{15}NO_9$ are calculated in (**A**) low NO (with an initial NO concentration of 0 ppb, 0.01 ppb, 0.02 ppb, and 0.03 ppb) and (**B**) high NO experiments (with an initial NO concentration of 20 ppb). Calculations using explicit reaction rates for individual peroxy radical are red lines; calculations using fixed reaction rates are green lines. CIMS data of $C_{10}H_{15}NO_9$ are denoted as black markers with one standard deviation as error bars.

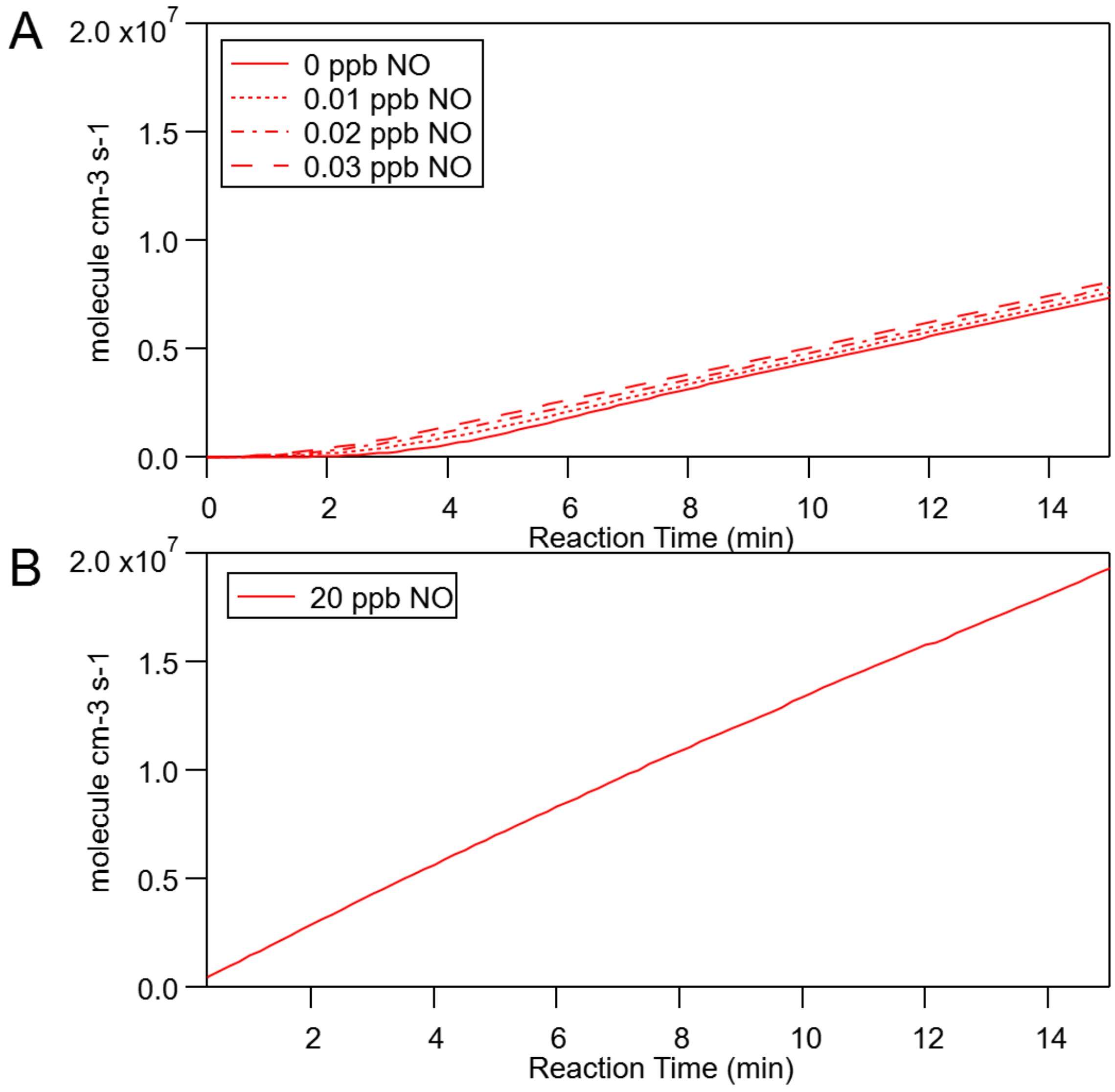

Fig. S20. Production rate of alkoxy radicals at low and high NO experiments. Based on reaction of RO_2• + NO → RO• + NO_2, the production rate of alkoxy radicals is estimated in (**A**) low NO and (**B**) high NO experiments, using organic nitrate yields (0.1) and concentrations of RO_2• and NO from MCM results. Note that the vertical axes of the two panels are on the linear scale with a range of 0-2.0×10^7 molecule cm^{-3} s^{-1}.

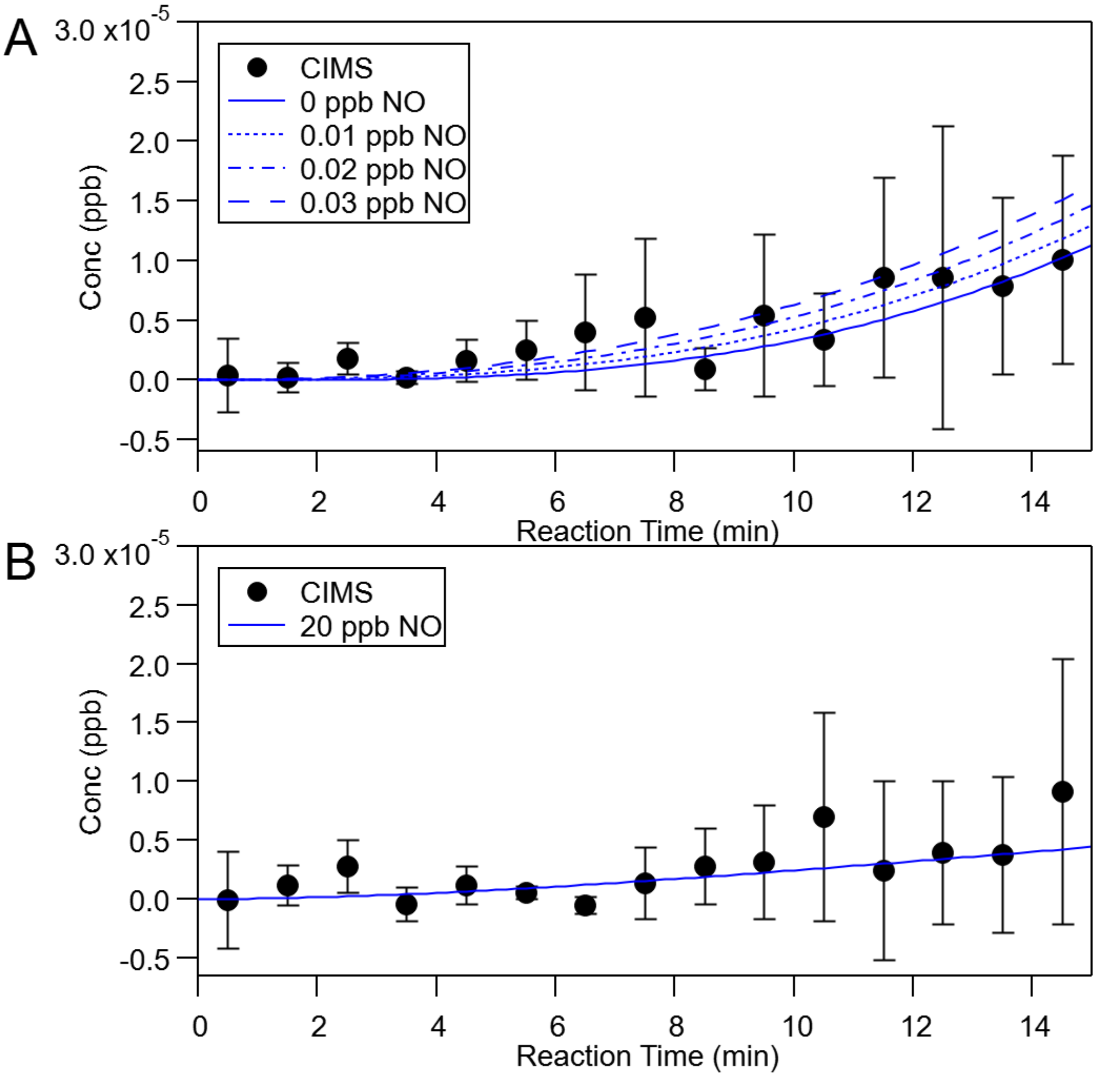

Fig. S21. Time series of calculated $C_{10}H_{17}NO_8$ concentration and measured one using CIMS. Based on our MCM mechanism of α-pinene + OH following Xu *et al.* (*27*), time series of $C_{10}H_{17}NO_8$ are calculated in (**A**) low NO (with an initial NO concentration of 0 ppb, 0.01 ppb, 0.02 ppb, and 0.03 ppb) and (**B**) high NO experiments (with an initial NO concentration of 20 ppb). CIMS data of $C_{10}H_{17}NO_8$ are denoted as black markers with one standard deviations as error bars.

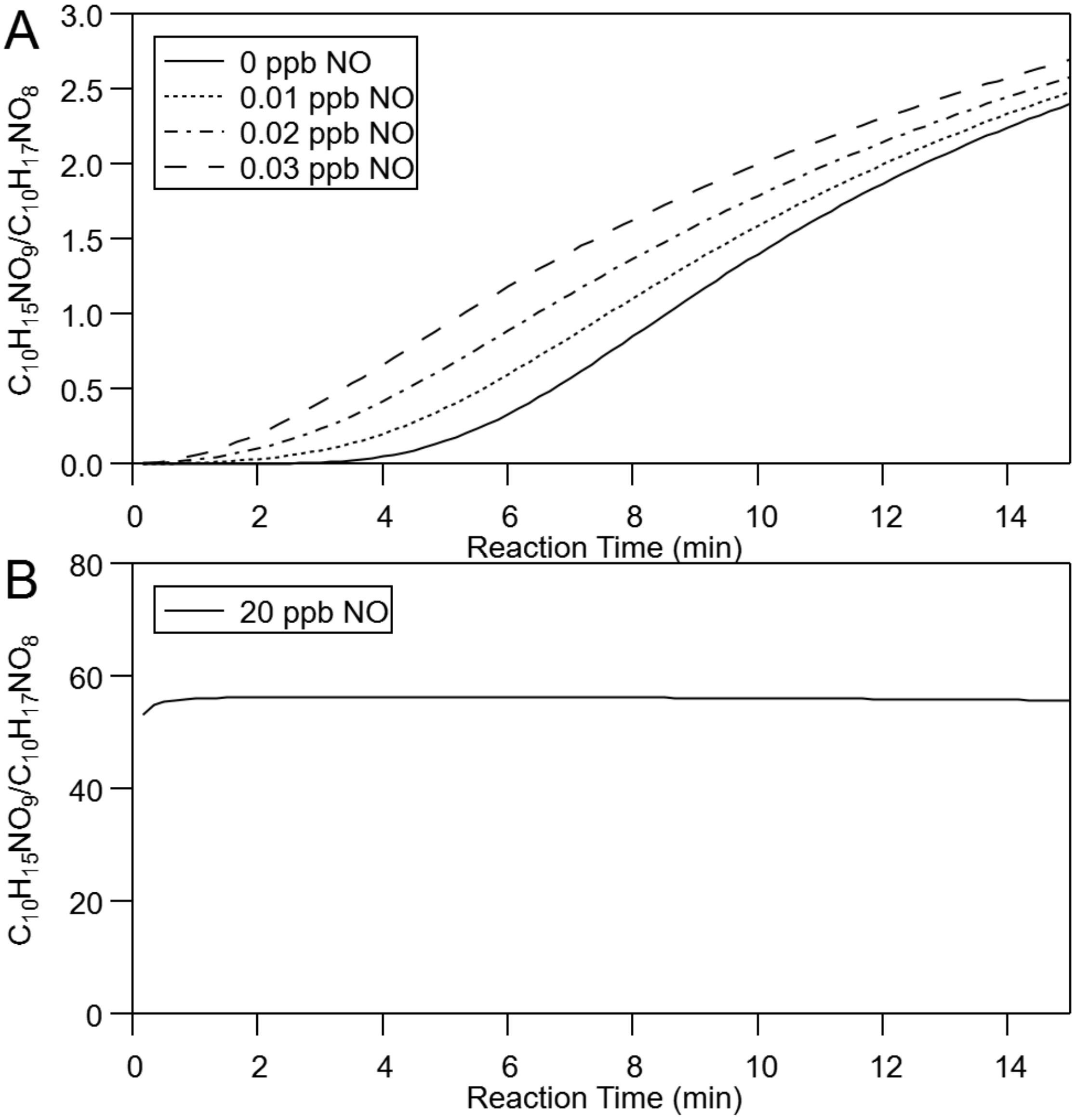

Fig. S22. Time series of calculated concentration ratio of $C_{10}H_{15}NO_9/C_{10}H_{17}NO_8$. Based on our MCM mechanism of α-pinene + OH following Xu *et al.* (*27*), time series of concentration ratio of $C_{10}H_{15}NO_9/C_{10}H_{17}NO_8$ are calculated in (**A**) low NO (with an initial NO concentration of 0 ppb, 0.01 ppb, 0.02 ppb, and 0.03 ppb) and (**B**) high NO experiments (with an initial NO concentration of 20 ppb).

Table S3. Hydrogen abstraction mechanism added to α-pinene + OH subset of MCM. Incorporated reactions and rate coefficients in the default α-pinene + OH subset of MCM mechanism. Two sets of rate coefficients were used, including one set of values derived from explicit calculations in this study and another one assigned as fixed values.

**No.**	**Reactions**	**Explicit rate coefficients (1st order reactions: s^{-1} 2nd order reactions: cm^3 $molecule^{-1}$ s^{-1})**	**Fixed rate coefficients (1st order reactions: s^{-1} 2nd order reactions: cm^3 $molecule^{-1}$ s^{-1})**
1	APINENE + OH = APINAO2	1.2E-11*EXP(440/TEMP)*0.91*0.572	1.2E-11*EXP(440/TEMP)*0.91*0.572
2	APINENE + OH = APINBO2	1.2E-11*EXP(440/TEMP)*0.91*0.353	1.2E-11*EXP(440/TEMP)*0.91*0.353
3	APINENE + OH = APINCO2	1.2E-11*EXP(440/TEMP)*0.91*0.075	1.2E-11*EXP(440/TEMP)*0.91*0.075
4	APINENE + OH = syn-R1OO	1.2E-11*EXP(440/TEMP)*0.09*0.4*0.5	1.2E-11*EXP(440/TEMP)*0.09*0.4*0.5
5	APINENE + OH = anti-R1OO	1.2E-11*EXP(440/TEMP)*0.09*0.4*0.5	1.2E-11*EXP(440/TEMP)*0.09*0.4*0.5
6	APINENE + OH = syn-R2OO	1.2E-11*EXP(440/TEMP)*0.09*0.6*0.4	1.2E-11*EXP(440/TEMP)*0.09*0.6*0.4
7	APINENE + OH = anti-R2OO	1.2E-11*EXP(440/TEMP)*0.09*0.6*0.6	1.2E-11*EXP(440/TEMP)*0.09*0.6*0.6
8	anti-R1O = syn-R3OO	6.3E+06*0.5	KDEC*0.5
9	anti-R1O = anti-R3OO	6.3E+06*0.5	KDEC*0.5
10	anti-R3OO = anti-R3OO-Others	1.3E-01 (1,5-H-shift)	1.0E-02
11	syn-R3O = R6OO	6.7E+12	KDEC
12	anti-R3O = R6OO	6.7E+12	KDEC
13	R6OO = R15OO	3.3E+02	1.0E+00
14	R15OO = R16OO	1.7E-01	5.0E-01
15	R16OO = R17OO	1.1E+03	1.0E+02
16	R17OO = R18OO	5.8E-02	1.0E-02
17	syn-R3OO = R7OO	1.3E-01	5.0E-01
18	R7OO = R8OO	1.6E+01	1.0E+02
19	R8O = R9OO	6.7E+12	KDEC
20	R9OO = R19OO	3.3E+02	1.0E+00
21	R19OO = R20OO	1.7E-01	5.0E-01
22	anti-R2O = syn-R4OO	2.9E+08*0.5	KDEC*0.5
23	anti-R2O = anti-R4OO	2.9E+08*0.5	KDEC*0.5
24	anti-R4OO = anti-R4OO-Others	2.1E-03 (1,5-H-shift)	1.0E-02
25	syn-R4O = R10OO	6.7E+12	KDEC
26	anti-R4O = R10OO	6.7E+12	KDEC
27	R10OO = R21OO	8.7E+02	1.0E+00
		2.8E-01 (1,6-H-shift)	5.0E-01
28	R10OO = R10OO-Others	2.4E+01 (6-ring closure)	1.0E+00
		2.0E-02 (1,5-H-shift)	1.0E-02
29	R21OO = R22OO	1.7E-01	5.0E-01
30	R21OO = R21OO-Others	5.8E-02 (1,5-H-shift)	1.0E-02
31	R22OO = R23OO	2.7E+04	1.0E+02
32	R23OO = R24OO	5.8E-02	1.0E-02

33	syn-R1O = syn-R5OO	3.0E+06*0.5*0.5	KDEC*0.5*0.5
34	syn-R1O = anti-R5OO	3.0E+06*0.5*0.5	KDEC*0.5*0.5
35	syn-R2O = syn-R5OO	3.0E+06*0.5*0.5	KDEC*0.5*0.5
36	syn-R2O = anti-R5OO	3.0E+06*0.5*0.5	KDEC*0.5*0.5
37	syn-R5OO = syn-R5OO-Others	3.8E-03 (7-ring closure)	1.0E+00
		4.0E-03 (1,7-H-shift)	1.0E-02
38	anti-R5OO = anti-R5OO-Others	7.1E-03 (1,5-H-shift)	1.0E-02
39	syn-R5O = R11OO	1.6E+06	KDEC
40	anti-R5O = R11OO	1.6E+06	KDEC
41	R11OO = R25OO	4.0E+03	5.0E-01
42	R11OO = R11OO-Others	5.6E-01 (7-ring closure)	1.0E+00
		1.0E+01 (6-ring closure)	1.0E+00
		4.3E-02 (6-ring closure)	1.0E+00
		5.2E+01 (5-ring closure)	1.0E+00
		3.0E-02 (1,6-H-shift)	1.0E-02
43	R25OO = R26OO	1.6E+01	1.0E+02
44	R25OO = R25OO-Others	6.0E-03 (1,7-H-shift)	1.0E-02
		1.5E-01 (9-ring closure)	1.0E+00
		2.6E-02 (8-ring closure)	1.0E+00
45	R26OO = R27OO	5.2E+01	1.0E+00
46	R27OO = R28OO	2.7E-02	1.0E+00
47	R27OO = R29OO	4.5E-02	1.0E+00
48	R27OO = R27OO-Others	1.8E-03 (1,5-H-shift)	3.5E-02
		1.2E-02 (1,6-H-shift)	8.8E-04
49	syn-R1O = syn-R14OO	3.0E+06*0.5*0.5	KDEC*0.5*0.5
50	syn-R1O = anti-R14OO	3.0E+06*0.5*0.5	KDEC*0.5*0.5
51	syn-R2O = syn-R14OO	3.0E+06*0.5*0.5	KDEC*0.5*0.5
52	syn-R2O = anti-R14OO	3.0E+06*0.5*0.5	KDEC*0.5*0.5
53	syn-R14OO = syn-R14OO-Others	3.3E-02 (6-ring closure)	1.0E+00
		1.8E-02 (7-ring closure)	1.0E+00
		2.7E-02 (1,7-H-shift)	1.0E-02
54	syn-R14O = R14HO + HO2	1.0E+12	KDEC
55	anti-R14O = R14HO + HO2	1.0E+12	KDEC
56	syn-R1OO + HO2 = syn-R1OOH	KRO2HO2	KRO2HO2
57	syn-R1OO + NO = syn-R1O + NO2	KRO2NO*0.9	KRO2NO*0.9
58	syn-R1OO + NO = syn-R1ONO2	KRO2NO*0.1	KRO2NO*0.1
59	anti-R1OO + HO2 = anti-R1OOH	KRO2HO2	KRO2HO2
60	anti-R1OO + NO = anti-R1O + NO2	KRO2NO*0.9	KRO2NO*0.9
61	anti-R1OO + NO = anti-R1ONO2	KRO2NO*0.1	KRO2NO*0.1
62	syn-R2OO + HO2 = syn-R2OOH	KRO2HO2	KRO2HO2
63	syn-R2OO + NO = syn-R2O + NO2	KRO2NO*0.9	KRO2NO*0.9

64	syn-R2OO + NO = syn-R2ONO2	KRO2NO*0.1	KRO2NO*0.1
65	anti-R2OO + HO2 = anti-R2OOH	KRO2HO2	KRO2HO2
66	anti-R2OO + NO = anti-R2O + NO2	KRO2NO*0.9	KRO2NO*0.9
67	anti-R2OO + NO = anti-R2ONO2	KRO2NO*0.1	KRO2NO*0.1
68	syn-R3OO + HO2 = syn-R3OOH	KRO2HO2	KRO2HO2
69	syn-R3OO + NO = syn-R3O + NO2	KRO2NO*0.9	KRO2NO*0.9
70	syn-R3OO + NO = syn-R3ONO2	KRO2NO*0.1	KRO2NO*0.1
71	anti-R3OO + HO2 = anti-R3OOH	KRO2HO2	KRO2HO2
72	anti-R3OO + NO = anti-R3O + NO2	KRO2NO*0.9	KRO2NO*0.9
73	anti-R3OO + NO = anti-R3ONO2	KRO2NO*0.1	KRO2NO*0.1
74	syn-R4OO + HO2 = syn-R4OOH	KRO2HO2	KRO2HO2
75	syn-R4OO + NO = syn-R4O + NO2	KRO2NO*0.9	KRO2NO*0.9
76	syn-R4OO + NO = syn-R4ONO2	KRO2NO*0.1	KRO2NO*0.1
77	anti-R4OO + HO2 = anti-R4OOH	KRO2HO2	KRO2HO2
78	anti-R4OO + NO = anti-R4O + NO2	KRO2NO*0.9	KRO2NO*0.9
79	anti-R4OO + NO = anti-R4ONO2	KRO2NO*0.1	KRO2NO*0.1
80	syn-R5OO + HO2 = syn-R5OOH	KRO2HO2	KRO2HO2
81	syn-R5OO + NO = syn-R5O + NO2	KRO2NO*0.9	KRO2NO*0.9
82	syn-R5OO + NO = syn-R5ONO2	KRO2NO*0.1	KRO2NO*0.1
83	anti-R5OO + HO2 = anti-R5OOH	KRO2HO2	KRO2HO2
84	anti-R5OO + NO = anti-R5O + NO2	KRO2NO*0.9	KRO2NO*0.9
85	anti-R5OO + NO = anti-R5ONO2	KRO2NO*0.1	KRO2NO*0.1
86	R6OO + HO2 = R6OOH	KRO2HO2	KRO2HO2
87	R6OO + NO = R6O + NO2	KRO2NO*0.9	KRO2NO*0.9
88	R6OO + NO = R6ONO2	KRO2NO*0.1	KRO2NO*0.1
89	R7OO + HO2 = R7OOH	KRO2HO2	KRO2HO2
90	R7OO + NO = R7O + NO2	KRO2NO*0.9	KRO2NO*0.9
91	R7OO + NO = R7ONO2	KRO2NO*0.1	KRO2NO*0.1
92	R8OO + HO2 = R8OOH	KRO2HO2	KRO2HO2
93	R8OO + NO = R8O + NO2	KRO2NO*0.9	KRO2NO*0.9
94	R8OO + NO = R8ONO2	KRO2NO*0.1	KRO2NO*0.1
95	R9OO + HO2 = R9OOH	KRO2HO2	KRO2HO2
96	R9OO + NO = R9O + NO2	KRO2NO*0.9	KRO2NO*0.9
97	R9OO + NO = R9ONO2	KRO2NO*0.1	KRO2NO*0.1
98	R10OO + HO2 = R10OOH	KRO2HO2	KRO2HO2
99	R10OO + NO = R10O + NO2	KRO2NO*0.9	KRO2NO*0.9
100	R10OO + NO = R10ONO2	KRO2NO*0.1	KRO2NO*0.1
101	R11OO + HO2 = R11OOH	KRO2HO2	KRO2HO2
102	R11OO + NO = R11O + NO2	KRO2NO*0.9	KRO2NO*0.9
103	R11OO + NO = R11ONO2	KRO2NO*0.1	KRO2NO*0.1
104	syn-R14OO + HO2 = syn-R14OOH	KRO2HO2	KRO2HO2

105	syn-R14OO + NO = syn-R14O + NO2	KRO2NO*0.9	KRO2NO*0.9
106	syn-R14OO + NO = syn-R14ONO2	KRO2NO*0.1	KRO2NO*0.1
107	anti-R14OO + HO2 = anti-R14OOH	KRO2HO2	KRO2HO2
108	anti-R14OO + NO = anti-R14O + NO2	KRO2NO*0.9	KRO2NO*0.9
109	anti-R14OO + NO = anti-R14ONO2	KRO2NO*0.1	KRO2NO*0.1
110	R15OO + HO2 = R15OOH	KRO2HO2	KRO2HO2
111	R15OO + NO = R15O + NO2	KRO2NO*0.9	KRO2NO*0.9
112	R15OO + NO = R15ONO2	KRO2NO*0.1	KRO2NO*0.1
113	R16OO + HO2 = R16OOH	KRO2HO2	KRO2HO2
114	R16OO + NO = R16O + NO2	KRO2NO*0.9	KRO2NO*0.9
115	R16OO + NO = R16ONO2	KRO2NO*0.1	KRO2NO*0.1
116	R17OO + HO2 = R17OOH	KRO2HO2	KRO2HO2
117	R17OO + NO = R17O + NO2	KRO2NO*0.9	KRO2NO*0.9
118	R17OO + NO = R17ONO2	KRO2NO*0.1	KRO2NO*0.1
119	R19OO + HO2 = R19OOH	KRO2HO2	KRO2HO2
120	R19OO + NO = R19O + NO2	KRO2NO*0.9	KRO2NO*0.9
121	R19OO + NO = R19ONO2	KRO2NO*0.1	KRO2NO*0.1
122	R21OO + HO2 = R21OOH	KRO2HO2	KRO2HO2
123	R21OO + NO = R21O + NO2	KRO2NO*0.9	KRO2NO*0.9
124	R21OO + NO = R21ONO2	KRO2NO*0.1	KRO2NO*0.1
125	R22OO + HO2 = R22OOH	KRO2HO2	KRO2HO2
126	R22OO + NO = R22O + NO2	KRO2NO*0.9	KRO2NO*0.9
127	R22OO + NO = R22ONO2	KRO2NO*0.1	KRO2NO*0.1
128	R23OO + HO2 = R23OOH	KRO2HO2	KRO2HO2
129	R23OO + NO = R23O + NO2	KRO2NO*0.9	KRO2NO*0.9
130	R23OO + NO = R23ONO2	KRO2NO*0.1	KRO2NO*0.1
131	R25OO + HO2 = R25OOH	KRO2HO2	KRO2HO2
132	R25OO + NO = R25O + NO2	KRO2NO*0.9	KRO2NO*0.9
133	R25OO + NO = R25ONO2	KRO2NO*0.1	KRO2NO*0.1
134	R26OO + HO2 = R26OOH	KRO2HO2	KRO2HO2
135	R26OO + NO = R26O + NO2	KRO2NO*0.9	KRO2NO*0.9
136	R26OO + NO = R26ONO2	KRO2NO*0.1	KRO2NO*0.1
137	R27OO + HO2 = R27OOH	KRO2HO2	KRO2HO2
138	R27OO + NO = R27O + NO2	KRO2NO*0.9	KRO2NO*0.9
139	R27OO + NO = R27ONO2	KRO2NO*0.1	KRO2NO*0.1

Table S4. OH addition mechanism added to α-pinene + OH subset of MCM. Incorporated reactions and rate coefficients (following Xu *et al.* (*27*) in the default α-pinene + OH subset of MCM mechanism.

No.	Reactions	Rate coefficients (1st order reactions: s^{-1} 2nd order reactions: cm^3 $molecule^{-1}$ s^{-1})
1	APINENE + OH = anti-APINCO2	1.2E-11*EXP(440/TEMP)*0.91*0.075*0.5
2	APINENE + OH = syn-APINCO2	1.2E-11*EXP(440/TEMP)*0.91*0.075*0.5
3	APINCOOH + OH = anti-APINCO2	1.03E-10*0.5
4	APINCOOH + OH = syn-APINCO2	1.03E-10*0.5
5	anti-APINCO2 + HO2 = APINCOOH	KRO2HO2*0.914
6	anti-APINCO2 + NO = APINCNO3	KRO2NO*0.125
7	anti-APINCO2 + NO = APINCO + NO2	KRO2NO*0.875
8	anti-APINCO2 + NO3 = APINCO + NO2	KRO2NO3
9	anti-APINCO2 = APINCO	6.70E-15*RO2*0.7
10	anti-APINCO2 = APINCOH	6.70E-15*RO2*0.3
11	syn-APINCO2 + HO2 = APINCOOH	KRO2HO2*0.914
12	syn-APINCO2 + NO = APINCNO3	KRO2NO*0.125
13	syn-APINCO2 + NO = APINCO + NO2	KRO2NO*0.875
14	syn-APINCO2 + NO3 = APINCO + NO2	KRO2NO3
15	syn-APINCO2 = APINCO	6.70E-15*RO2*0.7
16	syn-APINCO2 = APINCOH	6.70E-15*RO2*0.3
17	anti-APINCO2 = anti-R4	1.1*0.5
18	anti-R4 + NO = P4	KRO2NO*0.1
19	anti-R4 + NO = R7	KRO2NO*0.9
20	anti-R4 + HO2 = R4-ROOH	KRO2HO2
21	anti-R4 = R4-C10H17O5-RO2	1.0E+02
22	R4-C10H17O5-RO2 = R4-C10H16O5	0.1
23	R4-C10H17O5-RO2 + NO = R4-C10H17O5-RONO2	KRO2NO*0.1
24	R4-C10H17O5-RO2 + NO = R4-C10H17O5-RO	KRO2NO*0.9
25	R4-C10H17O5-RO2 + HO2 = R4-C10H17O5-ROOH	KRO2HO2
26	R4-C10H17O5-RO2 = R4-C10H17O7-RO2-endo-A	1.0E+00
27	R4-C10H17O7-RO2-endo-A + NO = R4-C10H17O7-RONO2-endo-A	KRO2NO*0.1
28	R4-C10H17O7-RO2-endo-A + NO = R4-C10H17O7-RO-endo-A + NO2	KRO2NO*0.9
29	R4-C10H17O7-RO2-endo-A + HO2 = R4-C10H17O7-ROOH-endo-A	KRO2HO2
30	R4-C10H17O7-RO2-endo-A = R4-C10H17O7-RO2-endo-B	1.0E+02
31	R4-C10H17O7-RO2-endo-B + NO = R4-C10H17O7-RONO2-endo-B	KRO2NO*0.1
32	R4-C10H17O7-RO2-endo-B + NO = R4-C10H17O7-RO-endo-B + NO2	KRO2NO*0.9
33	R4-C10H17O7-RO2-endo-B + HO2 = R4-C10H17O7-ROOH-endo-B	KRO2HO2
34	R4-C10H17O5-RO2 = R13	2.96E+01*0.5
35	R4-C10H17O5-RO2 = R4-C10H17O7-RO2	2.96E+01*0.5

36	R4-C10H17O7-RO2 + NO = R4-C10H17O7-RONO2	KRO2NO*0.1
37	R4-C10H17O7-RO2 + NO = R4-C10H17O7-RO + NO2	KRO2NO*0.9
38	R4-C10H17O7-RO2 + HO2 = R4-C10H17O7-ROOH	KRO2HO2
39	R4-C10H17O7-RO2 = R4-C10H17O9-RO2-endo	1.0E+00
40	R4-C10H17O9-RO2-endo + NO = R4-C10H17O9-RONO2-endo	KRO2NO*0.1
41	R4-C10H17O9-RO2-endo + NO = R4-C10H17O9-RO-endo + NO2	KRO2NO*0.9
42	R4-C10H17O9-RO2-endo + HO2 = R4-C10H17O9-ROOH-endo	KRO2HO2
43	anti-R4 = R11	1.0E+05
44	R11 + NO = R11-RONO2	KRO2NO*0.1
45	R11 + NO = R11-RO	KRO2NO*0.9
46	R11 + HO2 = R11-ROOH	KRO2HO2
47	R11 = R12	1.00E-01
48	R12 = R13	1.00E+06
49	R13 + NO = R13-RONO2	KRO2NO*0.1
50	R13 + NO = R13-RO	KRO2NO*0.9
51	R13 + HO2 = R13-ROOH	KRO2HO2
52	R13 = R14	1.00E+03
53	R14 + NO = R14-RONO2	KRO2NO*0.1
54	R14 + NO = R14-RO	KRO2NO*0.9
55	R14 + HO2 = R14-ROOH	KRO2HO2
56	R14 = R15	1.00E-01
57	R15 = R16	1.00E+06
58	R16 + NO = R16-RONO2	KRO2NO*0.1
59	R16 + NO = R16-RO	KRO2NO*0.9
60	R16 + HO2 = R16-ROOH	KRO2HO2
61	R16 = R17	1.00E+06
62	R17 = R18	1.00E+06
63	R18 + NO = R18-RONO2	KRO2NO*0.1
64	R18 + NO = R18-RO	KRO2NO*0.9
65	R18 + HO2 = R18-ROOH	KRO2HO2
66	R18 = R19	1.00E+05
67	R19 + NO = R19-RONO2	KRO2NO*0.1
68	R19 + NO = R19-RO	KRO2NO*0.9
69	R19 + HO2 = R19-ROOH	KRO2HO2
70	R19 = C10H16O10	1.00E-01
71	R7 = R7-C10H17O4-RO2	1.00E+05
72	R7 = R7-C10H17O4-endo	1.00E+00
73	R7 = R7-C10H17O6-RO2	KDEC
74	R7-C10H17O6-RO2 + HO2 = R7-C10H17O6-ROOH	KRO2HO2
75	R7-C10H17O6-RO2 + NO = R7-C10H17O6-RONO2	KRO2NO*0.1
76	R7-C10H17O6-RO2 + NO = R7-C10H17O6-RO	KRO2NO*0.9

77	R7-C10H17O6-RO = R7-C10H17O7-RO2	5.00E-01
78	R7-C10H17O7-RO2 + NO = R7-C10H17O7-RONO2	KRO2NO*0.1
79	R7-C10H17O7-RO2 + NO = R7-C10H17O7-RO	KRO2NO*0.9
80	R7-C10H17O7-RO2 + HO2 = R7-C10H17O7-ROOH	KRO2HO2
81	R7-C10H17O7-RO2 = R7-C10H16O7	1.00E-01
82	R7-C10H17O6-RO = R7-C7 + ACETONE	KDEC
83	R7-C10H17O6-RO = R7-C10H17O5-RO2	1.00E+05
84	R7-C10H17O5-RO2 + NO = R7-C10H17O5-RONO2	KRO2NO*0.1
85	R7-C10H17O5-RO2 + NO = R7-C10H17O5-RO	KRO2NO*0.9
86	R7-C10H17O5-RO2 + HO2 = R7-C10H17O5-ROOH	KRO2HO2
87	R7-C10H17O6-RO2 = R7-C10H17O8-RO2	5.00E-01
88	R7-C10H17O8-RO2 + NO = R7-C10H17O8-RONO2	KRO2NO*0.1
89	R7-C10H17O8-RO2 + NO = R7-C10H17O8-RO + NO2	KRO2NO*0.9
90	R7-C10H17O8-RO2 + HO2 = R7-C10H17O8-ROOH	KRO2HO2
91	R7-C10H17O8-RO2 = R7-C10H16O8	1.00E-01
92	R7-C10H17O8-RO2 = R7-C10H17O10-RO2	3.96E+00
93	R7-C10H17O10-RO2 + NO = R7-C10H17O10-RONO2	KRO2NO*0.1
94	R7-C10H17O10-RO2 + NO = R7-C10H17O10-RO +NO2	KRO2NO*0.9
95	R7-C10H17O10-RO2 + HO2 = R7-C10H17O10-ROOH	KRO2HO2
96	R7-C10H17O6-RO2 = R7-C10H17O6-RO2-A	1.00E+02
97	R7-C10H17O6-RO2-A + HO2 = R7-C10H17O6-ROOH-A	KRO2HO2
98	R7-C10H17O6-RO2-A + NO = R7-C10H17O6-RONO2-A	KRO2NO*0.1
99	R7-C10H17O6-RO2-A + NO = R7-C10H17O6-RO-A	KRO2NO*0.9
100	anti-APINCO2 = anti-R5	1.1E+00*0.5
101	anti-R5 + NO = P5	KRO2NO*0.1
102	anti-R5 + NO = R8	KRO2NO*0.9
103	anti-R5 + HO2 = R5-ROOH	KRO2HO2
104	anti_R5 = R5-C10H17O5-RO2	1.00E+02
105	R5-C10H17O5-RO2 + NO = R5-C10H17O5-RONO2	KRO2NO*0.1
106	R5-C10H17O5-RO2 + NO = R5-C10H17O5-RO + NO2	KRO2NO*0.9
107	R5-C10H17O5-RO2 + HO2 = R5-C10H17O5-ROOH	KRO2HO2
108	R5-C10H17O5-RO2 = R5-C10H16O5	1.00E-01
109	R5-C10H17O5-RO2 = R5-C10H17O7-RO2-endo	1.00E+00
110	R5-C10H17O7-RO2-endo + NO = R5-C10H17O7-RONO2-endo	KRO2NO*0.1
111	R5-C10H17O7-RO2-endo + NO = R5-C10H17O7-RO-endo + NO2	KRO2NO*0.9
112	R5-C10H17O7-RO2-endo + HO2 = R5-C10H17O7-ROOH-endo	KRO2HO2
113	anti-APINCO2 = P7	3.7E-01*0.5
114	anti-APINCO2 = R6	3.7E-01*0.5
115	R6 + NO = P6	KRO2NO*0.1
116	R6 + NO = R9	KRO2NO*0.9
117	R6 + HO2 = R6-ROOH	KRO2HO2

118	R6 = R6-C10H17O5-RO2	1.00E+02
119	R6-C10H17O5-RO2 + NO = R6-C10H17O5-RONO2	KRO2NO*0.1
120	R6-C10H17O5-RO2 + NO = R6-C10H17O5-RO + NO2	KRO2NO*0.9
121	R6-C10H17O5-RO2 + HO2 = R6-C10H17O5-ROOH	KRO2HO2
122	R6-C10H17O5-RO2 = R6-C10H16O4	1.00E-01
123	R6-C10H17O5-RO2 = R6-C10H17O7-RO2-endo	1.00E+00
124	R6-C10H17O7-RO2-endo + NO = R6-C10H17O7-RONO2-endo	KRO2NO*0.1
125	R6-C10H17O7-RO2-endo + NO = R6-C10H17O7-RO-endo + NO2	KRO2NO*0.9
126	R6-C10H17O7-RO2-endo + HO2 = R6-C10H17O7-ROOH-endo	KRO2HO2
127	anti-APINCO2 = R1	3.50E-01
128	R1 + NO = P1	KRO2NO*0.1
129	R1 + NO = R2	KRO2NO*0.9
130	R1 + HO2 = R1-ROOH	KRO2HO2
131	anti-APINCO2 = C10H17O3-endo	2.00E-03
132	syn-APINCO2 = R1	2.30E+00
133	syn-APINCO2 = C10H17O3_endo	2.60E-02
134	syn-APINCO2 = syn-R4	1.6E-1*0.5
135	syn-R4 + NO = P4	KRO2NO*0.1
136	syn-R4 + NO = R7 + NO2	KRO2NO*0.9
137	syn-R4 + HO2 = R4-ROOH	KRO2HO2
138	syn-R4 = R4-C10H17O5-RO2	1.00E+02
138	syn-APINCO2 = syn-R5	1.6E-1*0.5
138	syn-R5 + NO = P5	KRO2NO*0.1
138	syn-R5 + NO = R8 + NO2	KRO2NO*0.9
138	syn-R5 + HO2 = R5-ROOH	KRO2HO2
138	syn_R5 = R5-C10H17O5-RO2	1.00E+02

Table S5. Reported products and their molar yields from α-pinene + OH and α-pinene + O_3 using different ionizations. Note that molar yields that are not from direct measurements are marked with asterisks (*).

Reaction	Molar yield	Products	Ionization
α-pinene + OH	≤0.01 (*11*)*		Nitrate
	0.0044 (-50%/+100%) (*21*)*		Nitrate
	0.012 (-60%/+100%) (*12*)	Selected products: $C_{8\text{-}10}H_{14\text{-}16}O_{6\text{-}12}$ $C_{19\text{-}20}H_{28\text{-}30}O_{9\text{-}14}$	Nitrate
	0.00052 (-50%/+100%) (*26*)	Most abundant product: HO-$C_{10}H_{15}$(OO)(OOH)O_2	Nitrate
	0.024 (-50%/+100%) (*26*)	Most abundant product: HO-$C_{10}H_{15}$(OO)(OOH)O_2	Acetate
	0.75-0.81 (-58%/+104%) (*25*)	HO-$C_{10}H_{16}O_{2\text{-}6}$•	Iodide Ethylaminium
	0.42-0.45 (-58%/+104%) (*25*)	$C_{10}H_{16}O_2$•	Iodide Ethylaminium
	0.17-0.19 (-58%/+104%) (*25*)	$C_{10}H_{16}O_4$•	Iodide Ethylaminium
	0.076 (-50%/+100%) (*65*)	HO-$C_{10}H_{16}(O_2)_XO_2$• (X=2)	Methylaminium
	0.076 (-50%/+100%) (*65*)	HO-$C_{10}H_{16}(O_2)_XO_2$• (X=2)	Ethylaminium
	0.0484 (-50%/+100%) (*65*)	HO-$C_{10}H_{16}(O_2)_XO_2$• (X=2)	n-propylaminium
	0.008-0.011 (-55%/+144%) (this study)	Main products: $C_{10}H_{15}O_{10}$• $C_{10}H_{14}O_{10}$ $C_{10}H_{15}NO_{11}$	Nitrate
α-pinene + O_3	0.07 (±0.035) (*11*)	Example products: $C_{10}H_{14\text{-}16}O_{7\text{-}11}$ $C_{19\text{-}20}H_{28\text{-}32}O_{10\text{-}18}$	Nitrate
	0.034 (-50%/+100%) (*21*)	"Fingerprints" products: $C_{10}H_{14\text{-}16}O_{7\text{-}10}$ $C_{19\text{-}20}H_{28\text{-}32}O_{9\text{-}18}$	Nitrate
	0.029 (-60%/+100%) (*12*)	Selected products: $C_{8\text{-}10}H_{14\text{-}16}O_{6\text{-}12}$ $C_{19\text{-}20}H_{28\text{-}30}O_{9\text{-}14}$	Nitrate
	0.021 (-50%/+100%) (*26*)	O,O-$C_{10}H_{15}(O_2)_XO_2$• X=1 - 4	Nitrate
	0.024 (-50%/+100%) (*26*)	O,O-$C_{10}H_{15}(O_2)_XO_2$• X=1 - 4	Acetate
	0.024 (-50%/+100%) (*26*)	O,O-$C_{10}H_{15}(O_2)_XO_2$• X=1 - 4	Lactate
	0.034 (-50%/+100%) (*26*)	O,O-$C_{10}H_{15}(O_2)_XO_2$• X=1 - 4	Pyruvate
	0.055 (-50%/+100%) (*65*)	O,O-$C_{10}H_{15}(O_2)_XO_2$• (X=2, 3)	Methylaminium
	0.056 (-50%/+100%) (*65*)	O,O-$C_{10}H_{15}(O_2)_XO_2$• (X=2, 3)	Ethylaminium
	0.0365 (-50%/+100%) (*65*)	O,O-$C_{10}H_{15}(O_2)_XO_2$• (X=2, 3)	n-propylaminium

REFERENCES AND NOTES

1. Q. Zhang, J. L. Jimenez, M. R. Canagaratna, J. D. Allan, H. Coe, I. Ulbrich, M. R. Alfarra, A. Takami, A. M. Middlebrook, Y. L. Sun, K. Dzepina, E. Dunlea, K. Docherty, P. F. DeCarlo, D. Salcedo, T. Onasch, J. T. Jayne, T. Miyoshi, A. Shimono, S. Hatakeyama, N. Takegawa, Y. Kondo, J. Schneider, F. Drewnick, S. Borrmann, S. Weimer, K. Demerjian, P. Williams, K. Bower, R. Bahreini, L. Cottrell, R. J. Griffin, J. Rautiainen, J. Y. Sun, Y. M. Zhang, D. R. Worsnop, Ubiquity and dominance of oxygenated species in organic aerosols in anthropogenically-influenced Northern Hemisphere midlatitudes. *Geophys. Res. Lett.* **34**, L13801 (2007).

2. M. Kanakidou, J. H. Seinfeld, S. N. Pandis, I. Barnes, F. J. Dentener, M. C. Facchini, R. Van Dingenen, B. Ervens, A. Nenes, C. J. Nielsen, E. Swietlicki, J. P. Putaud, Y. Balkanski, S. Fuzzi, J. Horth, G. K. Moortgat, R. Winterhalter, C. E. L. Myhre, K. Tsigaridis, E. Vignati, E. G. Stephanou, J. Wilson, Organic aerosol and global climate modelling: A review. *Atmos. Chem. Phys.* **5**, 1053–1123 (2005).

3. J. L. Jimenez, M. R. Canagaratna, N. M. Donahue, A. S. H. Prevot, Q. Zhang, J. H. Kroll, P. F. DeCarlo, J. D. Allan, H. Coe, N. L. Ng, A. C. Aiken, K. S. Docherty, I. M. Ulbrich, A. P. Grieshop, A. L. Robinson, J. Duplissy, J. D. Smith, K. R. Wilson, V. A. Lanz, C. Hueglin, Y. L. Sun, J. Tian, A. Laaksonen, T. Raatikainen, J. Rautiainen, P. Vaattovaara, M. Ehn, M. Kulmala, J. M. Tomlinson, D. R. Collins, M. J. Cubison, E. J. Dunlea, J. A. Huffman, T. B. Onasch, M. R. Alfarra, P. I. Williams, K. Bower, Y. Kondo, J. Schneider, F. Drewnick, S. Borrmann, S. Weimer, K. Demerjian, D. Salcedo, L. Cottrell, R. Griffin, A. Takami, T. Miyoshi, S. Hatakeyama, A. Shimono, J. Y. Sun, Y. M. Zhang, K. Dzepina, J. R. Kimmel, D. Sueper, J. T. Jayne, S. C. Herndon, A. M. Trimborn, L. R. Williams, E. C. Wood, A. M. Middlebrook, C. E. Kolb, U. Baltensperger, D. R. Worsnop, Evolution of organic aerosols in the atmosphere. *Science* **326**, 1525–1529 (2009).

4. M. O. Andreae, D. Rosenfeld, Aerosol-cloud-precipitation interactions. Part 1. The nature and sources of cloud-active aerosols. *Earth. Sci. Rev.* **89**, 13–41 (2008).

5. T. M. VanReken, N. L. Ng, R. C. Flagan, J. H. Seinfeld, Cloud condensation nucleus activation properties of biogenic secondary organic aerosol. *J. Geophys. Res. Atmos.* **110**, D07206 (2005).

6. D. F. Zhao, A. Buchholz, R. Tillmann, E. Kleist, C. Wu, F. Rubach, A. Kiendler-Scharr, Y. Rudich, J. Wildt, T. F. Mentel, Environmental conditions regulate the impact of plants on cloud formation. *Nat. Commun.* **8**, 14067 (2017).

7. K. E. H. Hartz, T. Rosenorn, S. R. Ferchak, T. M. Raymond, M. Bilde, N. M. Donahue, S. N. Pandis, Cloud condensation nuclei activation of monoterpene and sesquiterpene secondary organic aerosol. *J. Geophys. Res. Atmos.* **110**, D14208 (2005).

8. K. Tsigaridis, M. Kanakidou, The present and future of secondary organic aerosol direct forcing on climate. *Curr. Clim. Change. Rep.* **4**, 84–98 (2018).

9. C. E. Scott, A. Rap, D. V. Spracklen, P. M. Forster, K. S. Carslaw, G. W. Mann, K. J. Pringle, N. Kivekas, M. Kulmala, H. Lihavainen, P. Tunved, The direct and indirect radiative effects of biogenic secondary organic aerosol. *Atmos. Chem. Phys.* **14**, 447–470 (2014).

10. M. Hallquist, J. C. Wenger, U. Baltensperger, Y. Rudich, D. Simpson, M. Claeys, J. Dommen, N. M. Donahue, C. George, A. H. Goldstein, J. F. Hamilton, H. Herrmann, T. Hoffmann, Y. Iinuma, M. Jang, M. E. Jenkin, J. L. Jimenez, A. Kiendler-Scharr, W. Maenhaut, G. McFiggans, T. F. Mentel, A. Monod, A. S. H. Prevot, J. H. Seinfeld, J. D. Surratt, R. Szmigielski, J. Wildt, The formation, properties and impact of secondary organic aerosol: Current and emerging issues. *Atmos. Chem. Phys.* **9**, 5155–5236 (2009).

11. M. Ehn, J. A. Thornton, E. Kleist, M. Sipilä, H. Junninen, I. Pullinen, M. Springer, F. Rubach, R. Tillmann, B. Lee, F. Lopez-Hilfiker, S. Andres, I. H. Acir, M. Rissanen, T. Jokinen, S. Schobesberger, J. Kangasluoma, J. Kontkanen, T. Nieminen, T. Kurtén, L. B. Nielsen, S. Jørgensen, H. G. Kjaergaard, M. Canagaratna, M. D. Maso, T. Berndt, T. Petäjä, A. Wahner, V. M. Kerminen, M. Kulmala, D. R. Worsnop, J. Wildt, T. F. Mentel, A large source of low-volatility secondary organic aerosol. *Nature* **506**, 476–479 (2014).

12. J. Kirkby, J. Duplissy, K. Sengupta, C. Frege, H. Gordon, C. Williamson, M. Heinritzi, M. Simon, C. Yan, J. Almeida, J. Trostl, T. Nieminen, I. K. Ortega, R. Wagner, A. Adamov, A. Amorim, A. K. Bernhammer, F. Bianchi, M. Breitenlechner, S. Brilke, X. M. Chen, J. Craven, A. Dias, S. Ehrhart, R. C. Flagan, A. Franchin, C. Fuchs, R. Guida, J. Hakala, C. R. Hoyle, T. Jokinen, H. Junninen, J. Kangasluoma, J. Kim, M. Krapf, A. Kurten, A. Laaksonen, K. Lehtipalo, V. Makhmutov, S. Mathot, U. Molteni, A. Onnela, O. Peräkylä, F. Piel, T. Petäjä, A. P. Praplan, K. Pringle, A. Rap, N. A. D. Richards, I. Riipinen, M. P. Rissanen, L. Rondo, N. Sarnela, S. Schobesberger, C. E. Scott, J. H. Seinfeld, M. Sipila, G. Steiner, Y. Stozhkov, F. Stratmann, A. Tome, A. Virtanen, A. L. Vogel, A. C. Wagner, P. E. Wagner, E. Weingartner, D. Wimmer, P. M. Winkler, P. L. Ye, X. Zhang, A. Hansel, J. Dommen, N. M. Donahue, D. R. Worsnop, U. Baltensperger, M. Kulmala, K. S. Carslaw, J. Curtius, Ion-induced nucleation of pure biogenic particles. *Nature* **533**, 521–526 (2016).

13. J. Tröstl, W. K. Chuang, H. Gordon, M. Heinritzi, C. Yan, U. Molteni, L. Ahlm, C. Frege, F. Bianchi, R. Wagner, M. Simon, K. Lehtipalo, C. Williamson, J. S. Craven, J. Duplissy, A. Adamov, J. Almeida, A. K. Bernhammer, M. Breitenlechner, S. Brilke, A. Dias, S. Ehrhart, R. C. Flagan, A. Franchin, C. Fuchs, R. Guida, M. Gysel, A. Hansel, C. R. Hoyle, T. Jokinen, H. Junninen, J. Kangasluoma, H. Keskinen, J. Kim, M. Krapf, A. Kurten, A. Laaksonen, M. Lawler, M. Leiminger, S. Mathot, O. Möhler, T. Nieminen, A. Onnela, T. Petäjä, F. M. Piel, P. Miettinen, M. P. Rissanen, L. Rondo, N. Sarnela, S. Schobesberger, K. Sengupta, M. Sipilä, J. N. Smith, G. Steiner, A. Tomè, A. Virtanen, A. C. Wagner, E. Weingartner, D. Wimmer, P. M. Winkler, P. L. Ye, K. S. Carslaw, J. Curtius, J. Dommen, J. Kirkby, M. Kulmala, I. Riipinen, D. R. Worsnop, N. M. Donahue, U. Baltensperger, The role of low-volatility organic compounds in initial particle growth in the atmosphere. *Nature* **533**, 527–531 (2016).

14. C. Mohr, J. A. Thornton, A. Heitto, F. D. Lopez-Hilfiker, A. Lutz, I. Riipinen, J. Hong, N. M. Donahue, M. Hallquist, T. Petäjä, M. Kulmala, T. Yli-Juuti, Molecular identification of organic vapors driving atmospheric nanoparticle growth. *Nat. Commun.* **10**, 4442 (2019).

15. G. McFiggans, T. F. Mentel, J. Wildt, I. Pullinen, S. Kang, E. Kleist, S. Schmitt, M. Springer, R. Tillmann, C. Wu, D. F. Zhao, M. Hallquist, C. Faxon, M. Le Breton, A. M. Hallquist, D. Simpson, R. Bergstrom, M. E. Jenkin, M. Ehn, J. A. Thornton, M. R. Alfarra, T. J. Bannan, C.

J. Percival, M. Priestley, D. Topping, A. Kiendler-Scharr, Secondary organic aerosol reduced by mixture of atmospheric vapours. *Nature* **565**, 587–593 (2019).

16. P. Roldin, M. Ehn, T. Kurtén, T. Olenius, M. P. Rissanen, N. Sarnela, J. Elm, P. Rantala, L. Q. Hao, N. Hyttinen, L. Heikkinen, D. R. Worsnop, L. Pichelstorfer, C. Xavier, P. Clusius, E. Öström, T. Petäjä, M. Kulmala, H. Vehkamäki, A. Virtanen, I. Riipinen, M. Boy, The role of highly oxygenated organic molecules in the boreal aerosol-cloud-climate system. *Nat. Commun.* **10**, 4370 (2019).

17. C. Rose, Q. Zha, L. Dada, C. Yan, K. Lehtipalo, H. Junninen, S. B. Mazon, T. Jokinen, N. Sarnela, M. Sipilä, T. Petäjä, V.-M. Kerminen, F. Bianchi, M. Kulmala, Observations of biogenic ion-induced cluster formation in the atmosphere. *Sci. Adv.* **4**, eaar5218 (2018).

18. F. Bianchi, T. Kurtén, M. Riva, C. Mohr, M. P. Rissanen, P. Roldin, T. Berndt, J. D. Crounse, P. O. Wennberg, T. F. Mentel, J. Wildt, H. Junninen, T. Jokinen, M. Kulmala, D. R. Worsnop, J. A. Thornton, N. Donahue, H. G. Kjaergaard, M. Ehn, Highly oxygenated organic molecules (HOM) from gas-phase autoxidation involving peroxy radicals: A key contributor to atmospheric aerosol. *Chem. Rev.* **119**, 3472–3509 (2019).

19. J. D. Crounse, L. B. Nielsen, S. Jørgensen, H. G. Kjaergaard, P. O. Wennberg, Autoxidation of organic compounds in the atmosphere. *J. Phys. Chem. Lett.* **4**, 3513–3520 (2013).

20. S. Iyer, M. P. Rissanen, R. Valiev, S. Barua, J. E. Krechmer, J. Thornton, M. Ehn, T. Kurtén, Molecular mechanism for rapid autoxidation in α-pinene ozonolysis. *Nat. Commun.* **12**, 878 (2021).

21. T. Jokinen, T. Berndt, R. Makkonen, V. M. Kerminen, H. Junninen, P. Paasonen, F. Stratmann, H. Herrmann, A. B. Guenther, D. R. Worsnop, M. Kulmala, M. Ehn, M. Sipilä, Production of extremely low volatile organic compounds from biogenic emissions: Measured yields and atmospheric implications. *Proc. Natl. Acad. Sci. U.S.A.* **112**, 7123–7128 (2015).

22. S. Tomaz, D. Wang, N. Zabalegui, D. Li, H. Lamkaddam, F. Bachmeier, A. Vogel, M. E. Monge, S. Perrier, U. Baltensperger, C. George, M. Rissanen, M. Ehn, I. El Haddad, M. Riva,

Structures and reactivity of peroxy radicals and dimeric products revealed by online tandem mass spectrometry. *Nat. Commun.* **12**, 300 (2021).

23. U. Molteni, M. Simon, M. Heinritzi, C. R. Hoyle, A. K. Bernhammer, F. Bianchi, M. Breitenlechner, S. Brilke, A. Dias, J. Duplissy, C. Frege, H. Gordon, C. Heyn, T. Jokinen, A. Kurten, K. Lehtipalo, V. Makhmutov, T. Petäjä, S. M. Pieber, A. P. Praplan, S. Schobesberger, G. Steiner, Y. Stozhkov, A. Tomé, J. Tröstl, A. C. Wagner, R. Wagner, C. Williamson, C. Yan, U. Baltensperger, J. Curtius, N. M. Donahue, A. Hansel, J. Kirkby, M. Kulmala, D. R. Worsnop, J. Dommen, Formation of highly oxygenated organic molecules from α-pinene ozonolysis: Chemical characteristics, mechanism, and kinetic model development. *ACS Earth Space Chem.* **3**, 873–883 (2019).

24. A. B. Guenther, X. Jiang, C. L. Heald, T. Sakulyanontvittaya, T. Duhl, L. K. Emmons, X. Wang, The model of emissions of gases and aerosols from nature version 2.1 (MEGAN2.1): An extended and updated framework for modeling biogenic emissions. *Geosci. Model Dev.* **5**, 1471–1492 (2012).

25. T. Berndt, Peroxy radical processes and product formation in the OH radical-initiated oxidation of α-pinene for near-atmospheric conditions. *J. Phys. Chem. A* **125**, 9151–9160 (2021).

26. T. Berndt, S. Richters, T. Jokinen, N. Hyttinen, T. Kurtén, R. V. Otkjær, H. G. Kjaergaard, F. Stratmann, H. Herrmann, M. Sipilä, M. Kulmala, M. Ehn, Hydroxyl radical-induced formation of highly oxidized organic compounds. *Nat. Commun.* **7**, 13677 (2016).

27. L. Xu, K. H. Møller, J. D. Crounse, R. V. Otkjær, H. G. Kjaergaard, P. O. Wennberg, Unimolecular reactions of peroxy radicals formed in the oxidation of α-pinene and β-pinene by hydroxyl radicals. *J. Phys. Chem. A* **123**, 1661–1674 (2019).

28. J. Peeters, L. Vereecken, G. Fantechi, The detailed mechanism of the OH-initiated atmospheric oxidation of α-pinene: A theoretical study. *Phys. Chem. Chem. Phys.* **3**, 5489–5504 (2001).

29. L. Vereecken, J. Peeters, Theoretical study of the formation of acetone in the OH-initiated atmospheric oxidation of α-pinene. *J. Phys. Chem. A* **104**, 11140–11146 (2000).

30. L. Vereecken, J. F. Müller, J. Peeters, Low-volatility poly-oxygenates in the OH-initiated atmospheric oxidation of alpha-pinene: Impact of non-traditional peroxyl radical chemistry. *Phys. Chem. Chem. Phys.* **9**, 5241–5248 (2007).

31. E. Praske, R. V. Otkjaer, J. D. Crounse, J. C. Hethcox, B. M. Stoltz, H. G. Kjaergaard, P. O. Wennberg, Atmospheric autoxidation is increasingly important in urban and suburban North America. *Proc. Natl. Acad. Sci. U.S.A.* **115**, 64–69 (2018).

32. J. J. Orlando, G. S. Tyndall, Laboratory studies of organic peroxy radical chemistry: An overview with emphasis on recent issues of atmospheric significance. *Chem. Soc. Rev.* **41**, 6294–6317 (2012).

33. J. Peeters, S. Vandenberk, E. Piessens, V. Pultau, H-atom abstraction in reactions of cyclic polyalkenes with OH. *Chemosphere* **38**, 1189–1195 (1999).

34. L. Vereecken, J. Peeters, Enhanced H-atom abstraction from pinonaldehyde, pinonic acid, pinic acid, and related compounds: Theoretical study of C–H bond strengths. *Phys. Chem. Chem. Phys.* **4**, 467–472 (2002).

35. C. Rio, P. M. Flaud, J. C. Loison, E. Villenave, Experimental revaluation of the importance of the abstraction channel in the reactions of monoterpenes with OH radicals. *Chem. Phys. Chem.* **11**, 3962–3970 (2010).

36. L. Vereecken, J. Peeters, H-atom abstraction by OH-radicals from (biogenic) (poly)alkenes: C-H bond strengths and abstraction rates. *Chem. Phys. Lett.* **333**, 162–168 (2001).

37. T. F. Mentel, M. Springer, M. Ehn, E. Kleist, I. Pullinen, T. Kurten, M. Rissanen, A. Wahner, J. Wildt, Formation of highly oxidized multifunctional compounds: Autoxidation of peroxy radicals formed in the ozonolysis of alkenes—Deduced from structure-product relationships. *Atmos. Chem. Phys.* **15**, 6745–6765 (2015).

38. D. F. Zhao, S. H. Schmitt, M. J. Wang, I. H. Acir, R. Tillmann, Z. F. Tan, A. Novelli, H. Fuchs, I. Pullinen, R. Wegener, F. Rohrer, J. Wildt, A. Kiendler-Scharr, A. Wahner, T. F. Mentel, Effects of NO_x and SO_2 on the secondary organic aerosol formation from photooxidation of α-pinene and limonene. *Atmos. Chem. Phys.* **18**, 1611–1628 (2018).

39. N. Hyttinen, O. Kupiainen-Määttä, M. P. Rissanen, M. Muuronen, M. Ehn, T. Kurtén, Modeling the charging of highly oxidized cyclohexene ozonolysis products using nitrate-based chemical ionization. *J. Phys. Chem. A* **119**, 6339–6345 (2015).

40. N. Hyttinen, M. P. Rissanen, T. Kurtén, Computational comparison of acetate and nitrate chemical ionization of highly oxidized cyclohexene ozonolysis intermediates and products. *J. Phys. Chem. A* **121**, 2172–2179 (2017).

41. I. Pullinen, S. Schmitt, S. Kang, M. Sarrafzadeh, P. Schlag, S. Andres, E. Kleist, T. F. Mentel, F. Rohrer, M. Springer, R. Tillmann, J. Wildt, C. Wu, D. F. Zhao, A. Wahner, A. Kiendler-Scharr, Impact of NO_x on secondary organic aerosol (SOA) formation from α-pinene and β-pinene photooxidation: The role of highly oxygenated organic nitrates. *Atmos. Chem. Phys.* **20**, 10125–10147 (2020).

42. T. Berndt, W. Scholz, B. Mentler, L. Fischer, H. Herrmann, M. Kulmala, A. Hansel, Accretion product formation from self- and cross-reactions of RO_2 radicals in the atmosphere. *Angew. Chem. Int. Edit.* **57**, 3820–3824 (2018).

43. Y. Zhao, J. A. Thornton, H. O. T. Pye, Quantitative constraints on autoxidation and dimer formation from direct probing of monoterpene-derived peroxy radical chemistry. *Proc. Natl. Acad. Sci. U.S.A.* **115**, 12142–12147 (2018).

44. H. Shen, D. Zhao, I. Pullinen, S. Kang, L. Vereecken, H. Fuchs, I.-H. Acir, R. Tillmann, F. Rohrer, J. Wildt, A. Kiendler-Scharr, A. Wahner, T. F. Mentel, Highly oxygenated organic nitrates formed from NO_3 radical-initiated oxidation of β-pinene. *Environ. Sci. Technol.* **55**, 15658–15671 (2021).

45. J. Wildt, T. F. Mentel, A. Kiendler-Scharr, T. Hoffmann, S. Andres, M. Ehn, E. Kleist, P. Musgen, F. Rohrer, Y. Rudich, M. Springer, R. Tillmann, A. Wahner, Suppression of new particle formation from monoterpene oxidation by NO_x. *Atmos. Chem. Phys.* **14**, 2789–2804 (2014).

46. C. Yan, W. Nie, A. L. Vogel, L. Dada, K. Lehtipalo, D. Stolzenburg, R. Wagner, M. P. Rissanen, M. Xiao, L. Ahonen, L. Fischer, C. Rose, F. Bianchi, H. Gordon, M. Simon, M. Heinritzi, O. Garmash, P. Roldin, A. Dias, P. Ye, V. Hofbauer, A. Amorim, P. S. Bauer, A. Bergen, A. K. Bernhammer, M. Breitenlechner, S. Brilke, A. Buchholz, S. B. Mazon, M. R. Canagaratna, X. Chen, A. Ding, J. Dommen, D. C. Draper, J. Duplissy, C. Frege, C. Heyn, R. Guida, J. Hakala, L. Heikkinen, C. R. Hoyle, T. Jokinen, J. Kangasluoma, J. Kirkby, J. Kontkanen, A. Kurten, M. J. Lawler, H. Mai, S. Mathot, R. L. Mauldin, U. Molteni, L. Nichman, T. Nieminen, J. Nowak, A. Ojdanic, A. Onnela, A. Pajunoja, T. Petaja, F. Piel, L. L. J. Quelever, N. Sarnela, S. Schallhart, K. Sengupta, M. Sipila, A. Tome, J. Trostl, O. Vaisanen, A. C. Wagner, A. Ylisirnio, Q. Zha, U. Baltensperger, K. S. Carslaw, J. Curtius, R. C. Flagan, A. Hansel, I. Riipinen, J. N. Smith, A. Virtanen, P. M. Winkler, N. M. Donahue, V. M. Kerminen, M. Kulmala, M. Ehn, D. R. Worsnop, Size-dependent influence of NO_x on the growth rates of organic aerosol particles. *Sci. Adv.* **6**, eaay4945 (2020).

47. K. Lehtipalo, C. Yan, L. Dada, F. Bianchi, M. Xiao, R. Wagner, D. Stolzenburg, L. R. Ahonen, A. Amorim, A. Baccarini, P. S. Bauer, B. Baumgartner, A. Bergen, A.-K. Bernhammer, M. Breitenlechner, S. Brilke, A. Buchholz, S. B. Mazon, D. Chen, X. Chen, A. Dias, J. Dommen, D. C. Draper, J. Duplissy, M. Ehn, H. Finkenzeller, L. Fischer, C. Frege, C. Fuchs, O. Garmash, H. Gordon, J. Hakala, X. He, L. Heikkinen, M. Heinritzi, J. C. Helm, V. Hofbauer, C. R. Hoyle, T. Jokinen, J. Kangasluoma, V.-M. Kerminen, C. Kim, J. Kirkby, J. Kontkanen, A. Kürten, M. J. Lawler, H. Mai, S. Mathot, R. L. Mauldin, U. Molteni, L. Nichman, W. Nie, T. Nieminen, A. Ojdanic, A. Onnela, M. Passananti, T. Petäjä, F. Piel, V. Pospisilova, L. L. J. Quéléver, M. P. Rissanen, C. Rose, N. Sarnela, S. Schallhart, S. Schuchmann, K. Sengupta, M. Simon, M. Sipilä, C. Tauber, A. Tomé, J. Tröstl, O. Väisänen, A. L. Vogel, R. Volkamer, A. C. Wagner, M. Wang, L. Weitz, D. Wimmer, P. Ye, A. Ylisirniö, Q. Zha, K. S. Carslaw, J. Curtius, N. M. Donahue, R. C. Flagan, A. Hansel, I. Riipinen, A. Virtanen, P. M. Winkler, U. Baltensperger, M. Kulmala, D. R. Worsnop, Multicomponent new

particle formation from sulfuric acid, ammonia, and biogenic vapors. *Sci. Adv.* **4**, eaau5363 (2018).

48. S. M. Saunders, M. E. Jenkin, R. G. Derwent, M. J. Pilling, Protocol for the development of the Master Chemical Mechanism, MCM v3 (Part A): Tropospheric degradation of non-aromatic volatile organic compounds. *Atmos. Chem. Phys.* **3**, 161–180 (2003).

49. K. H. Møller, R. V. Otkjær, J. Chen, H. G. Kjaergaard, Double bonds are key to fast unimolecular reactivity in first-generation monoterpene hydroxy peroxy radicals. *J. Phys. Chem. A* **124**, 2885–2896 (2020).

50. L. Vereecken, B. Nozière, H migration in peroxy radicals under atmospheric conditions. *Atmos. Chem. Phys.* **20**, 7429–7458 (2020).

51. L. Vereecken, J. Peeters, Decomposition of substituted alkoxy radicals—Part I: A generalized structure-activity relationship for reaction barrier heights. *Phys. Chem. Chem. Phys.* **11**, 9062–9074 (2009).

52. F. Rohrer, B. Bohn, T. Brauers, D. Bruning, F. J. Johnen, A. Wahner, J. Kleffmann, Characterisation of the photolytic HONO-source in the atmosphere simulation chamber SAPHIR. *Atmos. Chem. Phys.* **5**, 2189–2201 (2005).

53. K. H. Møller, K. H. Bates, H. G. Kjaergaard, The importance of peroxy radical hydrogen-shift reactions in atmospheric isoprene oxidation. *J. Phys. Chem. A* **123**, 920–932 (2019).

54. S. Jørgensen, H. C. Knap, R. V. Otkjær, A. M. Jensen, M. L. H. Kjeldsen, P. O. Wennberg, H. G. Kjaergaard, Rapid hydrogen shift scrambling in hydroperoxy-substituted organic peroxy radicals. *J. Phys. Chem. A* **120**, 266–275 (2016).

55. E. Praske, R. V. Otkjær, J. D. Crounse, J. C. Hethcox, B. M. Stoltz, H. G. Kjaergaard, P. O. Wennberg, Intramolecular hydrogen shift chemistry of hydroperoxy-substituted peroxy radicals. *J. Phys. Chem. A* **123**, 590–600 (2019).

56. L. Vereecken, G. Vu, A. Wahner, A. Kiendler-Scharr, H. M. T. Nguyen, A structure activity relationship for ring closure reactions in unsaturated alkylperoxy radicals. *Phys. Chem. Chem. Phys.* **23**, 16564–16576 (2021).

57. S. Kang, "Formation of highly oxygenated organic molecules from α-pinene photochemistry", thesis, University of Wuppertal (2021).

58. T. Berndt, N. Hyttinen, H. Herrmann, A. Hansel, First oxidation products from the reaction of hydroxyl radicals with isoprene for pristine environmental conditions. *Commun. Chem.* **2**, 21 (2019).

59. M. Riva, P. Rantala, J. E. Krechmer, O. Peräkylä, Y. Zhang, L. Heikkinen, O. Garmash, C. Yan, M. Kulmala, D. Worsnop, M. Ehn, Evaluating the performance of five different chemical ionization techniques for detecting gaseous oxygenated organic species. *Atmos. Meas. Tech.* **12**, 2403–2421 (2019).

60. T. Jokinen, M. Sipila, H. Junninen, M. Ehn, G. Lonn, J. Hakala, T. Petaja, R. L. Mauldin, M. Kulmala, D. R. Worsnop, Atmospheric sulphuric acid and neutral cluster measurements using CI-APi-TOF. *Atmos. Chem. Phys.* **12**, 4117–4125 (2012).

61. F. L. Eisele, D. J. Tanner, Measurement of the gas phase concentration of H_2SO_4 and methane sulfonic acid and estimates of H_2SO_4 production and loss in the atmosphere. *J. Geophys. Res. Atmos.* **98**, 9001–9010 (1993).

62. S. M. Aschmann, R. Atkinson, Rate constants for the gas-phase reactions of alkanes with Cl atoms at 296 ± 2 K. *Int. J. Chem. Kinet.* **27**, 613–622 (1995).

63. C. G. Masoud, L. H. Ruiz, Chlorine-initiated oxidation of α-pinene: Formation of secondary organic aerosol and highly oxygenated organic molecules. *ACS Earth Space Chem.* **5**, 2307–2319 (2021).

64. Y. H. Wang, M. Riva, H. B. Xie, L. Heikkinen, S. Schallhart, Q. Z. Zha, C. Yan, X. C. He, O. Perakyla, M. Ehn, Formation of highly oxygenated organic molecules from chlorine-atom-initiated oxidation of alpha-pinene. *Atmos. Chem. Phys.* **20**, 5145–5155 (2020).

65. T. Berndt, K. H. Møller, H. Herrmann, H. G. Kjaergaard, Trimethylamine outruns terpenes and aromatics in atmospheric autoxidation. *J. Phys. Chem. A* **125**, 4454–4466 (2021).

66. C. Yan, W. Nie, M. Aijala, M. P. Rissanen, M. R. Canagaratna, P. Massoli, H. Junninen, T. Jokinen, N. Sarnela, S. A. K. Hame, S. Schobesberger, F. Canonaco, L. Yao, A. S. H. Prévôt, T. Petäjä, M. Kulmala, M. Sipilä, D. R. Worsnop, M. Ehn, Source characterization of highly oxidized multifunctional compounds in a boreal forest environment using positive matrix factorization. *Atmos. Chem. Phys.* **16**, 12715–12731 (2016).

67. T. Jokinen, J. Kontkanen, K. Lehtipalo, H. E. Manninen, J. Aalto, A. Porcar-Castell, O. Garmash, T. Nieminen, M. Ehn, J. Kangasluoma, H. Junninen, J. Levula, J. Duplissy, L. R. Ahonen, P. Rantala, L. Heikkinen, C. Yan, M. Sipilä, D. R. Worsnop, J. Bäck, T. Petäjä, V.-M. Kerminen, M. Kulmala, Solar eclipse demonstrating the importance of photochemistry in new particle formation. *Sci. Rep.* **7**, 45707 (2017).

68. P. Massoli, H. Stark, M. R. Canagaratna, J. E. Krechmer, L. Xu, N. L. Ng, R. L. Mauldin, C. Yan, J. Kimmel, P. K. Misztal, J. L. Jimenez, J. T. Jayne, D. R. Worsnop, Ambient measurements of highly oxidized gas-phase molecules during the Southern Oxidant and Aerosol Study (SOAS) 2013. *ACS Earth Space Chem.* **2**, 653–672 (2018).

69. H. O. T. Pye, E. L. D'Ambro, B. Lee, S. Schobesberger, M. Takeuchi, Y. Zhao, F. Lopez-Hilfiker, J. M. Liu, J. E. Shilling, J. Xing, R. Mathur, A. M. Middlebrook, J. Liao, A. Welti, M. Graus, C. Warneke, J. A. de Gouw, J. S. Holloway, T. B. Ryerson, I. B. Pollack, J. A. Thornton, Anthropogenic enhancements to production of highly oxygenated molecules from autoxidation. *Proc. Natl. Acad. Sci. U.S.A.* **116**, 6641–6646 (2019).

70. R. Atkinson, J. Arey, Atmospheric degradation of volatile organic compounds. *Chem. Rev.* **103**, 4605–4638 (2003).

71. A. Hofzumahaus, F. Rohrer, K. D. Lu, B. Bohn, T. Brauers, C. C. Chang, H. Fuchs, F. Holland, K. Kita, Y. Kondo, X. Li, S. R. Lou, M. Shao, L. M. Zeng, A. Wahner, Y. H. Zhang, Amplified trace gas removal in the troposphere. *Science* **324**, 1702–1704 (2009).

72. I. R. Piletic, T. E. Kleindienst, Rates and yields of unimolecular reactions producing highly oxidized peroxy radicals in the OH-induced autoxidation of α-pinene, β-pinene, and limonene. *J. Phys. Chem. A* **126**, 88–100 (2022).

73. Z. D. Wang, M. Ehn, M. P. Rissanen, O. Garmash, L. Quéléver, L. L. Xing, M. Monge-Palacios, P. Rantala, N. M. Donahue, T. Berndt, S. M. Sarathy, Efficient alkane oxidation under combustion engine and atmospheric conditions. *Commun. Chem.* **4**, 18 (2021).

74. E. L. D'Ambro, N. Hyttinen, K. H. Møller, S. Iyer, R. V. Otkjær, D. M. Bell, J. Liu, F. D. Lopez-Hilfiker, S. Schobesberger, J. E. Shilling, A. Zelenyuk, H. G. Kjaergaard, J. A. Thornton, T. Kurtén, Pathways to highly oxidized products in the Δ3-carene + OH system. *Environ. Sci. Technol.* **56**, 2213–2224 (2022).

75. B. Bohn, F. Rohrer, T. Brauers, A. Wahner, Actinometric measurements of NO_2 photolysis frequencies in the atmosphere simulation chamber SAPHIR. *Atmos. Chem. Phys.* **5**, 493–503 (2005).

76. D. F. Zhao, I. Pullinen, H. Fuchs, S. Schrade, R. R. Wu, I. H. Acir, R. Tillmann, F. Rohrer, J. Wildt, Y. D. Guo, A. Kiendler-Scharr, A. Wahner, S. Kang, L. Vereecken, T. F. Mentel, Highly oxygenated organic molecule (HOM) formation in the isoprene oxidation by NO_3 radical. *Atmos. Chem. Phys.* **21**, 9681–9704 (2021).

77. D. F. Zhao, M. Kaminski, P. Schlag, H. Fuchs, I. H. Acir, B. Bohn, R. Haseler, A. Kiendler-Scharr, F. Rohrer, R. Tillmann, M. J. Wang, R. Wegener, J. Wildt, A. Wahner, T. F. Mentel, Secondary organic aerosol formation from hydroxyl radical oxidation and ozonolysis of monoterpenes. *Atmos. Chem. Phys.* **15**, 991–1012 (2015).

78. M. P. Rissanen, T. Kurtén, M. Sipilä, J. A. Thornton, J. Kangasluoma, N. Sarnela, H. Junninen, S. Jørgensen, S. Schallhart, M. K. Kajos, R. Taipale, M. Springer, T. F. Mentel, T. Ruuskanen, T. Petäjä, D. R. Worsnop, H. G. Kjaergaard, M. Ehn, The formation of highly oxidized multifunctional products in the ozonolysis of cyclohexene. *J. Am. Chem. Soc.* **136**, 15596–15606 (2014).

79. H. Fuchs, H. P. Dorn, M. Bachner, B. Bohn, T. Brauers, S. Gomm, A. Hofzumahaus, F. Holland, S. Nehr, F. Rohrer, R. Tillmann, A. Wahner, Comparison of OH concentration measurements by DOAS and LIF during SAPHIR chamber experiments at high OH reactivity and low NO concentration. *Atmos. Meas. Tech.* **5**, 1611–1626 (2012).

80. R. Atkinson, D. L. Baulch, R. A. Cox, J. N. Crowley, R. F. Hampson, R. G. Hynes, M. E. Jenkin, M. J. Rossi, J. Troe, Evaluated kinetic and photochemical data for atmospheric chemistry: Volume I—Gas phase reactions of O_x, NO_x and SO_x species. *Atmos. Chem. Phys.* **4**, 1461–1738 (2004).

81. Y. L. Huang, R. Zhao, S. M. Charan, C. M. Kenseth, X. Zhang, J. H. Seinfeld, Unified theory of vapor-wall mass transport in Teflon-walled environmental chambers. *Environ. Sci. Technol.* **52**, 2134–2142 (2018).

82. P. J. Ziemann, R. Atkinson, Kinetics, products, and mechanisms of secondary organic aerosol formation. *Chem. Soc. Rev.* **41**, 6582–6605 (2012).

83. M. E. Jenkin, S. M. Saunders, M. J. Pilling, The tropospheric degradation of volatile organic compounds: A protocol for mechanism development. *Atmos. Environ.* **31**, 81–104 (1997).

84. N. C. Eddingsaas, C. L. Loza, L. D. Yee, J. H. Seinfeld, P. O. Wennberg, α-pinene photooxidation under controlled chemical conditions—Part 1: Gas-phase composition in low- and high-NO_x environments. *Atmos. Chem. Phys.* **12**, 6489–6504 (2012).

85. M. Rolletter, M. Kaminski, I. H. Acir, B. Bohn, H. P. Dorn, X. Li, A. Lutz, S. Nehr, F. Rohrer, R. Tillmann, R. Wegener, A. Hofzumahaus, A. Kiendler-Scharr, A. Wahner, H. Fuchs, Investigation of the α-pinene photooxidation by OH in the atmospheric simulation chamber SAPHIR. *Atmos. Chem. Phys.* **19**, 11635–11649 (2019).

86. B. R. Larsen, D. Di Bella, M. Glasius, R. Winterhalter, N. R. Jensen, J. Hjorth, Gas-phase OH oxidation of monoterpenes: Gaseous and particulate products. *J. Atmos. Chem.* **38**, 231–276 (2001).

87. B. Nozière, I. Barnes, K. H. Becker, Product study and mechanisms of the reactions of α-pinene and of pinonaldehyde with OH radicals. *J. Geophys. Res. Atmos.* **104**, 23645–23656 (1999).

88. Y. Zhao, D. G. Truhlar, The M06 suite of density functionals for main group thermochemistry, thermochemical kinetics, noncovalent interactions, excited states, and transition elements: Two new functionals and systematic testing of four M06-class functionals and 12 other functionals. *Theor. Chem. Acc.* **120**, 215–241 (2008).

89. T. H. Dunning, Gaussian basis sets for use in correlated molecular salculations. I. The atoms boron through neon and hydrogen. *J. Chem. Phys.* **90**, 1007–1023 (1989).

90. S. Grimme, S. Ehrlich, L. Goerigk, Effect of the damping function in dispersion corrected density functional theory. *J. Comput. Chem.* **32**, 1456–1465 (2011).

91. L. Goerigk, A. Hansen, C. Bauer, S. Ehrlich, A. Najibi, S. Grimme, A look at the density functional theory zoo with the advanced GMTKN55 database for general main group thermochemistry, kinetics and noncovalent interactions. *Phys. Chem. Chem. Phys.* **19**, 32184–32215 (2017).

92. I. M. Alecu, J. J. Zheng, Y. Zhao, D. G. Truhlar, Computational thermochemistry: Scale factor databases and scale factors for vibrational frequencies obtained from electronic model chemistries. *J. Chem. Theory Comput.* **6**, 2872–2887 (2010).

93. T. H. Dunning, K. A. Peterson, A. K. Wilson, Gaussian basis sets for use in correlated molecular calculations. X. The atoms aluminum through argon revisited. *J. Chem. Phys.* **114**, 9244–9253 (2001).

94. R. J. Bartlett, G. D. Purvis, Many-body perturbation theory, coupled-pair many-electron theory, and the importance of quadruple excitations for the correlation problem. *Int. J. Quantum Chem.* **14**, 561–581 (1978).

95. M. J. Frisch, G. W. Trucks, H. B. Schlegel, G. E. Scuseria, M. A. Robb, J. R. Cheeseman, G. Scalmani, V. Barone, G. A. Petersson, H. Nakatsuji, X. Li, M. Caricato, A. V. Marenich, J.

Bloino, B. G. Janesko, R. Gomperts, B. Mennucci, H. P. Hratchian, J. V. Ortiz, A. F. Izmaylov, J. L. Sonnenberg, D. Williams-Young, F. Ding, F. Lipparini, F. Egidi, J. Goings, B. Peng, A. Petrone, T. Henderson, D. Ranasinghe, V. G. Zakrzewski, J. Gao, N. Rega, G. Zheng, W. Liang, M. Hada, M. Ehara, K. Toyota, R. Fukuda, J. Hasegawa, M. Ishida, T. Nakajima, Y. Honda, O. Kitao, H. Nakai, T. Vreven, K. Throssell, J. A. Montgomery Jr., J. E. Peralta, F. Ogliaro, M. J. Bearpark, J. J. Heyd, E. N. Brothers, K. N. Kudin, V. N. Staroverov, T. A. Keith, R. Kobayashi, J. Normand, K. Raghavachari, A. P. Rendell, J. C. Burant, S. S. Iyengar, J. Tomasi, M. Cossi, J. M. Millam, M. Klene, C. Adamo, R. Cammi, J. W. Ochterski, R. L. Martin, K. Morokuma, O. Farkas, J. B. Foresman, D. J. Fox. (Gaussian Inc., Wallingford, CT, 2016).

96. L. Vereecken, J. Peeters, The 1,5-H-shift in 1-butoxy: A case study in the rigorous implementation of transition state theory for a multirotamer system. *J. Chem. Phys.* **119**, 5159–5170 (2003).

97. C. Eckart, The penetration of a potential barrier by electrons. *Phys. Rev.* **35**, 1303–1309 (1930).

98. H. S. Johnston, J. Heicklen, Tunnelling corrections for unsymmetrical Eckart potential energy barriers. *J. Phys. Chem.* **66**, 532–533 (1962).

99. B. Nozière, L. Vereecken, Direct observation of aliphatic peroxy radical autoxidation and water effects: An experimental and theoretical study. *Angew. Chem. Int. Edit.* **58**, 13976–13982 (2019).

100. M. E. Jenkin, R. Valorso, B. Aumont, A. R. Rickard, Estimation of rate coefficients and branching ratios for reactions of organic peroxy radicals for use in automated mechanism construction. *Atmos. Chem. Phys.* **19**, 7691–7717 (2019).

101. L. Vereecken, J. Peeters, A structure-activity relationship for the rate coefficient of H-migration in substituted alkoxy radicals. *Phys. Chem. Chem. Phys.* **12**, 12608–12620 (2010).

102. A. Novelli, C. Cho, H. Fuchs, A. Hofzumahaus, F. Rohrer, R. Tillmann, A. Kiendler-Scharr, A. Wahner, L. Vereecken, Experimental and theoretical study on the impact of a nitrate group on the chemistry of alkoxy radicals. *Phys. Chem. Chem. Phys.* **23**, 5474–5495 (2021).

103. H. C. Knap, S. Jørgensen, Rapid hydrogen shift reactions in acyl peroxy radicals. *J. Phys. Chem. A* **121**, 1470–1479 (2017).

104. S. Y. Wang, S. M. McNamara, C. W. Moore, D. Obrist, A. Steffen, P. B. Shepson, R. M. Staebler, A. R. W. Raso, K. A. Pratt, Direct detection of atmospheric atomic bromine leading to mercury and ozone depletion. *Proc. Natl. Acad. Sci. U.S.A.* **116**, 14479–14484 (2019).
